# Quantifying CDK inhibitor selectivity in live cells

**DOI:** 10.1038/s41467-020-16559-0

**Published:** 2020-06-02

**Authors:** Carrow I. Wells, James D. Vasta, Cesear R. Corona, Jennifer Wilkinson, Chad A. Zimprich, Morgan R. Ingold, Julie E. Pickett, David H. Drewry, Kathryn M. Pugh, Marie K. Schwinn, Byounghoon (Brian) Hwang, Hicham Zegzouti, Kilian V. M. Huber, Mei Cong, Poncho L. Meisenheimer, Timothy M. Willson, Matthew B. Robers

**Affiliations:** 1 Structural Genomics Consortium, UNC Eshelman School of Pharmacy, University of North Carolina at Chapel Hill, Chapel Hill, NC 27599 USA; 2 Promega Corporation, 2800 Woods Hollow Road, Madison, WI 53711 USA; 3 Target Discovery Institute, Nuffield Department of Medicine, University of Oxford, Oxford, UK; 4 Structural Genomics Consortium, Nuffield Department of Medicine, University of Oxford, Oxford, UK

**Keywords:** Kinases, Clinical pharmacology

## Abstract

Concerted multidisciplinary efforts have led to the development of Cyclin-Dependent Kinase inhibitors (CDKi’s) as small molecule drugs and chemical probes of intracellular CDK function. However, conflicting data has been reported on the inhibitory potency of CDKi’s and a systematic characterization of affinity and selectivity against intracellular CDKs is lacking. We have developed a panel of cell-permeable energy transfer probes to quantify target occupancy for all 21 human CDKs in live cells, and present a comprehensive evaluation of intracellular isozyme potency and selectivity for a collection of 46 clinically-advanced CDKi’s and tool molecules. We observed unexpected intracellular activity profiles for a number of CDKi’s, offering avenues for repurposing of highly potent molecules as probes for previously unreported targets. Overall, we provide a broadly applicable method for evaluating the selectivity of CDK inhibitors in living cells, and present a refined set of tool molecules to study CDK function.

## Abstract (web summary)

Cyclin-dependent kinase (CDK) inhibitors are widely used both in the clinic and for basic research aimed at dissecting the specific cellular functions of specific CDKs. Here, the authors report the development of a panel of fluorescent reporter probes and provide a comprehensive profile of the inhibitory activity of several CDK inhibitors towards all 21 CDKs in living cells.

## Introduction

Kinases represent the broadest class of intracellular enzymes in human cells, regulating critical nodes in signal transduction. As dysregulated kinase activity is common in a variety of cancers and immune diseases, small-molecule kinase inhibitors have emerged as one of the most successful modalities for drug development in the 21st century^1,2^. For example, cyclin-dependent kinases (CDKs) have been validated as oncogenic drivers in solid tumors^3^. The CDK family comprises 21 phosphotransfer enzymes with diverse cellular functions. CDK1, −2, −4 and −6 play key roles in the regulation of the eukaryotic cell cycle, CDK8–9 and −19 are involved in regulation of gene transcription^4,5^, while CDK7 has broader roles in both processes^6^. CDK activity is tightly regulated by intracellular protein-protein interactions, most critically with cyclin proteins. Many of the CDKs require heterodimerization with a cyclin protein to form an active enzyme^7^. This regulation is dynamic, as CDK/cyclin interactions oscillate depending on the cell cycle, providing a unique layer of complexity to intracellular signaling mediated by this kinase subfamily^8^. While knowledge of the regulatory role of the cell cycle and transcriptional CDKs has been extensively studied, the majority of the CDK family enzymes have unknown roles in cell physiology (most notably CDK5, −10, −11, 14–18, and −20). Nonetheless, the recent clinical advancement of dual CDK4/6 inhibitors for treatment of HER2 negative breast cancer has amplified broader interest in exploring the therapeutic potential of the established and understudied CDKs with small-molecule inhibitors^9,10^.

The vast majority of CDK inhibitors have been designed to occupy the nucleotide co-substrate (ATP) binding pocket^5,10^. As the catalytic pocket across the CDK enzyme family is highly conserved, the development of CDK inhibitors (CDKi’s) with isozyme selectivity is technically challenging. Moreover, the high concentration of intracellular ATP (varying between 1 and 10 mM and surpassing enzyme *K*_m_ by orders of magnitude) yields an unpredictable microenvironment for achieving competitive inhibition of CDK enzymatic activity^11–13^. While the CDK field is replete with ATP-competitive inhibitors with potent activity against the purified enzymes or kinase domains in cell-free biochemical assays, there remains a lack of well-characterized inhibitors with potent and selective pharmacology against each of the CDKs in live cells. The dearth of robust target engagement assay technologies that allow for an assessment of CDKi potency and selectivity within intact, living cells has represented a key technical limitation. Cellular methods for evaluating CDK isozyme pharmacology are generally limited to substrate phosphorylation analyses (e.g., Western blot from cell extracts), but such approaches suffer from the redundancy of CDK phosphotransfer activity across known substrates. For example, although phosphorylation of retinoblastoma protein (Rb) is a commonly used biomarker of CDK activity, the protein serves as a substrate for cell-cycle regulatory CDKs including CDK1, −2, −4, −5, and −6^14,15^. Beyond the cell-cycle-regulatory CDKs^16^, other isozymes in the family lack functional annotation and known substrates for intracellular phosphorylation analysis. Thus, evaluating the intracellular pharmacology of individual CDKs represents a major challenge across the family and leaves the understanding of inhibitor selectivity incomplete.

The pharmacological activity of CDKi’s is predicated on their physical engagement with cellular targets. Accordingly, target engagement potency will generally correlate quantitatively with potency of intracellular kinase inhibition^11,12,17^. Therefore, in the absence of isozyme-specific functional assays, cellular target engagement assays represent an ideal surrogate for evaluating inhibitor selectivity. Ideally, CDKi selectivity should be queried in a unified target engagement format, wherein occupancy is quantifiable in the presence of cyclin partners and other cellular factors that are known drivers in compound pharmacology for this kinase subfamily.

Here, we describe a comprehensive and systematic method to quantify target occupancy of CDKi’s in live cells for the complete CDK family. We use this method to perform an evaluation of intracellular target engagement selectivity for 46 CDKi’s comprising a collection of clinically-advanced compounds and recently published chemical tools. To evaluate CDK potency and selectivity in a physiological setting, we developed a panel of cell-permeable energy transfer probes that allow for quantitative evaluation of CDK fractional occupancy inside intact, living HEK-293 cells by Bioluminescence Resonance Energy Transfer (BRET) with CDK/NanoLuc fusion proteins^18^. Our results identified small-molecule CDKi’s with strong isozyme selectivity within cells, supporting their use as chemical tools. In contrast, we determined that a number of previously reported “selective” CDKi’s did not maintain their putative CDK selectivity profiles when evaluated in live cells. Surprisingly, a subset of this chemical matter, including a panel of well-studied clinically-advanced CDKi’s, can be repurposed as chemical probes for understudied CDK isozymes. Real-time analysis of target occupancy also revealed that CDK inhibitors may show surprisingly durable inhibition (i.e., long residence time) in live cells, resulting in a remarkable shift in the selectivity profile over time. The methods described herein can be applied to the evaluation of small-molecule inhibitors of all CDK family members. This analysis can thus serve as an adaptable workflow to evaluate CDKi selectivity potential in a variety of cell types and experimental systems to support discovery of new medicines. The resulting comprehensive analysis of CDK inhibitor activity in living cells is intended to provide a template for optimizing drug candidates and selecting chemical probes for experimental pharmacology.

## Results

### Cell-permeable energy transfer probes for all 21 human CDKs

To date, cell-free enzymatic or kinase binding assays have been used to successfully annotate the potency and selectivity of small-molecule CDKi’s^19,20^. Although robust and scalable in screening, these cell-free kinase assays do not query engagement in the presence of the cellular milieu (e.g., physiological ATP and the full complement of partners), and have therefore often revealed divergent pharmacology to that observed in cells^11,12,21^. The disconnect between biochemical and cell-based kinase potency^11,12^ has led to the development of more advanced techniques that allow for a systematic characterization of target occupancy in cell extracts via chemoproteomics^22^ and photoaffinity probes^23^. Such methods represent key technological advancements for the CDK field, but are generally incompatible with intact cell analyses. As kinase pharmacology is often impacted by the composite effect of the intracellular milieu, target occupancy measured in live cells may fundamentally differ from that observed in lysates or in purified systems^11,12^. For this reason, we elected to develop a comprehensive and unified method to measure the intrinsic CDKi affinity and selectivity across all 21 family members within live cells.

**Fig. 1:**
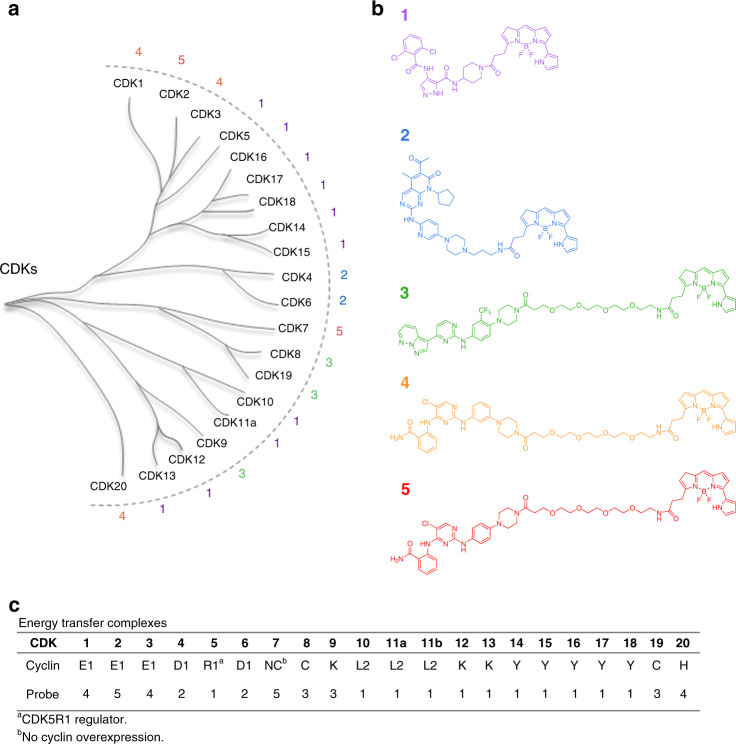
Comprehensive energy transfer system for all 21 human CDKs in live cells. **a** CDK phylogenetic tree^4,5,8^. **b** Cell-permeable energy transfer probes used to comprehensively profile CDK engagement in live cells. **c** Key components for each CDK assay are summarized and described in full in Supplementary Table 2. For simplicity, only CDK11A will be represented in the dendrograms throughout this manuscript. However, both CDK11A and CDK11B isoforms were evaluated.

Our groups previously collaborated to develop a collection of broad-spectrum energy transfer probes to query target occupancy for 178 kinases in live, intact cells^24^. With live cells expressing the selected NanoLuc(Nluc)/kinase fusion protein, this BRET method allows for scalable detection of target engagement using a simple ratiometric analysis of acceptor (red channel) and donor (blue channel) luminescence. In a competitive displacement format, target fractional occupancy results are quantitative when energy transfer probes are introduced at or below *K*_d_-apparent. Under such conditions, target engagement potency correlated quantitatively with potency of intracellular kinase inhibition, thus providing a predictive approach to evaluating kinase inhibitor selectivity in cells. Although the probe set covered 178 kinases, coverage over the CDK family was limited to only four members^24^. To adapt the method to cover the CDK family comprehensively, we employed a two-fold strategy wherein novel energy transfer probes were developed either from known CDKi’s or by optimization of known broad-spectrum ATP-competitive kinase inhibitors. Each probe was optimized for binding to their target CDKs by installation of a functional group for dye conjugation as well as selection of the linker between the binding moiety and the fluorophore (Supplementary Fig. 1). Each bifunctional energy transfer probe was screened for binding across the entire panel of 21 CDK/Nluc fusion proteins (Supplementary Table 1). Those probes demonstrating the highest specific energy transfer signals for each individual CDK were selected for further characterization in dose-response and competitive displacement experiments to optimize probe conditions for quantitation (Supplementary Figs. 2–5). This approach yielded 5 optimized energy transfer probes with sufficient performance to enable live-cell assays for 21 CDKs in a competitive displacement format (Fig. 1, Supplementary Table 2).

The structure of the five energy transfer probes is depicted in Fig. 1b. Briefly, probe 1 was developed from promiscuous CDK inhibitor AT7519^25,26^, which enabled assays for 11 CDKs including the complete TAIRE subfamily (CDKs14 −18). Robust assays for CDK4 and CDK6 were enabled by probe 2, which was developed from the FDA-approved drug palbociclib^27^. GW779439 was discovered during review of the published kinase inhibitor set 2 (PKIS2) dataset^28^, and probe 3 was developed from this scaffold to enable robust assays for CDK8, CDK9, and CDK19. Lastly, optimization of inhibitors based on the CTx-0294885^24^ scaffold yielded probes 4 and 5, which collectively enabled assays for CDK1, CDK2, CDK3, CDK7, and CDK20. For the majority of CDKs, the resulting assays proved suitable in screening environments, with Z′ values > 0.5 (Supplementary Table 2). For CDK12 and CDK13, energy transfer signals were more modest than the remaining family (Supplementary Fig. 2), potentially limiting their use to measurements of compound potency in dose-response experiments (as reported herein).

The enzymatic activity of many CDKs is known to be modulated in cells by the specific cyclin or regulatory partner to which they are complexed^8^. We therefore implemented an assay design that would allow evaluation of compound pharmacology based on CDK/cyclin interactions. Thus, in addition to evaluating the CDK-Nluc  fusions in both N- and C-terminal orientations (examples provided in Supplementary Fig. 6), we also defined the assay systems by co-expression of an excess of specific cyclins and regulatory partners. Cyclin genes were selected based on commonly reported CDK interactions^4,5,8^. Although not all described CDK/cyclin interactions were evaluated, the design of this assay method will allow for alternate CDK/cyclin interactions to be queried in a simplified workflow. For the majority of the CDKs, co-expression of an excess of a known^8^ cyclin partner potentiated the energy transfer signal, providing support that the CDK population was shifted toward the selected cyclin pair (Supplementary Figs. 7–9). Furthermore, engagement potency for some compounds shifted dramatically in response to cyclin overexpression. For example, in the case of CDK2, co-expression of cyclin E1 increased the potency of both dinaciclib and RGB-286638 by nearly 2 orders of magnitude compared with co-expression with cyclin A1 (Supplementary Fig. 10). In contrast, NVP-LCQ-195 and SB-1317 showed similar potency regardless of which cyclin was co-expressed (Supplementary Fig. 10). These results suggest that compound pharmacology may be generally impacted by intracellular cyclin interactions, and that such shifts are chemotype-dependent.

To confirm whether plasmid-based overexpression conditions promote formation of intended CDK/cyclin complexes, a NanoLuc binary complementation system (NanoBiT^29^) was evaluated for CDK2/cyclin E1 (Supplementary Fig. 11). The luminescence generated using the NanoBiT complementation system supports formation of the CDK2-cyclin complex as well as ternary complex formation with energy transfer probe (Supplementary Fig. 12). Moreover, target engagement results with dinaciclib using the NanoBiT system closely match those using the CDK2-NanoLuc/cyclin E1 co-expression. Thus, co-expression of cyclin E1 has a pronounced effect on engagement potency for CDK2 compared with CDK2 expression alone (Supplementary Fig. 12). These results are consistent with the general observation that energy transfer is potentiated upon co-expression of CDK and cyclin, thus supporting formation of the intended CDK/cyclin interaction under these experimental conditions.

### CDKi panel and criteria for determining intracellular selectivity

Despite the literature being rife with CDK inhibitors, very few CDKi’s have been profiled for target selectivity in living cells^19,20,22^. As such, the chemical landscape of CDK inhibition has yet to be systematically defined. To define the CDK selectivity profiles in live cells, we assembled a set of 46 commercially-available CDKi’s that represent broad chemical diversity (Supplementary Data 1). The panel included the 3 FDA-approved CDK inhibitors, 18 drugs in advanced clinical trials, and tool molecules that had been described in the literature. In this panel we included three kinase inhibitors with reported collateral engagement of CDKs in a cell-free or lysate-based format (dabrafenib, BDP5290, and momelotinib)^22,30–32^. These compounds were selected to evaluate CDKs as potential off-target liabilities in a live-cell context. To assemble a comprehensive intracellular profile for all 46 CDKi’s, each compound was initially profiled across all 21 CDK/cyclin complexes in live cells at 10 µM, using 50% occupancy as a cutoff for follow-up potency (IC_50_) determination. Supplementary Data 2 and 3 summarize the potencies for all molecules conforming to stringent intracellular occupancy (≥50% at 10 μM) and potency (<1 µM) criteria.

### Verification of CDKi selectivity for CDKs 4/6, −7, and −9

**Fig. 2:**
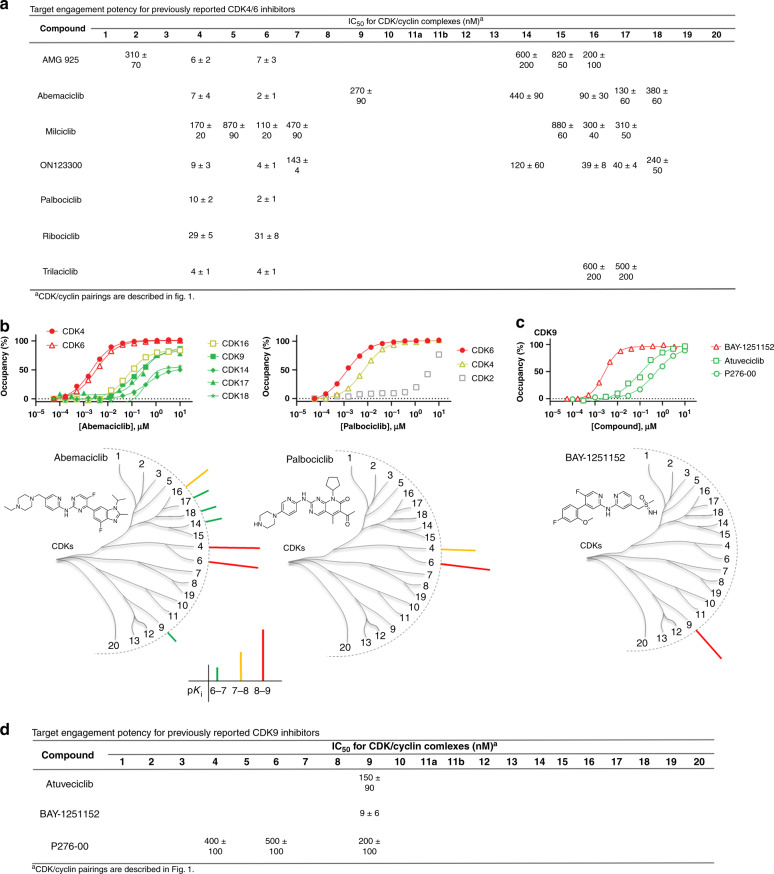
Selective CDK4/6 and CDK9 inhibitors. **a** Live cell engagement potency for CDK4/6 selective probes. Reported IC_50_ data with values <1 µM. Blank cells represent IC_50_ values that failed to meet our criteria of potency (<1 µM) or occupancy (≥50% at 10 µM). Reported IC_50_ data are the mean of 3 independent experiments ± S.E.M. (Supplementary Data 2). **b** Upper: Target engagement of abemaciclib and palbociclib. A representative single technical replicate (*n* = 1) of live cell target occupancy results from 3 independent experiments (Supplementary Data 2). For palbociclib, CDK2 occupancy is included for comparative reference. Lower: Compound structures and dendrogram-based illustration of engagement selectivity for abemaciclib and palbociclib against the complete CDK family. **c** Upper: Target engagement of CDK9-selective inhibitors. A representative single technical replicate (*n* = 1) of target occupancy results from 3 independent experiments (Supplementary Data 2). Lower: Compound structure and dendrogram-based illustration of engagement selectivity for BAY-1251152. **d**. Engagement potency for CDK9-selective inhibitors. Reported IC_50_ data are the mean of 3 independent experiments ± S.E.M. (Supplementary Data 2). Blank cells represent IC_50_ values that failed to meet our criteria of potency (<1 µM) or occupancy (≥50% at 10 µM). Source data are provided as a Source Data file.

CDK4 and CDK6 are two closely related family members that control transition from G1 to S phase of the cell cycle, and are established oncogenic drivers in a variety of solid tumors^33^. Accordingly, drug development programs have yielded three dual CDK4/6 inhibitors (abemaciclib, palbociclib, and ribociclib) that have been FDA-approved for treatment of breast cancer. As validation of our approach, we generated the full CDK profile of these drugs and other known CDK4/6 inhibitors in our live-cell energy transfer system to compare with their reported clinical pharmacology. Eight CDK4/6 inhibitors were evaluated against all 21 CDKs (Fig. 2a and Supplementary Data 2 and 3). All three FDA approved CDKi’s potently engaged CDK4/6 in the live HEK-293 cell assays. Abemaciclib showed a target engagement potency at CDK4/6 that agreed closely with its reported cellular potency in MCF7 cells^34^. However, abemaciclib also showed collateral engagement of CDK9, and exhibited pan-TAIRE family activity (CDK14 and CDKs16−18), (Fig. 2b)^35^. Palbociclib and ribociclib were more selective for CDK4/6, with minimal engagement against the remaining family (Fig. 2 and Supplementary Fig. 13). Other CDK4/6 inhibitors showed lower levels of cellular selectivity: AMG-925 and ON123300 potently engaged CDK4/6 (Supplementary Fig. 14), but collaterally engaged a number of other family members including those directly involved in cell cycle regulation. In cells, CDK4/6 were potently engaged by a number of CDKi’s that were previously annotated against other family members. For example, milciclib engaged CDK4/6 with strongest potency within the family, despite being described as a pan-CDKi with modest selectivity for CDK2 in cell-free formats^10,20^.

For highly selective CDK4/6 inhibitors, target occupancy should be commensurate with intracellular enzymatic inhibition. To evaluate this relationship, target engagement and endogenous substrate phosphorylation (phospho-Rb) analyses were performed in MCF7 cells for the CDK4/6-selective inhibitors. CDK4 and CDK6 target engagement assays were adapted into MCF7 cells using conditions similar to those developed for HEK-293. Potency of endogenous phospho-Rb (S807/811) inhibition was measured using a homogenous luminescent immunoassay^36^. In MCF7, a strong correlation (*R*^2^ = 0.99) between rank-order potencies of CDK6 engagement and reduction of endogenous phospho-Rb was observed for all three of the FDA-approved CDK4/6 inhibitors as well as trilaciclib (a phase 2 clinical CDKi) (Supplementary Fig. 15). Engagement potencies were modestly left-shifted compared with functional inhibition, possibly due to the differences in cell treatment conditions (e.g., the extended duration of compound treatment required for phospho-Rb inhibition). Although such correlations may not always be observed for less selective CDKi’s, these results support the translational capacity for target occupancy analysis when evaluated in a unified pathophysiological setting.

The role of CDK9 is in transcriptional regulation and its dysregulation has been implicated in a variety of human pathologies^37^. Our results demonstrate that BAY-1251152 and atuveciclib are inhibitors with strong and selective engagement to CDK9 in cells. Both compounds were highly selective for CDK9 over other members of the family. Our analysis also revealed that P276-00, a reported pan-CDKi, engaged CDK9 in live cells with modest selectivity compared with the remaining family (Fig. 2d and Supplementary Fig. 16). Among the CDKi’s in our panel, BAY-1251152 demonstrated the strongest target affinity and selectivity (Fig. 2c) and is recommended as a tool compound for selective modulation of CDK9 function in cellular studies. The results with BAY-1251152 were corroborated using HEK-293 cells edited via CRISPR/ Cas9 to introduce the HiBiT reporter tag^38^ onto CDK9 at endogenous genomic loci. BAY-1251152 potencies were in close agreement for both ectopic expression of CDK-NanoLuc and endogenously edited CDK/NanoBiT reporters (Supplementary Fig. 17).

**Fig. 3:**
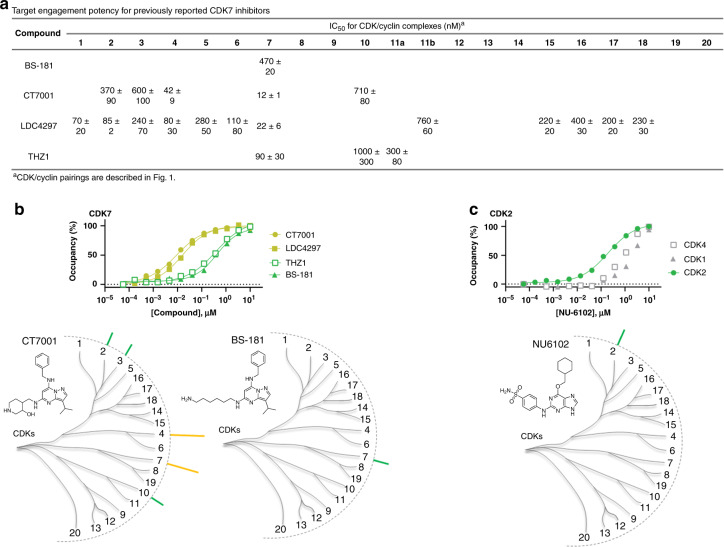
Selective CDK7 and CDK2 inhibitors. **a** Live cell engagement potency for CDK7 selective probes. Reported IC_50_ data are the mean of 3 independent experiments ± S.E.M. (Supplementary Data 2). Blank cells represent IC_50_ values that failed to meet our criteria of potency (< 1 µM) or occupancy (≥ 50% at 10 µM). **b** Upper: Target engagement of CDK7-selective inhibitors. A representative single technical replicate (*n* = 1) of live cell target occupancy results from 3 independent experiments (Supplementary Data 2). Lower: Compound structures and dendrogram-based illustration of engagement selectivity for CT7001 and BS-181 against the complete CDK family. **c** Upper: Target engagement of NU6102 against CDK2. A representative technical replicate (*n* = 1) of target occupancy results from 3 independent experiments is shown (Supplementary Data 2). Potency of putative targets CDK1 and CDK4 are shown for comparison. Lower: Chemical structure and dendrogram-based illustration of engagement selectivity for NU6102. Source data are provided as a Source Data file.

CDK7 has been identified as a promising drug target due to its dual function in controlling the cell cycle and transcription, which has led to several inhibitors undergoing evaluation in clinical trials as anticancer therapies^39–41^. Molecules based on the pyrazolopyrimidine scaffold have been reported as potent inhibitors of CDK7. Surprisingly, in our analysis, pyrazolopyrimidine CT7001^42^ showed potent engagement of CDK4 in addition to CDK7 (Fig. 3a, b, Supplementary Data 2 and 3), with modest selectivity over other members of the family. LDC4297^43^, a structurally related CDK7 inhibitor, engaged CDK7 with strongest potency within the family, but was less selective than originally reported and showed collateral engagement to CDK1−6, and exhibited pan-TAIRE-family activity (CDK15–18), (Supplementary Fig. 18). In our cellular analysis, the pyrazolopyrimidine BS-181 engaged CDK7 with modest potency (470 nM) with negligible occupancy at other family members at concentrations under 1 µM (Fig. 3a, b). BS-181 should therefore be considered among the best-in-class selective CDK7 probes in our panel.

CDK7 contains a reactive cysteine (C312) located outside the nucleotide pocket that can be targeted with covalent inhibitors. Gray and coworkers exploited this mechanism to develop THZ1, a potent covalent inhibitor of CDK7 with efficacy in multiple cell models^6,44^. We evaluated THZ1 at our standard 2 h incubation time (Fig. 3a and Supplementary Fig. 18), as well as an extended 6 h duration in live cells (Supplementary Fig. 19). Only modest selectivity of THZ1 was observed for CDK7 after 2 h of incubation with cells (Fig. 3a). An extended 6 h incubation enhanced the engagement potency to CDK7 (Supplementary Fig. 19), matching closely with antiproliferative potency of THZ1 in Jurkat cells^6,44^. Thus, our findings corroborate time-dependent engagement of CDK7 by THZ1^6^ and support its potential utility as a CDK7 tool compound after extended incubation times.

### CDK1 and CDK2 inhibitors are poorly selective

As critical modulators of cell cycle progression, CDK1 and CDK2 have been targets for development of cancer drugs^3^. We evaluated the intracellular selectivity of a number of molecules reported as selective CDK2 or dual CDK1/2 inhibitors. A number of CDKi’s demonstrated intracellular affinity values for CDK1 and CDK2 below 100 nM, and a subset of these compounds had intracellular affinities approaching single-digit nM (AZD5597, dinaciclib, BS-194, CDKI-73, and RGB286638). Remarkably, all of the highest affinity CDK1/2 inhibitors collaterally engage other CDK family members. Comprehensive intracellular profiling of these potent CDK1/2 compounds revealed strong collateral engagement to other CDK’s, most notably the TAIRE subfamily (CDK14–18), (Supplementary Data 2 and 3). For example, CDKI-73 and RGB286638 (Supplementary Fig. 20) engaged the closely related CDK16 and CDK17 with strong intracellular affinity. Our data demonstrate that broad assessment of CDKi pharmacology in live cells is warranted, especially for compounds that advance to clinical development.

While high-affinity CDKi’s for CDK1/2 yielded strong engagement to other family members, some weaker affinity inhibitors also showed modest selectivity for CDK2 in cells. For example, NU6102 was selective for CDK2 over CDK4, with a relatively weak engagement of the remaining CDK family (Fig. 3c). Thus, although CDK1/2 are two of the most highly studied family members, none of the inhibitors tested were both potent and selective for these isozymes in cells.

### Repurposing CDK1/2 inhibitors for CDK8/19 in cells

In our comprehensive live-cell analysis, a number of the inhibitors in this study produced surprisingly strong engagement patterns to collateral CDKs. For example, pan-TAIRE family (CDK14–18) activity was observed for several CDK1/2 inhibitors (Supplementary Data 2 and 3). We therefore evaluated the possibility that a subset of CDKi’s may engage understudied CDKs with stronger intracellular affinity than their originally targeted family member, and if such molecules could be repurposed as probes for the lesser-studied family member.

**Fig. 4:**
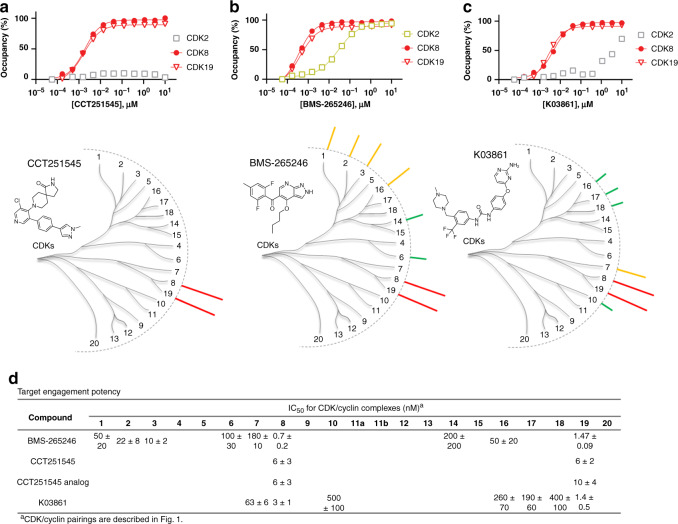
Selective dual inhibitors of CDK8/19. Target engagement at CDK8/19 (upper panels) with compounds CCT251545 (**a**), BMS-265246 (**b**), and K03861 (**c**). CDK2 is included in each graph for comparative reference. Each graph shows a single representative technical replicate (*n* = 1) from 3 independent experiments (Supplementary Data 2). Lower: Compound structures and dendrogram-based illustration of engagement selectivity for CCT251545, BMS-265246, and K03861 against the complete CDK family D. Representative live cell engagement potency for CDK8/19-selective compounds. Reported IC_50_ data are the mean of 3 independent experiments ± S.E.M. (Supplementary Data 2). Blank cells represent IC_50_ values that failed to meet our criteria of potency (<1 µM) or occupancy (≥50% at 10 µM). Source data are provided as a Source Data file.

The paralog kinases CDK8/19 were engaged by a number of CDKi’s with nanomolar intracellular affinity. CDK8/19 are closely related but relatively understudied members of the CDK family that have been identified as components of the mediator complex involved in global regulation of transcription in eukaryotic cells^45^ and are potential oncogenes in a subset of solid tumors^46^. Recently, two chemical probes have been described for CDK8/19^47,48^. CCT251545 (and a related analog) potently inhibited downstream CDK8/19 activity biomarkers with single-digit nanomolar potency^47,48^. Our live-cell occupancy results at CDK8 and CDK19 (≤10 nM *K*_d_-apparent for both CDK8 and CDK19) agreed closely with the these reported cellular potencies (Fig. 4, Supplementary Fig. 21, and Supplementary Data 2 and 3).

We also uncovered a number of compounds with unexpected selectivity for CDK8/19 (Fig. 4). The CDK2 inhibitor K03861^49,50^ engaged CDK8/19 in cells, with pronounced selectivity over other family members, including CDK2 (Fig. 4c, d). K03861 is a type II inhibitor that stabilizes the inactive DFG-out conformation of CDK2^49,50^. Comparable results were observed with the related chemotype AST-487 (albeit with more modest potency for CDK8/19). CDK8/19 has been reported to adopt a similar inactive conformation to accommodate type II inhibitors similar to that of CDK2^51^. However, intracellular engagement of type II inhibitors to CDK8/19 has not been reported previously. Our results identify K03861 and AST-487 as selective chemical probes for CDK8/19 within the family, and indicate that type II inhibition should be evaluated further as a potential strategy for mediator kinases.

Chemotypes structurally unrelated to K03861 showed similar patterns of selectivity to CDK8/19, offering additional opportunities for repurposing. Previously annotated as a CDK1/2 inhibitor^20^, BMS-265246 engaged CDK8/19 potently (<2 nM *K*_d_-apparent) and with >10-fold selectivity index over CDK1/2 (Fig. 4b, d). Similarly, CDK8/19 were among the most potent intracellular targets of flavopiridol (previously a clinical asset and described as a pan-CDKi), (Supplementary Data 2 and 3). Our results demonstrate that CDK8 and CDK19 are collateral targets of a number of mischaracterized CDKi’s in cells, and opportunities may exist for repurposing one or more of them as chemical probes of mediator kinase activity. Moreover, these results support that the composite effect of the intracellular milieu has a strong influence on CDKi pharmacology and that cellular profiling should accompany cell-free studies for determination of isozyme selectivity.

### Residence time as a CDK selectivity determinant

For evaluation of CDKi selectivity, steady-state analysis is standard practice. However, these equilibrium-based measurements may fail to accurately predict occupancy in vivo, where drug concentrations are highly dynamic^52,53^. In a dynamic open system, it is possible to achieve target selectivity via durable binding interactions that may not be evident under steady-state conditions^54^. The residence time (1/k_off_) of the target-ligand interaction is often a more accurate predictor of drug efficacy and pharmacodynamic effect^52^. It has been reported that some CDKi’s display protracted residence time in purified biochemical assays^55^. We therefore explored the possibility that CDKi’s may yield durable engagement, and kinetic selectivity under simulated open system conditions in living cells.

Among all 46 CDKi’s evaluated here, RGB286638 is among the most potent and broad coverage over the CDK family in live cells under steady-state conditions (Supplementary Data 2 and 3). However, residence times for this and many of the other CDKi’s in our panel have not been reported in cell-free or cellular contexts. Based on its broad coverage, RGB286638 served as an ideal candidate to evaluate a potential disconnect between target potency and residence time in cells. To query residence time as a potential selectivity determinant, CDK2, CDK6, and CDK7 were used based on their nearly identical intracellular affinity values with RGB286638 (12−16 nM). Residence time was determined via pre-equilibration with each target/CDKi combination at a near saturating concentration (~20-fold above *K*_d_-apparent as determined above under equilibrium conditions). This condition was selected to ensure adequate target occupancy prior to compound washout. Residence time was then evaluated by a rapid compound washout procedure, followed by addition of energy transfer probe 1. Under these conditions, the rate of the energy transfer signal increase serves as a direct proxy for the loss of the target-CDKi interaction^18,56,57^.

**Fig. 5:**
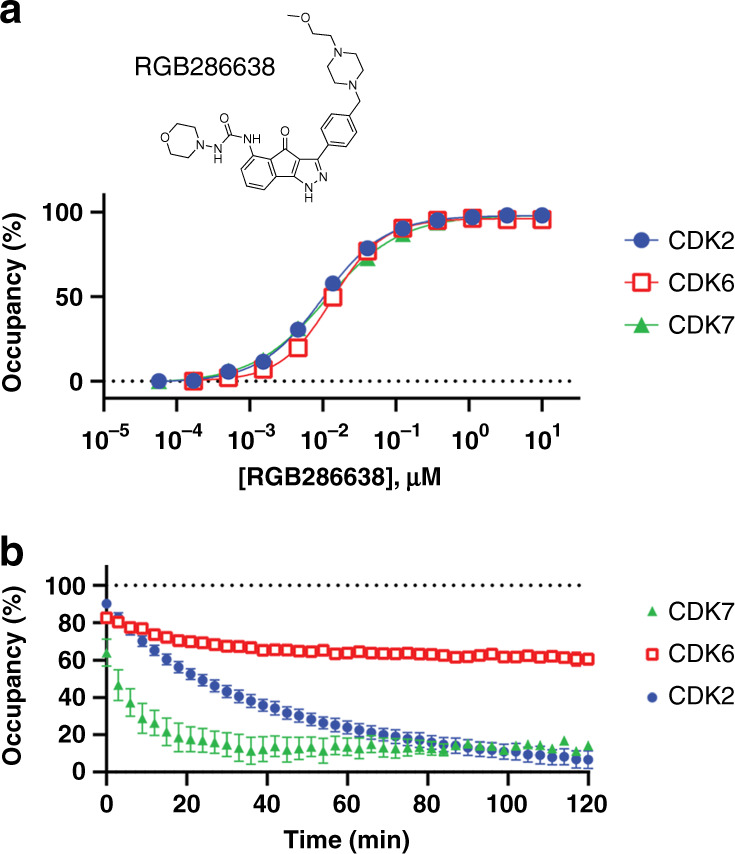
Pan-CDK inhibitor RGB286638 is kinetically-selective for CDK6 over CDK2/7 in cells. **a** Compound structure and steady-state target engagement of RGB286638 for CDKs 2, 6, and 7. A representative single technical replicate (*n* = 1) from 3 independent experiments is shown (Supplementary Data 2). **b** Residence time was measured by pretreating cells with compound (10−20-fold above the apparent *K*_d_ measured in **a**), followed by compound removal and introduction of energy transfer probe 1 at 1 µM. After 2 h of real-time analysis, CDK6 is >50% occupied while CDK2 and CDK7 are fully dissociated. Apparent residence times were 41 min for CDK2 and 8 min for CDK7, while residence time at CDK6 was too long to quantify after 2 h of analysis. Data are the mean ± S.E.M. of three independent experiments. Source data are provided as a Source Data file.

In contrast to steady-state analysis of RGB286638 (which yielded similar CDKi potencies for CDK2, CDK6, and CDK7, Supplementary Data 2 and 3), real-time analysis revealed robust durability to only CDK6 (Fig. 5b and Supplementary Fig. 22). After 2 h of occupancy analysis following compound washout, CDK6 remained >50% occupied by RGB286638, while CDK2 and CDK7 were fully dissociated. Apparent residence times for CDK2 and CDK7 were 41 min and 8 min, respectively, while the CDK6 dissociation rate with RGB286638 was too slow to quantify under these experimental conditions. This pattern was surprising, given the similar affinities observed for all three CDKs under steady-state conditions in cells (Fig. 5a). We therefore evaluated the apparent rate of equilibration of RGB286638 to corroborate the protracted residence time at CDK6. Although this equilibration rate may be impacted by the composite effect of compound permeability and interference from endogenous factors such as ATP, the analysis allows for a qualitative comparison of relative equilibration kinetics. Compared with CDK7, the rate of CDK6 engagement was relatively slow, thus supporting reduced apparent association and dissociation kinetics (Supplementary Fig. 23). These results support a mechanism of kinetic selectivity for CDK6 over CDK2 and CDK7 in living cells. However, it is possible that the durable occupancy and slow engagement of RGB286638 with CDK6 may be impacted by intracellular compound partitioning and/or rebinding, and therefore only observed in cell culture systems^58,59^. To rule out the potential impacts of cellular physiology on engagement kinetics and residence time, a cell-free biophysical evaluation is warranted to quantify the intrinsic rates of engagement to CDK6 and the remaining family members. These preliminary intracellular results encourage broader assessment of CDKi residence time as a selectivity determinant.

## Discussion

We have developed a panel of cell-permeable energy transfer probes to enable a quantitative evaluation of target occupancy against all 21 human CDKs in live, intact cells. We report a summary of intracellular target engagement potencies for 46 advanced CDKi’s including many with clinical activity (Supplementary Data 2 and 3). For CDKi’s targeting CDKs 4/6, −7, and −8/19, our target occupancy analysis agreed closely with potency at known biomarkers of intracellular CDK activity^6,34,44,47,48^. Moreover, rank-order potencies of engagement and reduction of substrate phosphorylation in MCF7 cells were in agreement for highly selective CDK4/6 inhibitors. Corroborating earlier studies on histone deacetylases (HDACs) and kinases^12,18^, these observations support that potency of target engagement can accurately correlate with potency of target inhibition when each are assessed in a common pathophysiological setting. Furthermore, results with endogenously edited cells matched closely with ectopic expression of CDK/cyclin complexes, further supporting that this target engagement method serves as a suitable proxy for interrogating CDKi selectivity in the absence of cellular assays that measure intrinsic CDK enzyme activity. As the majority of the CDK family have limited functional annotation (and generally lack specific substrates or biomarkers for intracellular activity), the ability to quantify target occupancy for all 21 CDKs in a unified format will be broadly enabling.

Our analysis cumulatively uncovered potent and selective inhibitors that have utility as selective tool molecules to modulate individual CDKs or paralog isozymes in live cells (Table 1). For CDK4/6 and CDK9, a subset of CDKi’s exhibited nanomolar affinity and robust indexes of selectivity. For CDK2, CDK3, and CDK7, only modestly selective inhibitors with reduced intracellular potency were observed. Although a portion of our intracellular analysis corroborated CDK profiles from cell-free biochemical systems, for many compounds we observed a striking pattern of intracellular selectivity that diverged from the cell-free analyses. For example, numerous inhibitors of CDK4/6 and CDK7 were more promiscuous in cells than previously reported (engaging a number of collateral CDKs and exhibiting pan-TAIRE activity). Conversely, a number of pan-CDKi’s had surprisingly narrow spectrum of activity in cells and were even marginally isozyme selective (e.g., milciclib for CDK4/6 and P276-00 for CDK9). The reduced spectrum of intracellular activity was not unique to the molecules only annotated as CDK family inhibitors. For example, in live cells, dabrafenib (a BRAF inhibitor) and BDP5290 (a ROCK kinase inhibitor) only weakly engaged CDKs at concentrations up to 10μM, despite reported collateral engagement to CDKs in cell-free and lysate-based kinase profiling experiments^22,30,31^. In this context, live-cell CDK profiling may become valuable for de-risking late-stage kinase inhibitors in a clinical setting.

**Table 1:** Best CDKi’s identified in this study for selective target engagement in live cells.

CDK	Compound	On target potency^a^	Collateral CDK (potency)
CDK2	NU6102	290 nM	ST^b^
CDK3	RO-3306	200 nM	CDK7 (690 nM)
CDK4/6	Palbociclib	10 nM/2 nM	ST^b^
CDK4/6	Ribociclib	29 nM/31 nM	ST^b^
CDK7	THZ1	90 nM	CDK11a (300 nM)
CDK7	BS-181	470 nM	ST^b^
CDK9	BAY-1251152	9 nM	ST^b^
CDK9	Atuveciclib	150 nM	ST^b^
CDK8/19	CCT251545	6 nM/6 nM	ST^b^
CDK8/19	CCT251545 analog	6 nM/10 nM	ST^b^
CDK8/19	BMS-265246	0.7 nM/1.5 nM	CDK3 (10 nM)
CDK8/19	K08361	3 nM/1.4 nM	CDK7 (63 nM)
CDK12	THZ531	770 nM	ST^b^

^a^Potency represents the mean of 3 independent experiments. For S.E.M., see Supplementary Data 2 or 3.

^b^Sub Threshold: no collateral CDKs were detected with potency below the cutoff of 1 µM.

Strikingly, a number of CDKi’s had off-target profiles that warrant reclassification for understudied family members. For example, we identified BMS-265246 (previously a clinical asset) as a potent and selective inhibitor of mediator kinases CDK8/19 that is currently annotated as a CDK1/2 inhibitor^20^. Furthermore, potent intracellular engagement of a reported type II CDK2 inhibitor, K03861, was observed with CDK8/19. Further evaluation is therefore warranted to determine if K03861 engages CDK8/19 via a type II mechanism, and if type II inhibitors could be a productive strategy for inhibition of mediator kinase activity. Other pan-CDKi’s, notably flavopiridol, surprisingly engaged CDK8/19 with strong potency compared with other family members. Thus, these intracellular occupancy measurements support avenues for repurposing numerous mischaracterized CDKi’s as selective probes in live cells. Moreover, additional studies are warranted to evaluate the engagement of CDK8/19 as a mechanism of efficacy or adverse events for previous clinical assets such as BMS-265246 and flavopiridol.

Despite profiling a wide array of chemotypes, we were unable to identify selective tool molecules for some CDKs. For CDK12 and CDK13, few potent inhibitors were observed. Although 10 μM THZ531 occupied both CDK12 and CDK13, potent engagement (<1 μM) was only observed for CDK12. It is possible that extended incubation times may be required to determine the engagement potential of such covalent inhibitors. Although many compounds exhibited potent TAIRE family activity (CDK14–18), collateral engagement was observed for these compounds across other CDK family members. For CDK20 no molecule showed >50% engagement at 10 µM, except for CTx-0294885, the parent compound from which probes 4 and 5 was developed (Supplementary Fig. 3). Although our analysis failed to uncover selective modulators of these CDKs, the energy transfer-based probes developed in this work can be used to identify and optimize potential tool molecules for these understudied but important family members. Encouragingly, during the preparation of this manuscript a number of inhibitors were reported for understudied CDKs including the TAIRE subfamily^60^ as well as CDK11^61^. As our compound panel represents only a fraction of known CDKi’s, the results presented here suggest that comprehensive assessments of CDK target engagement are warranted as a standard practice for all novel tool compounds and promising clinical leads that are beyond the scope of this study. If such comprehensive analyses could be generated, live-cell CDK profiling results would serve as a complementary resource to existing databases based on cell extracts^22,23^ or biochemically-defined assay systems^20^.

We further extended the intracellular analysis of CDKi selectivity to a simulated open system, for evaluation of target residence time. Our results support that even the most potent and non-selective CDKi’s such as RGB286638 may durably engage only a subset of CDKs despite similar potencies in cells. These results support a potential disconnect between thermodynamic and kinetic selectivity in live cells for certain CDKi’s, as well as a method to optimize kinetic selectivity within the CDK family. Accordingly, real-time analysis of intracellular residence time may be critical to predicting CDKi selectivity in an in vivo setting where drug concentrations are highly dynamic and cannot be represented only at steady-state.

Based on the divergent CDK profiles observed in cells vs cell-free systems, an evaluation of CDKi selectivity in live cells may be warranted against the broader kinome as demonstrated previously for multi-kinase inhibitors^12^. As CDKi selectivity patterns may be influenced by cellular context, this workflow is designed to be readily adapted to evaluate target engagement in alternate cell models. This approach is therefore intended to serve as a template for querying intracellular selectivity for CDKi’s as drug leads and chemical probes for experimental pharmacology.

## Methods

### Cell transfections and BRET measurements

HEK-293 cells (ATCC) were cultured in DMEM (Gibco) + 10% FBS (Seradigm), with incubation in a humidified, 37 °C/5% CO_2_ incubator. N- or C-terminal NanoLuc/CDK fusions were encoded in pFN31K or pFC32K expression vectors (Promega), including flexible Gly-Ser-Ser-Gly linkers between NLuc and each full-length kinase. Cyclin or other regulatory protein expression vectors were encoded in pFN5K vectors (Promega). Optimal orientations for each construct are described in Supplementary Table 2. For cellular BRET target engagement experiments, HEK-293 were transfected with NLuc/target fusion constructs using FuGENE HD (Promega) according to the manufacturer’s protocol. Briefly, NLuc/target fusion constructs were diluted into either regulator expression vector or Transfection Carrier DNA (Promega) at a mass ratio of 1:9 (mass/mass), after which FuGENE HD was added at a ratio of 1:3 (µg DNA: µL FuGENE HD). 1 part (vol) of FuGENE HD complexes thus formed were combined with 20 parts (vol) of HEK-293 cells suspended at a density of 2 × 10^5^ per mL, followed by incubation in a humidified, 37 °C/5% CO_2_ incubator for 20 hr. BRET assays were performed in white, tissue-culture treated 96-well plates (Corning #3917) at a density of 2 × 10^4^ cells per well. All chemical inhibitors were prepared as concentrated stock solutions in DMSO (Sigma-Aldrich) and diluted in Opti-MEM to prepare working stocks. Cells were equilibrated for 2 h with energy transfer probe and test compound prior to BRET measurements. Energy transfer probes were prepared at a working concentration of 20× in dilution buffer (12.5 mM HEPES, 31.25% PEG-400, pH 7.5). To measure BRET, NanoBRET NanoGlo Substrate and Extracellular NanoLuc Inhibitor (Promega) were added according to the manufacturer’s recommended protocol, and filtered luminescence was measured on a GloMax Discover luminometer equipped with 450 nm BP filter (donor) and 600 nm LP filter (acceptor), using 0.5 s integration time. BRET values are calculated by dividing the acceptor luminescence by the donor luminescence. Milli-BRET (mBRET) units (mBU) are calculated by multiplying the raw BRET values by 1,000.

### Determination of energy transfer probe affinity

For energy transfer probe dose-response experiments, energy transfer probes were added to cells as an 11-point dilution series starting at a maximum final concentration of 1 µM. To determine apparent tracer affinity, mBRET values were plotted as a function of energy probe concentration, and probe affinity values (EC_50_) were determined using the sigmoidal dose-response (variable slope) equation available in GraphPad Prism, Eq. (1);

1 $$Y = {\mathrm{Bottom}} + \left( {{\mathrm{Top}} {\hbox{-}} {\mathrm{Bottom}}} \right)/\left( {{\it{1}} + {\it{10}}^ \wedge \left( {\left( {{\mathrm{LogEC}}_{50}-X} \right) \ast {\mathrm{HillSlope}}} \right)} \right)$$

where *X* = energy transfer probe concentration and *Y* = mBRET.

### Determination of test compound occupancy and potency

To determine test compound occupancy, the energy transfer probes were added to the cells at concentrations optimized for each target, as described in Supplementary Table 2, along with a single dose or dilution series of the test compound (vide infra). BRET values were converted to occupancy (%) according to Eq. (2) below

2 $${\mathrm{Occupancy}}\,( {\it{\% }}) = [ {{\it{1}} - ( {X - Z} )/( {Y - Z} ) ] {\ast} {\it{100}}}$$

where *X* = mBRET in the presence of the test compound and the energy transfer probe, *Y* = mBRET in the presence of only energy transfer probe, and *Z* = mBRET in the absence of energy transfer probe and test compound. To determine potency, occupancy (%) values were then plotted as a function of test compound concentration, and the data were fitted to Eq. (1) to determine the IC_50_ value.

### Test compound formulation and testing strategy

Test compounds were sourced from common chemical vendors (Supplementary Data 1). All test compounds were formulated at 10 mM in DMSO, after which they were diluted in OptiMEM to prepare a 10X working solution for testing in the BRET assay. Compounds were initially screened for activity at a dose of 10 µM in two independent experiments. Compounds displaying >50% occupancy for the mean of the two independent experiments were further characterized in dose-response experiments to determine the potency. The potency (IC_50_) was determined using an 11-point dilution series (maximum dose of 10 µM) in two independent experiments. Compounds that reproducibly displayed potency values ≤ 1 µM were considered hits and were reported. Potency for compounds that did not reproducibly demonstrate values ≤ 1 µM were not reported.

### Evaluation of intracellular residence time

Residence time was qualitatively assessed via a cellular washout approach, where delayed energy probe association is used as an indirect measurement for the durability of target/compound engagement. Cells were transfected as described above and pre-equilibrated with a dose of test compound 20-fold above the apparent potency value (IC_50_, vide supra) as indicated in the figure legends. After 2 h of incubation at 37 °C, the medium was aspirated from the cells and replaced with fresh OptiMEM containing NanoBRET NanoGlo Substrate and Extracellular NanoLuc Inhibitor. Energy transfer probe 1 was added at 1 μM and the BRET was measured kinetically at 3-min intervals. Occupancy values were plotted as a function of time. Apparent residence times for RGB286638 to CDKs (residence time, tau) were calculated using Supplementary Eq. (2). Apparent residence time for CDK6 was too long to measure under these assay conditions.

### Reporting summary

Further information on research design is available in the Nature Research Reporting Summary linked to this article.

## Supplementary information

Supplementary Information

Peer Review File

Reporting Summary

Description of Additional Supplementary Files

Supplementary Data 1

Supplementary Data 2

Supplementary Data 3

## Data Availability

The data that support this study are available from the corresponding authors upon reasonable request. The source data underlying Fig. 2–5 and Supplementary Figs. 1–10 and 12–23 are provided as a source data file and in Supplementary Data 2 and 3.

## Source Data

Source Data
