# Supplementary material for: Quantifying CDK inhibitor selectivity in live cells
Source: Nat Commun. 2020 Jun 2;11:2743. doi: 10.1038/s41467-020-16559-0 (PMC7265341; doi:10.1038/s41467-020-16559-0)
Supplement: Supplementary file 1 — Supplementary Information [file 41467_2020_16559_MOESM1_ESM.pdf]

Quantifying CDK inhibitor selectivity in live cells

Wells et al.

Table of Contents

Supplementary Methods 2
Supplementary Figures 13
Supplementary Tables 52
Supplementary References 54

Supplementary Methods

Linearized Cheng-Prusoff Analysis

To evaluate the impact of energy transfer probe concentration to compound potency measurements, potency data was collected at increasing concentrations of energy transfer probe. Potency values were replotted as a function of probe concentration and fitted to the linearized Cheng-Prusoff equation (Supplementary Equation 1).

$$IC_{50} = K_{i,app}*(1 + [probe]/K_{d,app}) \quad (1)$$

where $K_{i,app}$ = the apparent K_i value of the compound under live-cell conditions, and $K_{d,app}$ = the apparent K_d of the probe under the live-cell conditions.[1]

NanoBiT Complementation Assays to selectively measure target engagement at CDK2/Cyclin E1 complexes. SmBiT and LgBiT reporters[2] were encoded in pFN220 and pFN217 (CMV) vector backbones. SmBiT was tagged onto the c-terminus of CDK2 (NP_001789) and encoded the following linker between the CDK2 and SmBiT coding region: VSQGSSGGGGSGGGGSSG. LgBiT-cyclin E1 (NP_001229) and LgBiT-p53 (NP_000537.3) negative control encoded the following linker between the LgBiT and desired coding region: GSSGGGGSGGGGSSGAIA. Target engagement analysis was then performed under transfection and assay conditions identical to that used for CDK2-NanoLuc/Cyclin E1. Binary complex signal from the

CDK2/Cyclin E1 complex was measured in the donor NanoBRET channel (using the standard 450 nm bandpass filter on the Glomax Discover plate reader). Ternary complex was measured in the presence of 0.5 µM probe 5. Ternary complex signal from the CDK2/Cyclin E1/probe 5 complex was measured in the acceptor NanoBRET channel (using the standard 600 nm longpass filter on the Glomax Discover plate reader). Total LgBiT expression level was determined in the presence of a saturating concentration (100 nM) of high affinity HiBiT peptide (Peptide 2.0), 1X NanoBRET Target Engagement substrate, and 50 mg/mL digitonin (as a permeabilization agent).

Real-time analysis of RGB286638 engagement to CDK6 and CDK7 in live cells. RGB286638 binding to CDK6 and CDK7 was measured in real time by displacement of energy transfer probe 2 or probe 5, respectively, at probe concentrations identical to those described for steady state analysis of target engagement. Cells were pre-equilibrated with energy transfer probe for 40 min prior to addition of various concentrations of RGB286638, after which the BRET was measured kinetically at 3 minute intervals at 25 °C (temperature reduced to avoid evaporation of cell culture medium). To quantify apparent rates of equilibration for RGB286638 to CDK6 vs CDK7, engagement data were plotted as IC_{50} vs time. Apparent association rate was also plotted as k_{obs} vs [RGB286638], where k_{obs} was determined using the one phase association equation (Supplementary Equation 2) in graphpad prism.

$$Y = Y_0 + (\text{Plateau} - Y_0)*(1 - e^{-K*X}) \qquad (2)$$

where Y = mBRET ratio, X = time, and K = the observed rate constant (k_{obs}). Both analyses support relatively slow engagement of RGB286638 to CDK6 compared to CDK7.

Generation of endogenously edited CDK-HiBiT cell lines and target engagement analysis at endogenous protein levels. HEK293 cells were edited to express HiBiT fused to the N-terminus of CDK9 or the C-terminus of CDK16, as described previously[3]. For CDK9, the sequence of the guide RNA was CGGCGGCGGCGCGTTGGAGG, and the sequence of the donor DNA was

gagcggcggcagcagcgactgggggcggcggcgcgttggaggccgccatgGTCTCCGTGAGCGGCTGGCGGCTGTTCAAGAAGATTAGCgcaaagcagtacgactcggtggagtgccctttttgtgatgaagtttc (integrated nucleotides are capitalized and PAM site mutation that blocks re-cutting is underlined). For CDK16, the guide RNA sequence was GAGTTCTAAGCCACAGACCG, and the donor DNA sequence was tgctttcccccacaggcaggccagctttccgcgtggtggacaccgagttcGTCTCCGTGAGCGGCTGGCGGCTGTTCAAGAAGATTAGCtaagccacagaccgaggccccagcaggcagcggctggagggatgccacac (integrated nucleotides are capitalized). Briefly, ribonucleoprotein (RNP) complexes were assembled by combining 100 pmol (Alt-R S.p. Cas9 Nuclease V3, Integrated DNA Technologies, IDT) and 120 pmol of guide RNA (Alt-R CRISPR-Cas9 crRNA:tracrRNA duplex, IDT) for 10 min at ambient temperature. 2×10^5 HEK293 cells were resuspended in 20 µl of 4D Nucleofector Solution SF (Lonza), and RNP complexes along with 100 pmol single stranded DNA template (Ultramer DNA Oligo, IDT) were electroporated into the cells using the Nucleofector 4D System (Lonza) and program CM-130. Following a 5 min incubation at ambient temperature, cells were transferred to a six-well plate containing 2 ml of growth medium. 7 days post-editing, live cell singlets were isolated by sorting into 96-well plates using the FACSMelody cell sorter (BD Biosciences). Clones were expanded, copy plated, and screened for luminescence using the Nano-Glo HiBiT Lytic Detection System (Promega) per the manufacturer's instructions. Luminescence positive clones were sequence validated to identify those harboring HiBiT integration in at least one allele and to confirm the absence of mutations to the coding region of the genes, as described previously[4]. To generate luminescent CDK-NanoBiT complexes in cells, cells were transduced with LgBiT/BacMam virus. 2×10^4 cells were plated in solid white 96-well tissue culture plates in 100 µl growth medium containing 5% (v/v) BacMam CMV-LgBiT reagent (Kempbio, Inc., viral titer approximately 2×10^8 PFU/ml) and grown for 24 h. Energy transfer was measured under conditions described earlier. 0.25 µM probe 5 was used to measure target engagement for CDK9 and CDK16.

Target Engagement Analysis in MCF7 cells. On the day prior to transfection, 0.1 mL / well of MCF7 cells (ATCC) were seeded into 96-well plates (Corning 3917) in DMEM + 10% FBS at densities of 25,000 cells per well. After 20 hours of incubation, cells were transfected with NLuc-CDK6 or NLuc-CDK4 plasmids in combination with Cyclin D1 expression plasmid using conditions similar to those described for HEK293 cells, but using Viafect transfection reagent and a 6:1 Viafect:DNA ratio (volume:mass). 20 hours post transfection, medium was replaced with Opti-MEM medium. The target engagement assay conditions and probe 2 concentrations were identical to those used for HEK293 cells (0.063µM).

Endogenous phospho-Rb analysis in MCF7 cells.

The level of phospho-Rb (Ser 807/811) was measured with Lumit Immunoassay Cellular System as follows; 50,000 MCF-7 cells were seeded in 160 µl complete growth medium (DMEM + 10% FBS) per well into a 96 well plate and incubated overnight. The next day, the medium was replaced with phenol-red free DMEM medium without serum and the cells were incubated overnight. Then, the cells were treated with various concentration of the compounds in 200 µl volume in the presence of 25 ng/ml hEGF (Promega, G5021) for 24 hours. After treatment, the samples in the plates were analyzed with Lumit Immunoassay Cellular System (Promega, W1331) to detect phospho-Rb following the manufacturer's recommendations and as described[5]. The following antibodies were purchased from Cell Signaling Technology Inc. and were used at 150 ng/ml: Rabbit anti-phospho-Rb (#8516) and Mouse anti-Rb (#9309). Briefly, the medium was replaced with 40 µl of 1X immunoassay buffer and 10 µl of lysis buffer was added. The plates were mixed vigorously for 20 minutes. Then, 50 µl of an antibody mix in 1X immunoassay buffer containing two primary antibodies against the phospho-Rb protein and the two Lumit secondary antibodies (Lumit Anti-Mouse Ab-SmBiT and Anti-rabbit Ab-LgBiT) was added to the lysates. The plates were incubated at 23°C for 90 minutes, followed by the addition of 25 µl of Lumit detection reagent. Luminescence was measured after 2 minutes using a plate-reading luminometer.

Data represents % Rb phosphorylation normalized to the control wells that contained vehicle treated cells (100%) and the 0% phosphorylation wells where all the detection reagents were added except for primary antibodies.

General Chemical Synthesis Information

All solvents were purchased from Sigma or Fisher Scientific and used without purification. AT7519 was purchased from MedKoo Biosciences, Morrisville NC. NanoBRET® 590 SE was obtained from Promega Corp. Madison, WI. ^{1}H-NMR spectra were recorded on a Bruker Avance 400 MHz spectrometer or a Bruker Ascend 400 MHz spectrometer. Chemical shifts (δ) are quoted in parts per million (ppm) and referenced to the residual solvent peak. Multiplicities are denoted as s-singlet, d-doublet, t-triplet, q-quartet and quin-quintet and derivatives thereof (br denotes a broad resonance peak). Coupling constants are given in Hz and round to the nearest 0.1 Hz. Mass spectra were recorded on a Waters SQ Detector 2 (LC-MS) and purity (≥95 %) determined by reverse-phase high pressure liquid chromatography (RP-HPLC) using a Kinetex 5 µm EVO C18 100 Å LC Column 30 × 2.1 mm column or a Phenomenex Synergi 2.5 µm Max-RP 100 Å LC column. High resolution mass spectrum (HRMS) were recorded on a SCIEX Triple TOF 5600 spectrometer. Compounds were purified on a Waters LC Prep 150 using a Waters XBridge Prep C18 OBD 30x250mm column. Standard Method 1: Initial - 90% aqueous (0.1% TFA in H_2O), 10% acetonitrile to 0% aqueous, 100% acetonitrile, 30 min linear gradient.

Synthetic Methods

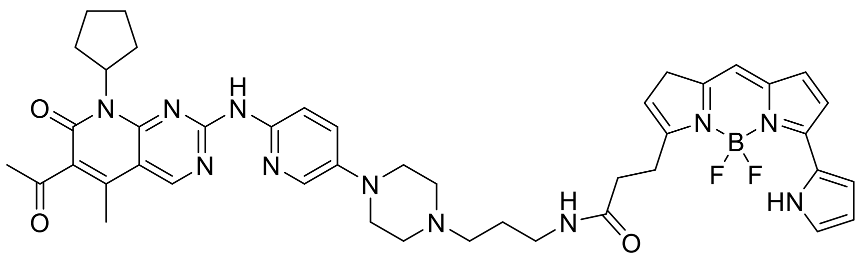

***N*-(3-(4-(6-((6-acetyl-8-cyclopentyl-5-methyl-7-oxo-7,8-dihydropyrido[2,3-*d*]pyrimidin-2-yl)amino)pyridin-3-yl)piperazin-1-yl)propyl)-3-(5,5-difluoro-7-(1*H*-pyrrol-2-yl)-5*H*-5λ^{4},6λ^{4}-dipyrrolo[1,2-*c*:2',1'-*f*][1,3,2]diazaborinin-3-yl)propenamide, (Probe 2).**

To a solution of 6-acetyl-2-((5-(4-(3-aminopropyl)piperazin-1-yl)pyridin-2-yl)amino)-8-cyclopentyl-5-methylpyrido[2,3-d]pyrimidin-7(8H)-one (PMID: 26390342) (6.56 mg, 13 µmol) in DMF (500 µL), DIPEA (150 µL, 859 µmol) was added and the reaction mixture stirred for 10 min. After addition of NanoBRET 590 SE (5.2 mg, 12 µmol), the reaction mixture was stirred for another 2 hr at room temperature. The solvent was removed and the product purified by preparative TLC using 4% ammonia/methanol in dichloromethane as eluent. Extraction from silica using 20% methanol in dichloromethane followed by solvent removal afforded the final purified product in moderate yield as a purple solid (3.5 mg, 4.3 µmol, 36% yield). **^{1}H NMR** (400 MHz, DMSO-d6) δ 11.43 (s, 1H), 10.08 (s, 1H), 8.94 (s, 1H), 8.04 (d, J = 2.9 Hz, 1H), 7.96 (s, 1H), 7.84 (d, J = 9.0 Hz, 1H), 7.44 (s, 1H), 7.37 (d, J = 3.8 Hz, 1H), 7.33 (d, J = 4.5 Hz, 1H), 7.26 (td, J = 2.7, 1.3 Hz, 1H), 7.17 (d, J = 4.6 Hz, 1H), 7.01 (d, J = 4.0 Hz, 1H), 6.33 (dd, J = 4.1, 2.1 Hz, 2H), 5.86 – 5.73 (m, 2H), 3.14 (t, J = 7.9 Hz, 8H), 2.41 (s, 3H), 2.30 (s, 3H), 2.22 (d, J = 9.6 Hz, 4H), 1.91 – 1.69 (m, 6H), 1.63 – 1.50 (m, 4H), 1.22 (s, 2H), 0.89 – 0.75 (m, 2H). **MS** (**ESI, *m/z)*** calcd. for $C_{43}H_{50}BF_5N_{11}O_3$ [M+H]$^{+}$: 816.8, found 816.5.

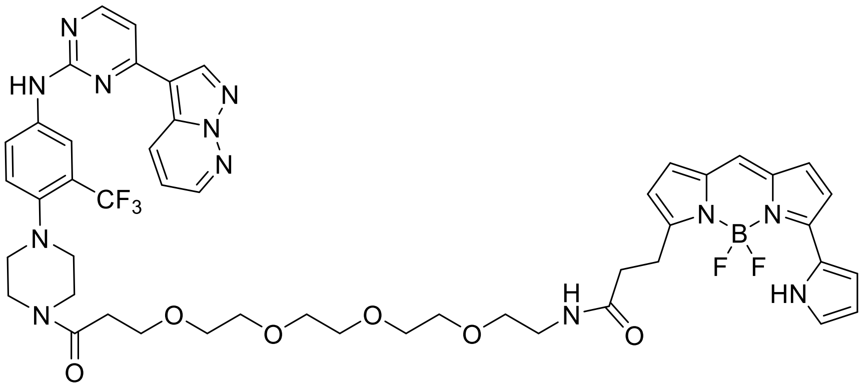

3-(5,5-difluoro-7-(1*H*-pyrrol-2-yl)-5*H*-5λ[4],6λ[4]-dipyrrolo[1,2-*c*:2',1'-*f*][1,3,2]diazaborin in-3-yl)-*N*-(15-oxo-15-(4-(4-((4-(pyrazolo[1,5-*b*]pyridazin-3-yl)pyrimidin-2-yl)amino)-2-(trifluoromethyl)phenyl)piperazin-1-yl)-3,6,9,12-tetraoxapentadecyl)propenamide, (Probe 3)

A 100 mL round bottom flask was charged with 1-amino-15-(4-(4-((4-(pyrazolo[1,5-*b*]pyridazin-3-yl)pyrimidin-2-yl)amino)-2-(trifluoromethyl)phenyl)piperazin-1-yl)-3,6,9,12-tetraoxapentadecan-15-one trifluoroacetic acid salt (34.0 mg, 0.016 mmol) and was taken up in DMF (5.0 mL). The mixture was treated with *N*-*N*-diisopropylethylamine (36.9 uL, 0.212 mmol) and was stirred for 10 min. NanoBRET 590 SE (18.1 mg, 0.042 mmol) was added and the mixture was stirred in the dark for 3 hrs. The reaction mixture was diluted to 8 mL with 1:1:0.01 water, ACN, TFA and was subjected to reverse-phase preparative HPLC purification using Standard Method 1. Product containing fractions were pooled and concentrated under reduced pressure to give a purple film that was treated with 10 mL ACN and concentrated to dryness three times. The resulting film was dried overnight under high vacuum to give the product (28.7 mg, 67.7%) as a purple solid. **^{1}H NMR** (400 MHz, CD_2Cl_2) δ 10.50 – 10.22 (m, 1H), 8.89 – 8.74 (m, 1H), 8.50 (s, 1H), 8.44 – 8.35 (m, 2H), 8.12 (d, *J* = 3.3 Hz, 1H), 7.33 (d, *J* = 9.1 Hz, 1H), 7.22 – 7.09 (m, 3H), 7.06 (d, *J* = 5.1 Hz, 1H), 7.02 (s, 1H), 6.86 (d, *J* = 5.1 Hz, 2H), 6.33 (dd, *J* = 21.7, 4.1 Hz, 2H),

3.76 (t, *J* = 6.9 Hz, 3H), 3.58 (d, *J* = 4.2 Hz, 17H), 3.45 – 3.40 (m, 2H), 3.28 (t, *J* = 8.2 Hz, 2H), 2.85 (p, *J* = 6.3 Hz, 4H), 2.62 (td, *J* = 8.1, 7.6, 3.5 Hz, 4H), 2.08 – 1.75 (m, 2H). **^{13}C NMR** (101 MHz, CD_2Cl_2) δ 171.93, 169.77, 160.65, 160.43, 158.45, 156.56, 150.77, 146.89, 143.83, 139.99, 137.79, 137.70, 134.02, 133.61, 132.12, 129.88, 128.65, 128.37, 128.09, 127.80, 127.06, 126.27, 125.93, 125.56, 124.57, 124.12, 123.99, 123.21, 120.68, 119.02, 118.96, 118.91, 118.85, 118.71, 118.15, 117.12, 111.91, 110.88, 109.02, 71.03, 71.01, 70.99, 70.95, 70.78, 70.37, 67.87, 46.68, 42.53, 39.85, 35.72, 34.03, 25.06, 25.04, 25.02. **MS** (**ESI, *m/z)*** calcd. for $C_{48}H_{51}BF_5N_{12}O_6$ $[M-H]^-$: 997.4068, found: 997.4059.

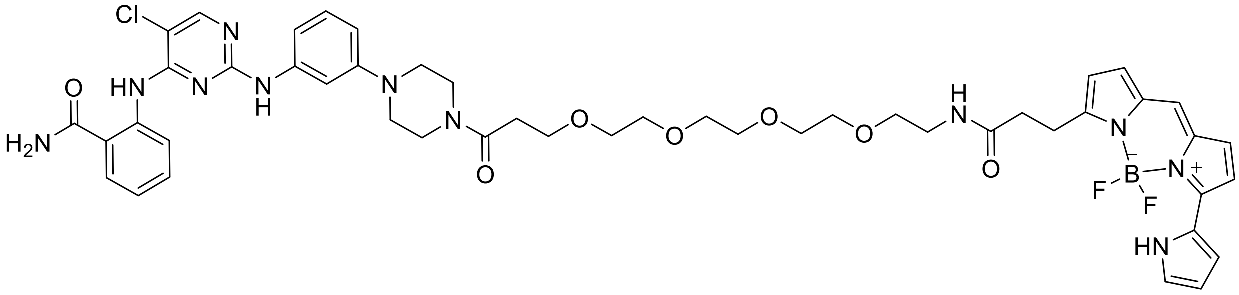

2-((5-chloro-2-((3-(4-(1-(5,5-difluoro-7-(1*H*-pyrrol-2-yl)-5H-5λ^4,6λ^4-dipyrrolo[1,2-*c*:2',1'-*f*][1,3,2]diazaborinin-3-yl)-3-oxo-7,10,13,16-tetraoxa-4-azanonadecan-19-oyl)piperazin-1-yl)phenyl)amino)pyrimidin-4-yl)amino)benzamide, (Probe 4)

A 100 mL round bottom flask was charged with 2-((5-chloro-2-((3-(4-(1-(chloro-λ^4-azaneyl)-3,6,9,12-tetraoxapentadecan-15-oyl)piperazin-1-yl)phenyl)amino)pyrimidin-4-yl)amino)benzamide (30.0 mg, 0.042 mmol) and was taken up in DMF (3.5 mL). The mixture was treated with *N-N*-diisopropylethylamine (8.6 uL, 0.049 mmol) and was stirred for 10 min. NanoBRET 590 SE (15.0 mg, 0.035 mmol) was added and the mixture was stirred in the dark for 40 min. The reaction mixture was diluted into EtOAc (70 mL) and was washed three times with 0.25 M sodium citrate solution (35 mL).

The organic layer was dried over Na_2SO_4, absorbed on celite, concentrated to dryness and was subjected to silica gel flash chromatography using a 10% MeOH/DCM gradient. The purified product was concentrated to give the product (34.0 mg, 98.1%) as a purple solid. **^{1}H NMR** (400 MHz, CD_2Cl_2) δ 11.30 (s, 1H), 10.36 (s, 1H), 8.53 (d, J = 8.4 Hz, 1H), 8.07 (s, 1H), 7.66 (d, J = 7.8 Hz, 1H), 7.43 – 7.31 (m, 3H), 7.21 – 7.13 (m, 2H), 7.06 – 6.96 (m, 4H), 6.83 (m, 4H), 6.59 (dd, J = 8.1, 2.3 Hz, 1H), 6.36 (m, 1H), 6.26 (d, J = 4.0 Hz, 1H), 3.72 (t, J = 6.7 Hz, 2H), 3.58 (m, 16H), 3.43 (q, J = 5.1 Hz, 2H), 3.37 (t, J = 5.1 Hz, 2H), 3.26 (t, J = 7.8 Hz, 2H), 2.93 (t, J = 5.3 Hz, 2H), 2.83 (t, J = 5.0 Hz, 2H), 2.61 (t, J = 7.8 Hz, 2H), 2.50 (t, J = 6.7 Hz, 2H). **^{13}C NMR** (101 MHz, CD_2Cl_2) δ 171.56, 171.49, 168.87, 157.66, 156.15, 155.50, 154.59, 151.78, 150.15, 140.50, 140.19, 137.19, 133.46, 132.39, 131.53, 129.08, 128.14, 126.52, 125.70, 123.58, 123.44, 121.99, 121.85, 120.13, 120.07, 117.54, 116.46, 111.45, 111.36, 111.32, 107.63, 106.57, 70.42, 70.39, 70.36, 70.27, 70.21, 69.92, 67.33, 49.29, 49.21, 45.30, 41.32, 39.36, 35.00, 33.42, 24.46. **HRMS** (**ESI, *m/z)*** calcd. for $C_{48}H_{55}BClF_2N_{11}O_7$ $[M+H]^+$: 982.4114, found: 982.4105

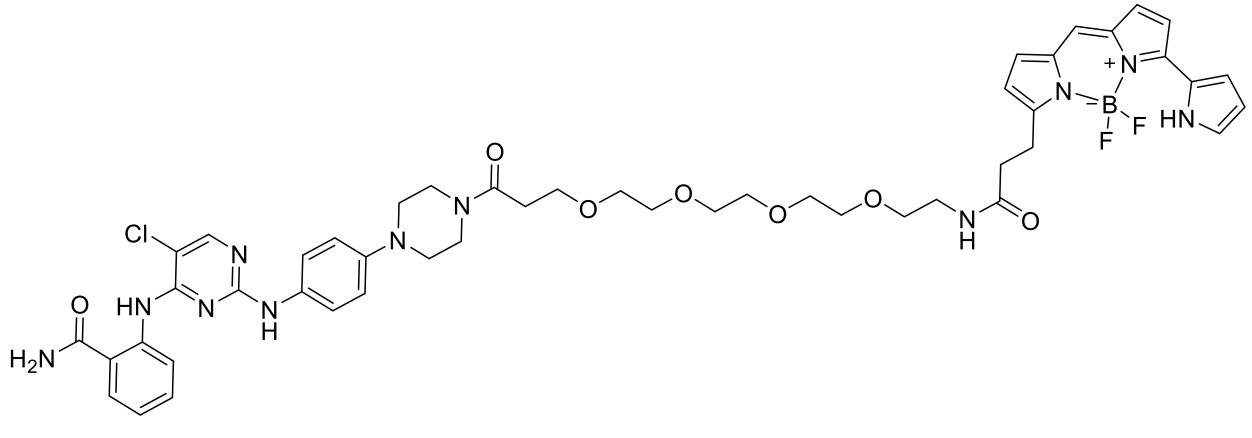

2-((5-chloro-2-((4-(4-(1-(5,5-difluoro-7-(1*H*-pyrrol-2-yl)-5H-5λ^4,6λ^4-dipyrrolo[1,2-*c*:2',1'-*f*][1,3,2]diazaborinin-3-yl)-3-oxo-7,10,13,16-tetraoxa-4-azanonadecan-19-oyl)piperazin-1-yl)phenyl)amino)pyrimidin-4-yl)amino)benzamide, (Probe 5)

A 100 mL round bottom flask was charged with 2-((2-((4-(4-(1-amino-3,6,9,12-tetraoxapentadecan-15-oyl)piperazin-1-yl)phenyl)amino)-5-chloropyrimidin-4-yl)amino)benzamide trifluoroacetic acid salt (34.0 mg, 0.0433 mmol) and was taken up in DMF (3.5 mL). The mixture was treated with *N*-*N*-diisopropylethylamine (8.7 uL, 0.050 mmol) and was stirred for 10 min. NanoBRET 590 SE (14.2 mg, 0.033 mmol) was added and the mixture was stirred in the dark for 80 min. The reaction mixture was concentrated under reduced pressure, dissolved in DMF containing 1% TFA (1.5 mL) and was subjected to reverse-phase preparative HPLC purification using Standard Method 1. Product containing fractions were pooled and concentrated under reduced pressure to give a purple film that was treated with 10 mL ACN and concentrated to dryness three times. The resulting residue was dried overnight under high vacuum to give the product (24.0 mg, 73.4%) as a purple solid. **^{1}H NMR** (400 MHz, CD_2Cl_2) δ 11.31 (s, 1H), 10.38 (s, 1H), 8.70 (d, J = 8.5 Hz, 1H), 8.03 (s, 1H), 7.62 (d, J = 7.8 Hz, 1H), 7.49 – 7.35 (m, 3H), 7.17 (m, 2H), 7.11 – 6.97 (m, 4H), 6.90 – 6.81 (m, 4H), 6.47 (m, 1H), 6.37 (m, 1H), 6.29 (d, J = 4.0 Hz, 1H), 3.72 (m, 4H), 3.62 – 3.48 (m, 16H), 3.41 (q, J = 5.2 Hz, 2H), 3.27 (t, J = 7.8 Hz, 2H), 3.06 (q, J = 5.7, 5.3 Hz, 4H), 2.60 (m, 4H). **^{13}C NMR** (101 MHz, CD_2Cl_2) δ 171.46, 171.26, 169.18, 158.15, 155.95, 155.73, 154.36, 150.24, 147.27, 140.40, 137.25, 133.47, 132.47, 132.28, 131.60, 127.80, 126.50, 125.79, 123.57, 123.46, 122.07, 122.03, 121.87, 120.15, 119.65, 117.63, 117.03, 116.50, 111.36, 106.13, 70.44, 70.38, 70.23, 69.82, 67.29, 50.29, 49.85, 45.54, 41.39, 39.32, 35.10, 33.43, 24.47. **HRMS** (**ESI, *m/z)*** calcd. for $C_{48}H_{55}BClF_2N_{11}O_7$ $[M+H]^+$: 982.4114, found: 982.4114

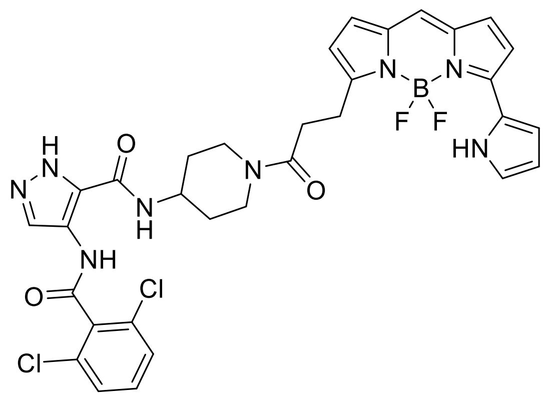

4-(2,6-dichlorobenzamido)-*N*-(1-(3-(5,5-difluoro-7-(1*H*-pyrrol-2-yl)-5H-5λ^4,6λ^4-dipyrrolo[1,2-*c*:2',1'-*f*][1,3,2]diazaborinin-3-yl)propanoyl)piperidin-4-yl)-1*H*-pyrazole-5-carboxamide, (Probe 1)

AT7519 (10.0 mg, 0.029 mmol) was charged into an amber vial and was taken up in DMF (2.0 mL). The mixture was treated with *N*-*N*-diisopropylethylamine (22.8 uL, 0.131 mmol) and was stirred for 10 min. NanoBRET 590 SE (12.3 mg, 0.0288 mmol) was added and the mixture was stirred in the dark for 2 hrs. The reaction mixture was diluted to 8 mL with 1:1:0.01 water, ACN, TFA and was subjected to reverse-phase preparative HPLC purification using Standard Method 1. Product containing fractions were pooled and concentrated under reduced pressure to give a purple film that was treated with 10 mL ACN and concentrated to dryness three times. The resulting film was dried overnight under high vacuum to give the product (14.8 mg, 81.6%) as a purple solid. **^{1}H NMR** (400 MHz, DMSO-d_6) δ 13.41 (s, 1H), 11.41 (s, 2H), 10.17 (s, 2H), 8.42 (d, *J* = 8.1 Hz, 2H), 8.33 (d, *J* = 14.0 Hz, 2H), 7.58 (d, *J* = 7.2 Hz, 4H), 7.52 (dd, *J* = 9.3, 6.6 Hz, 2H), 7.44 (s, 2H), 7.39 – 7.31 (m, 4H), 7.26 (d, *J* = 3.2 Hz, 2H), 7.16 (d, *J* = 4.5 Hz, 2H), 7.02 (d, *J* = 3.9 Hz, 2H), 6.42 (d, *J* = 4.0 Hz, 2H), 6.33 (dt, *J* = 4.6, 2.4 Hz, 2H), 4.41 (d, *J* = 13.0 Hz, 2H), 3.97 (qt, *J* = 13.3, 10.2, 4.4 Hz, 4H), 3.13 (t, *J* = 8.0 Hz, 4H), 3.05 (t, *J* = 12.8 Hz, 2H), 2.74 (td, *J* = 7.3, 2.6 Hz, 4H), 2.63 (t, *J* = 13.0 Hz, 2H), 1.82 – 1.71 (m, 4H), 1.52 (ddt, *J* = 34.5, 14.4, 6.6 Hz, 4H). **^{13}C NMR** (101 MHz, DMSO) δ 169.22, 162.61, 160.33, 156.14, 150.12, 136.87, 135.37, 133.05, 132.34, 131.89, 131.26, 128.43, 126.83, 126.04, 124.41, 122.90, 121.49, 119.26, 117.33, 116.76, 111.48, 79.17, 45.97, 43.91, 31.73, 31.50, 30.91, 24.01. **HRMS** (**ESI, *m/z)*** calcd. for $C_{32}H_{28}BCl_2F_2N_8O_3$ [M-H]$^-$: 681.1723, found: 697.1722.

Supplementary Figures

Supplementary Figure 1

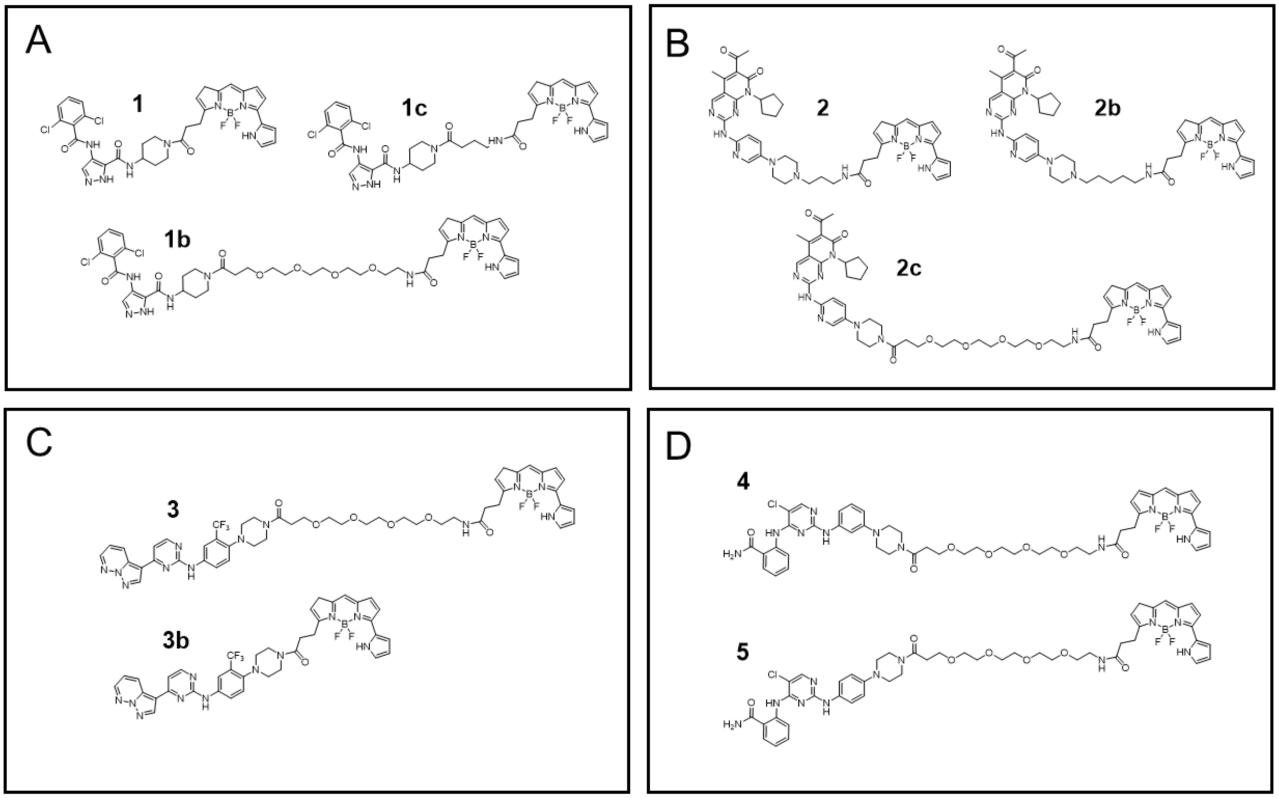

E

CDK	Relative BRET Signal			Relative BRET Signal			Relative BRET Signal		Relative BRET Signal	
	Probe 1	Probe 1b	Probe 1c	Probe 2	Probe 2b	Probe 2c	Probe 3	Probe 3b	Probe 5	Probe 4
CDK1 + Cyclin E1	2.04	1.68	1.52	1.08	1.11	1	1.23	1.04	2.46	3.1
CDK2 + Cyclin E1	5.37	4.44	6.63	1.04	1.02	1	2.45	1.29	11.56	8.83
CDK3 + Cyclin E1	5.25	3.16	3.31	1.08	1.05	0.97	1.55	1.11	5.63	9.16
CDK4 + Cyclin D1	4.34	1.36	3.02	5.53	1.46	3.57	1.43	1.07	4.3	1.37
CDK5 + CDK5R1	4.6	2.4	4.36	1.09	0.97	1.02	1.8	1.1	9.46	5.55
CDK6 + Cyclin D1	3.05	1.18	2.06	4.09	1.86	3.53	1.18	1	3.14	1.46
CDK7	4.36	1.13	2.01	1.03	1.11	1.03	1.76	1.1	3.21	1.33
CDK8 + Cyclin C	1.22	1.04	1.06	1.04	1.07	1.03	5.08	3.01	1.07	1.08
CDK9 + Cyclin K	8.5	2.76	8.06	1.63	1.07	1.17	8.18	3.64	3.74	3.53
CDK10 + Cyclin L2	2.48	1.05	1.33	1.03	1.18	1.04	1.15	1	2.67	1.43
CDK11A + Cyclin L2	5.38	1.23	2.09	1.05	1.14	0.99	1.14	0.96	1.14	1.06
CDK11B + Cyclin L2	5.85	1.16	1.91	1.06	1.26	1.01	1.05	0.97	1.13	1.03
CDK12 + Cyclin K	1.25	1.05	1.13	1.03	1.08	1.04	1.21	1.08	1.44	1.39
CDK13 + Cyclin K	1.29	1	1.11	1.01	1.07	0.97	1.17	1.02	1.29	1.29
CDK14 + Cyclin Y	5.96	2.07	3.33	1.25	1	1.02	2.18	1.21	3	1.26
CDK15 + Cyclin Y	5.69	2.82	4.76	1.09	1.14	1.08	2.51	1.09	3.31	1.74
CDK16 + Cyclin Y	5.63	2.37	2.27	1.19	0.97	1.07	6.4	1.42	4.83	2.21
CDK17 + Cyclin Y	6.27	2.13	2.51	1.32	1	1.05	8.31	1.58	6.44	2.21
CDK18 + Cyclin Y	8.23	1.98	2.72	1.23	1.04	1.04	6.22	1.36	8.15	3.25
CDK19 + Cyclin C	1.13	1.03	1.06	1.05	1.03	1	2.37	1.67	1.06	1.02
CDK20 + Cyclin H	1.2	1.02	1.03	1.05	1.39	0.99	0.91	0.79	5.61	2.93

Supplementary Figure 1. Linker evaluation for energy transfer probes. For each base compound, linker length, properties, or positioning was evaluated by screening for specific BRET signals across the panel of CDKs. Probes were added to cells expressing CDK/NanoLuc fusions at a concentration of 0.5µM in the presence of 20µM of the unlabeled parent compound to demonstrate specificity. For each probe/CDK pair, specific BRET is reported as a relative BRET signal by normalizing the raw BRET value for the tracer only samples (n = 2) to the raw BRET value in the presence of 20µM of the unlabeled parent compound (n = 2). Probe structures are depicted in panels A, B, C, and D, with corresponding relative BRET signals tabulated in panel E. Relative BRET signals in panel E represent values measured in a single (n = 1) biological experiment. Source data are provided as a Source Data File.

Supplementary Figure 2

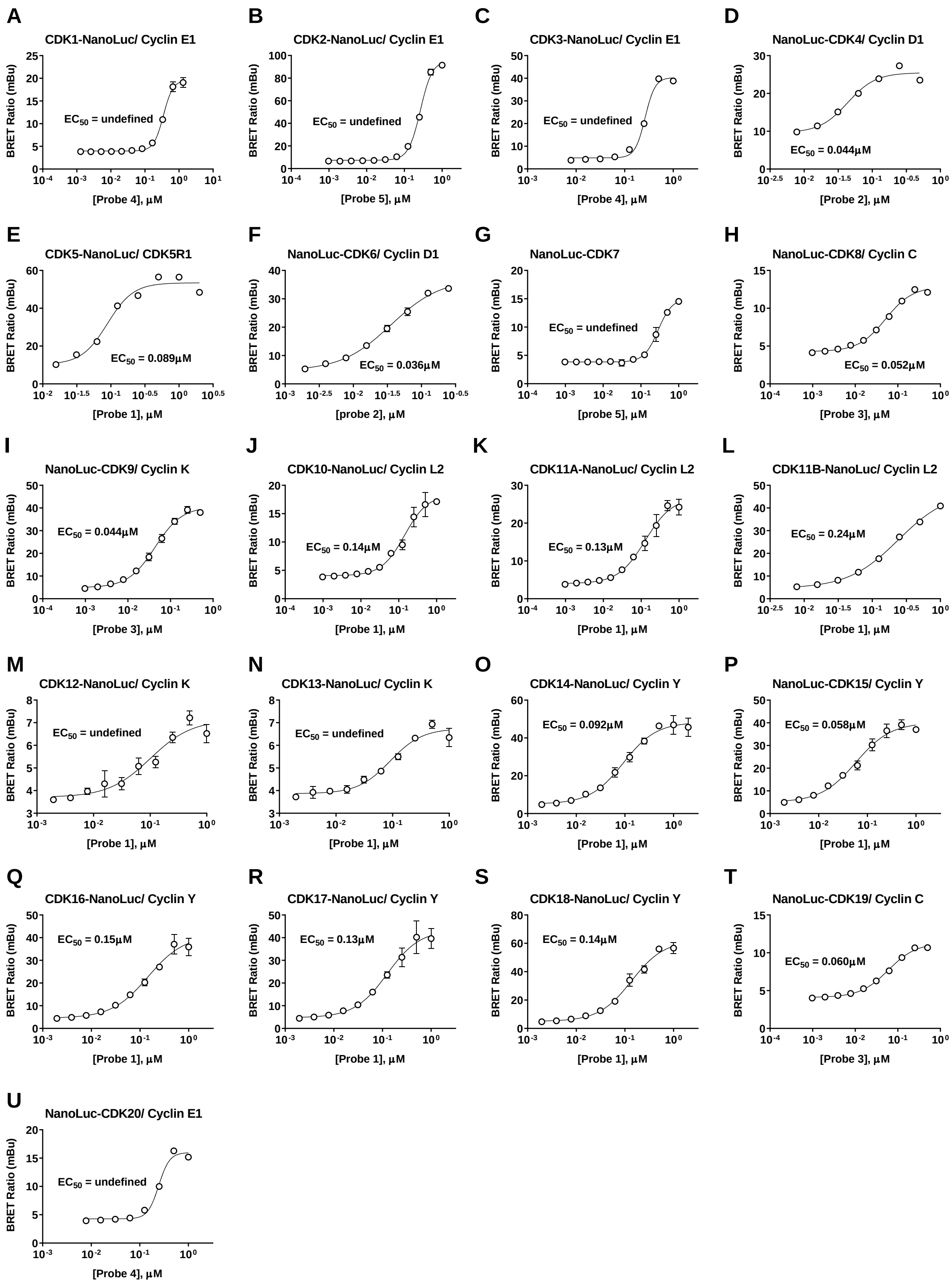

Supplementary Figure 2. Energy probe characterization for each CDK assay. Energy probes were characterized as described in the methods section. Briefly, energy probes were titrated onto HEK-293 cells expressing CDK/NanoLuc fusions up to a maximum concentration of 1µM (the functional performance limit of most of these energy transfer probes, see Supplementary Figure 5). The BRET ratio was measured as a function of energy probe concentration, and the EC_{50} value of the tracer was interpolated using Equation 1 (see methods section). For some CDK assays, the BRET was not saturable before reaching the energy probe solubility threshold (see Supplementary Figure 5), and the EC_{50} value is reported as undefined. In these cases, the absence of impact of tracer concentration on compound IC_{50} (Supplementary Figure 3) and a lack of a strong Cheng-Prusoff relationship (Supplementary Figure 4) provide support for the tracer concentrations employed in this study. Dose response curves in each plot were collected in a single biological experiment (n = 1), where individual data points represent either singlicate values (n = 1; panels C, D, E, L, and U), the mean ± S.D. of 3 technical replicates (n = 3; panels M and N), or the mean ± S.D. of 4 technical replicates (n = 4; panels A, B, F, G, H, I, J, K, O, P, Q, R, S, and T). Source data are provided as a Source Data File.

Supplementary Figure 3

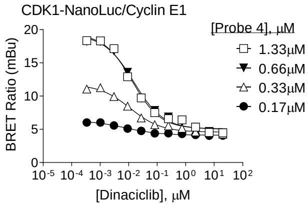

[Tracer], µM	1.33	0.66	0.33	0.17
IC_{50} (µM)	0.013	0.015	0.011	0.0076

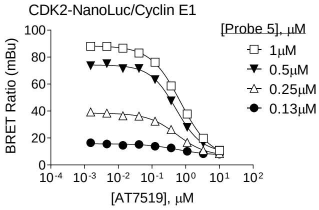

[Probe 5], µM	1	0.5	0.25	0.13
IC_{50} (µM)	0.7	0.51	0.55	0.58

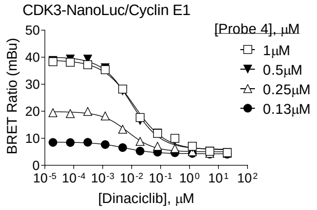

[Probe 4], µM	1	0.5	0.25	0.13
IC_{50} (µM)	0.012	0.0096	0.0073	0.0057

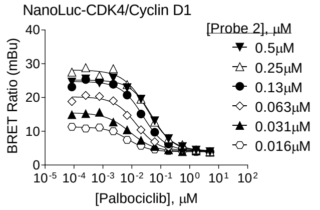

[Probe 2], µM	0.5	0.25	0.13	0.063	0.031	0.016
IC_{50} (µM)	0.049	0.035	0.024	0.014	0.0087	0.0073

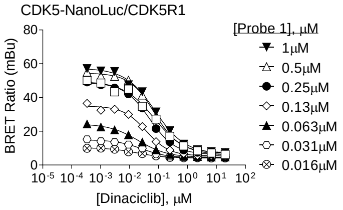

[Probe 1], µM	1	0.5	0.25	0.13	0.063	0.031	0.016
IC_{50} (µM)	0.077	0.072	0.048	0.037	0.020	0.026	0.018

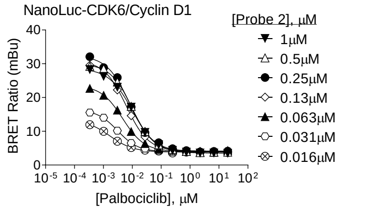

[Probe 2], µM	1	0.5	0.25	0.13	0.063	0.031	0.016
IC_{50} (µM)	0.010	0.0098	0.0081	0.0062	0.0046	0.0031	0.0017

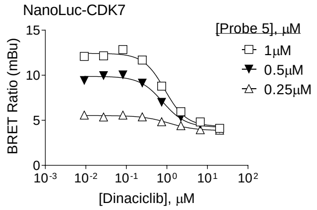

[Probe 5], µM	1	0.5	0.25
IC_{50} (µM)	0.89	0.79	1.1

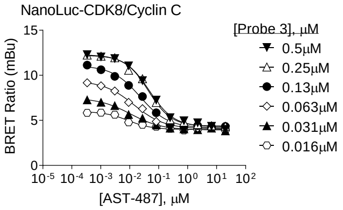

[Probe 3], µM	0.5	0.25	0.13	0.063	0.031	0.016
IC_{50} (µM)	0.048	0.044	0.023	0.014	0.011	0.0088

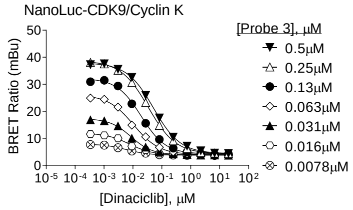

[Probe 3], µM	0.5	0.25	0.13	0.063	0.031	0.016	0.0078
IC_{50} (µM)	0.052	0.034	0.019	0.010	0.0086	0.0076	0.0059

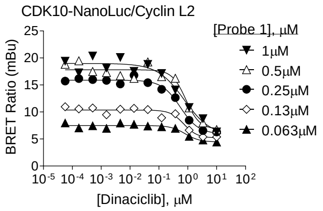

[Probe 1], µM	1	0.5	0.25	0.13	0.063
IC_{50} (µM)	0.74	0.85	0.55	0.72	0.81

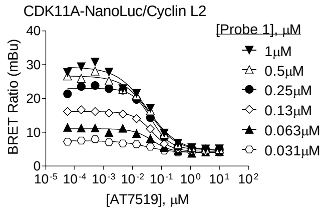

[Probe 1], µM	1	0.5	0.25	0.13	0.063	0.031
IC_{50} (µM)	0.033	0.039	0.044	0.046	0.049	0.032

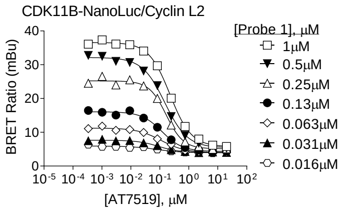

[Probe 1], µM	1	0.5	0.25	0.13	0.063	0.031	0.016
IC_{50} (µM)	0.22	0.15	0.17	0.12	0.11	0.090	0.077

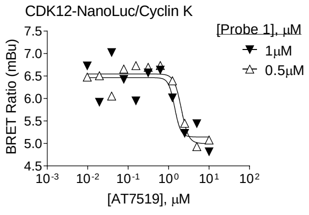

[Probe 1], µM	1	0.5
IC_{50} (µM)	1.4	2.1

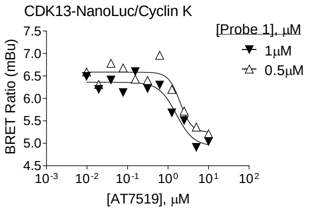

[Probe 1], µM	1	0.5
IC_{50} (µM)	1.6	2

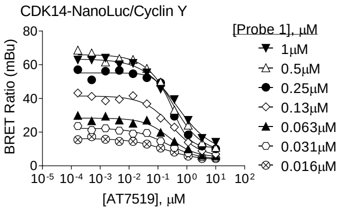

[Probe 1], µM	1	0.5	0.25	0.13	0.063	0.031	0.016
IC_{50} (µM)	0.44	0.32	0.34	0.31	0.25	0.21	0.17

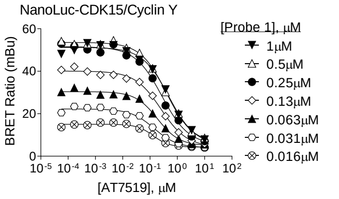

[Probe 1], µM	1	0.5	0.25	0.13	0.063	0.031	0.016
IC_{50} (µM)	0.49	0.40	0.27	0.25	0.19	0.14	0.13

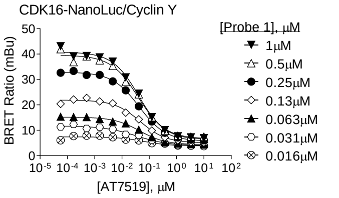

[Probe 1], µM	1	0.5	0.25	0.13	0.063	0.031	0.016
IC_{50} (µM)	0.041	0.039	0.042	0.046	0.045	0.033	0.062

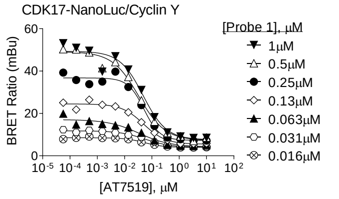

[Probe 1], µM	1	0.5	0.25	0.13	0.063	0.031	0.016
IC_{50} (µM)	0.048	0.033	0.051	0.049	0.034	0.055	0.052

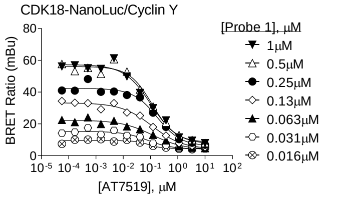

[Probe 1], µM	1	0.5	0.25	0.13	0.063	0.031	0.016
IC_{50} (µM)	0.10	0.12	0.18	0.11	0.12	0.078	0.085

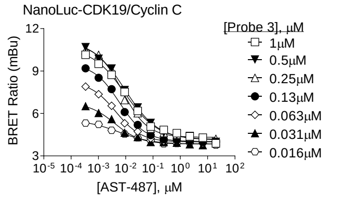

[Probe 1], µM	1	0.5	0.25	0.13	0.063	0.031	0.016
IC_{50} (µM)	0.010	0.0094	0.0074	0.0059	0.0043	0.0039	0.0055

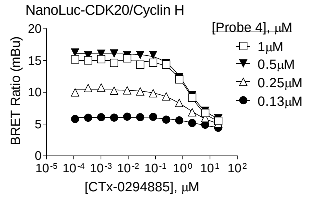

[Probe 4], µM	1	0.5	0.25	0.13
IC_{50} (µM)	1.6	1.4	1.5	N/D

Supplementary Figure 3. Impact of Energy Transfer Probe Concentration on the Apparent Compound IC_{50} for Preferred CDK/Probe Pairs. Energy probes were characterized as described in the methods section. Briefly, a dilution series of test compound was added to HEK-293 cells containing an increasing concentration of energy transfer probe. The BRET ratio was measured as a function of test compound dose and the compound IC_{50} at each probe concentration was interpolated using equation 1 (see methods section). The data in each plot was collected in a single biological experiment (n = 1), with each individual data point as a single technical replicate (n = 1). In all cases, optimized probe concentrations chosen for CDK compound profiling in this study (Supplementary Table 1) provided a balance of sufficient assay window with minimal right-shifting of apparent compound IC_{50}. Source data are provided as a Source Data File.

Supplementary Figure 4

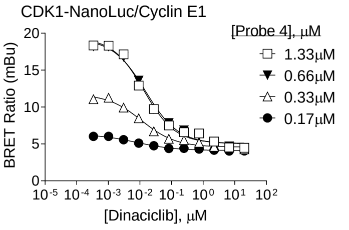

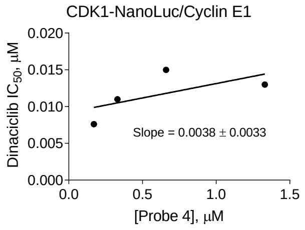

[Tracer], µM	1.33	0.66	0.33	0.17
IC_{50} (µM)	0.013	0.015	0.011	0.0076

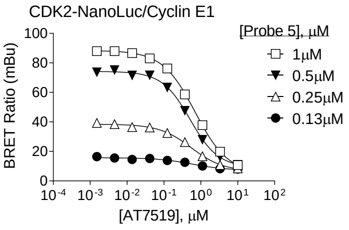

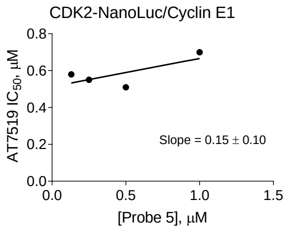

[Probe 5], µM	1	0.5	0.25	0.13
IC_{50} (µM)	0.70	0.51	0.55	0.58

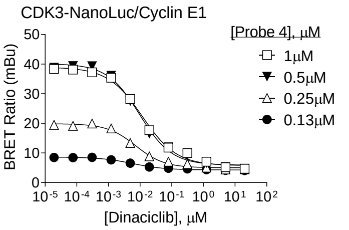

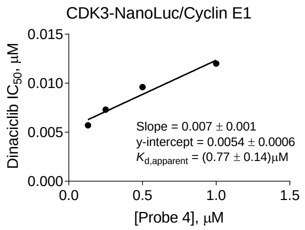

[Probe 4], µM	1	0.5	0.25	0.13
IC_{50} (µM)	0.012	0.0096	0.0073	0.0057

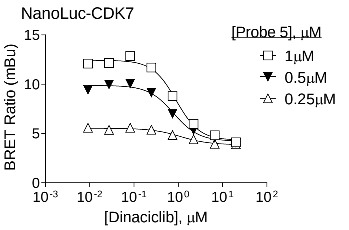

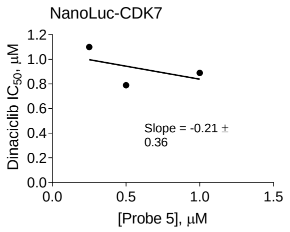

[Probe 5], µM	1	0.5	0.25
IC_{50} (µM)	0.89	0.79	1.1

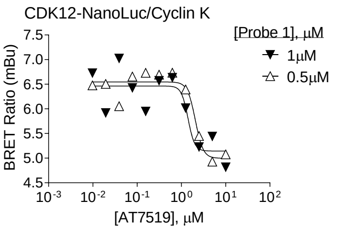

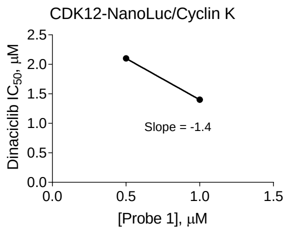

[Probe 1], µM	1	0.5
IC_{50} (µM)	1.4	2.1

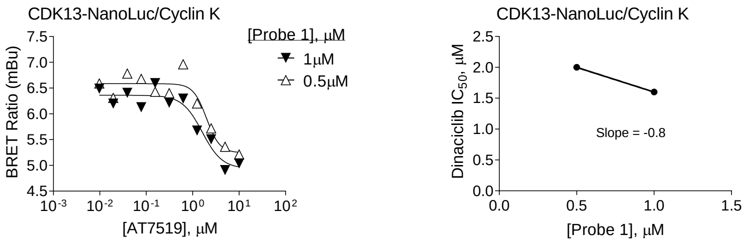

[Probe 1], μM	1	0.5
IC_{50} (μM)	1.6	2

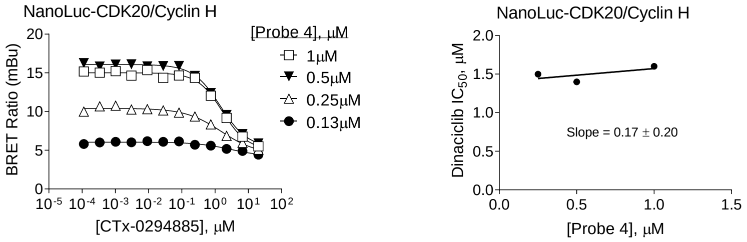

[Probe 4], μM	1	0.5	0.25	0.13
IC_{50} (μM)	1.6	1.4	1.5	N/D

Supplementary Figure 4. Evaluation of Probe Saturation Behavior for CDKs with Undefined Potencies in Probe Titrations. Apparent probe saturation was evaluated by assessing the degree to which the probe established a linear Cheng-Prusoff relationship up to a concentration of 1μM (the functional performance limit for most probes used in this study, Supplementary Figure 5). Probes were characterized as described in the methods section. (Left Panels) A dilution series of test compound was added to HEK-293 cells containing an increasing concentration of energy transfer probe. The BRET ratio was measured as a function of test compound dose and the compound IC_{50} at each probe concentration was interpolated using Equation 1 (see methods section). The dose response curves in each plot were measured in a single biological experiment (n = 1), with each data point as a singlicate measurement (n = 1). (Right Panels) The IC_{50}s generated in the left panels from each dose response curve were replotted as a function of probe concentration, and the data was fit to the linearized Cheng-Prusoff equation (Supplementary Equation 1). Errors on the curve fit values represent the S.E. propagated from curve fitting in Graphphad Prism. In most cases, linear fits to the IC_{50} replots did not yield a meaningful Cheng-Prusoff relationship, as evidenced by the lack of either a slope meaningfully higher than "0" or a concentration-dependent increase in apparent compound IC_{50}, suggesting that the probe concentrations used for IC_{50} profiling (Supplementary Table 1) are subsaturating. In the case of the CDK3/Cyclin E1 assay, a subtle Cheng-Prusoff relationship was established with a $K_{d,apparent}$ of 0.77μM for probe 4, which is above the 0.5μM concentration of probe 4 chosen for compound profiling. See Supplementary Figure 5 for examples of targets where a linear cheng-prusoff relationship has been established. Source data are provided as a Source Data File.

Supplementary Figure 5

A

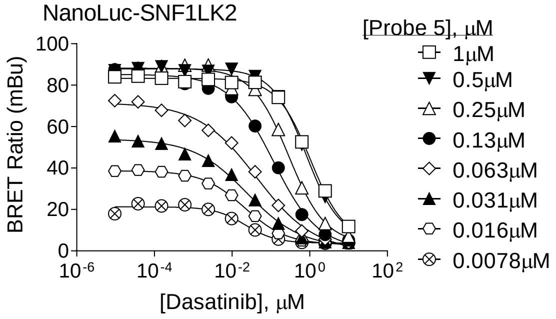

[Probe 5], µM	1	0.5	0.25	0.13	0.063	0.031	0.016	0.0078
IC_{50} (µM)	1.0	0.80	0.29	0.12	0.0430	0.025	0.021	0.022

B

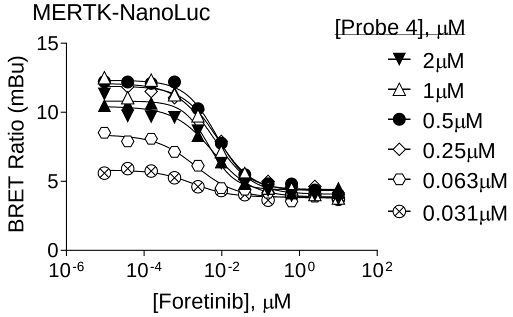

[Probe 4], µM	2	1	0.5	0.25	0.13	0.063	0.031
IC_{50} (µM)	0.0060	0.0064	0.0072	0.0078	0.0046	0.0022	0.0018

C

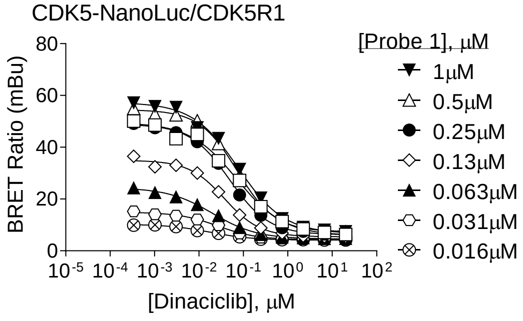

[Probe 1], µM	1	0.5	0.25	0.13	0.063	0.031	0.016
IC_{50} (µM)	0.077	0.072	0.048	0.037	0.020	0.026	0.018

D

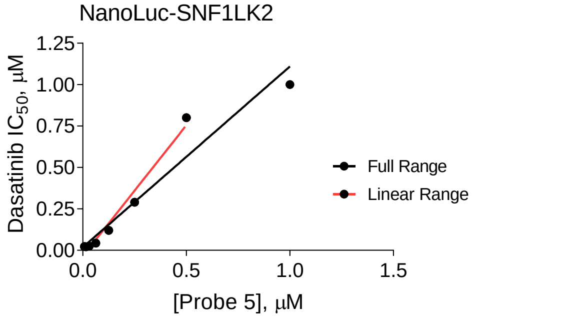

Range Fitted	Full Range	Linear Range
R^2	0.93	0.98

E

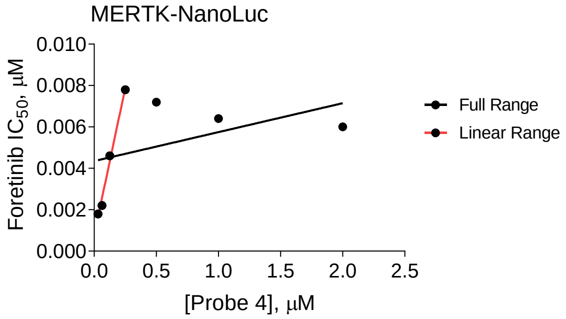

Range Fitted	Full Range	Linear Range
R^2	0.18	0.99

F

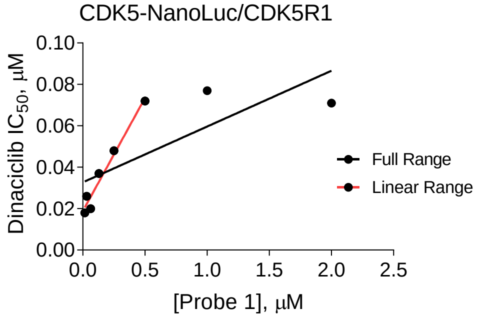

Range Fitted	Full Range	Linear Range
R^2	0.58	0.96

Supplementary Figure 5. Evaluation of the Functional Performance Limit for Representative Energy Transfer Probes. The functional performance limit for probes 1, 4, and 5 were evaluated by determining the concentration of probe at which an established Cheng-Prusoff relationship abruptly deviates from linearity. Probes were characterized as described in the methods section. (Panels A, B, and C) For each probe, a kinase target was evaluated that has been found to establish a linear Cheng-Prusoff relationship (CDK5-NanoLuc/CDK5R1 for probe 1, MERTK-NanoLuc for probe 4, and NanoLuc-SNF1LK2 for probe 5. A dilution series of test compound was added to HEK-293 cells containing an increasing concentration of energy transfer probe. The BRET ratio was measured as a function of test compound dose and the compound IC_{50} at each probe concentration was interpolated using Equation 1 (see methods section). The dose response curves in each plot were collected in a single biological experiment (n = 1), with each data point as a singlicate measurement (n = 1). (Panels D, E, and F) The IC_{50}s generated were replotted as a function of probe concentration, and the data was fit to the linearized Cheng-Prusoff equation (Supplementary Equation 1) over both the linear range ($R^2 > 0.96$) and non-linear range. All three probes demonstrated a pronounced deviation from linearity in the concentration range of 0.5−1µM. These data, combined with the formation of precipitate at even higher concentrations (data not shown), suggest a functional performance limit in the range of 0.5−1µM for these representative bodipy-based probes. Source data are provided as a Source Data File.

Supplementary Figure 6

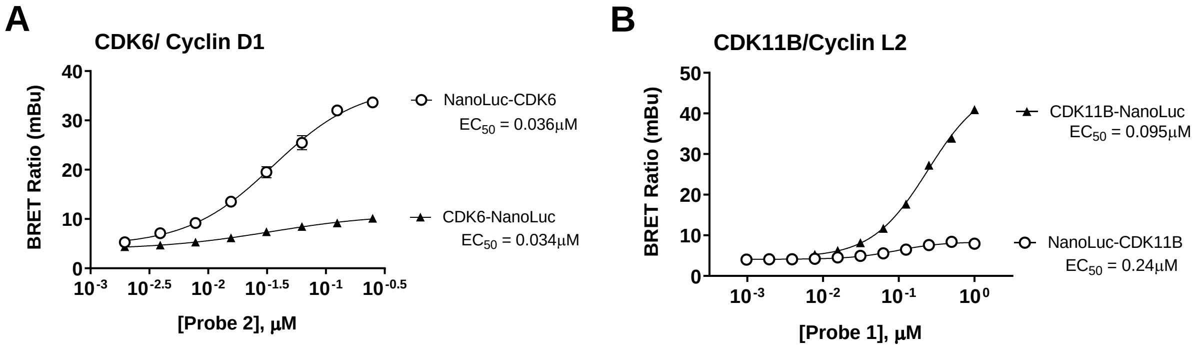

Supplementary Figure 6. Example effects of N- or C-terminal NanoLuc tagging on assay performance. Energy probes were characterized as described in the methods section. Briefly, energy probes were titrated onto HEK-293 cells expressing CDK/NanoLuc fusions up to a maximum concentration of 1µM. The BRET ratio was measured as a function of energy probe concentration. In the case of CDK6 (A), N- and C-terminal NanoLuc fusions demonstrated similar probe potency, but the N-terminal NanoLuc fusion demonstrated a vastly superior BRET signal. In the case of CDK11B (B), The C-terminal NanoLuc fusion demonstrated improved probe potency and a vastly superior BRET signal compared to the N-terminal NanoLuc fusion. In panels A and B, the dose response curves were measured in a single biological experiment (n = 1). In panel A, the individual points for the NanoLuc-CDK6 curve represent the mean ± S.D. of 4 technical replicates (n = 4) and the individual points for the CDK6-NanoLuc curve represent technical singlicates (n = 1). In panel B, the individual points for the CDK11B-NanoLuc curve represent the mean ± S.D. of 4 technical replicates (n = 4) and the individual points for the NanoLuc-CDK11B curve represent technical singlicates (n = 1). Source data are provided as a Source Data File.

Supplementary Figure 7

A

	CDK1	CDK1 + Cyclin E1
Probe 1	1.54	2.04
Probe 4	1.83	3.10
Probe 5	1.56	2.46

B

	CDK2	CDK2 + Cyclin E1
Probe 1	5.14	5.37
Probe 4	8.08	8.83
Probe 5	11.5	11.56

C

	CDK3	CDK3 + Cyclin E1
Probe 1	2.07	5.25
Probe 4	3.12	9.16
Probe 5	2.16	5.63

D

	CDK4	CDK4 + Cyclin D1
Probe 1	1.95	4.34
Probe 2	2.14	5.53
Probe 5	2.38	4.30

E

	CDK5	CDK5 + CDK5R1
Probe 1	1.44	4.60
Probe 4	5.09	5.55
Probe 5	8.67	9.46

F

	CDK6	CDK6 + Cyclin D1
Probe 1	1.79	3.05
Probe 2	6.52	4.09
Probe 5	1.85	3.14

G

	CDK7	CDK7 + Cyclin H
Probe 1	4.36	1.26
Probe 5	3.21	2.22

H

	CDK8	CDK8 + Cyclin C
Probe 3	1.81	5.08

I

	CDK9	CDK9 + Cyclin K
Probe 1	5.87	8.50
Probe 3	4.74	8.18
Probe 4	1.62	3.53
Probe 5	2.05	3.74

J

	CDK10	CDK10 + Cyclin L2
Probe 1	2.28	2.48
Probe 5	2.44	2.67

K

	CDK11A	CDK11A + Cyclin L2
Probe 1	5.08	5.38

L

	CDK11B	CDK11AB+ Cyclin L2
Probe 1	5.46	5.85

M

	CDK12	CDK12 + Cyclin K
Probe 1	1.32	1.25
Probe 4	1.29	1.39
Probe 5	1.38	1.44

N

	CDK13	CDK13 + Cyclin K
Probe 1	1.38	1.29
Probe 4	1.22	1.29
Probe 5	1.15	1.29

O

	CDK14	CDK14 + Cyclin Y
Probe 1	9.91	5.96
Probe 3	1.62	2.18
Probe 5	3.02	3.00

P

	CDK15	CDK15 + Cyclin Y
Probe 1	6.36	5.69
Probe 3	1.8	2.51
Probe 5	2.21	3.31

Q

	CDK16	CDK16 + Cyclin Y
Probe 1	7.91	5.63
Probe 3	5.69	6.4
Probe 4	3.17	2.21
Probe 5	7.44	4.83

R

	CDK17	CDK17 + Cyclin Y
Probe 1	12.04	6.27
Probe 3	7.56	8.31
Probe 4	2.57	2.21
Probe 5	9.19	6.44

S

	CDK18	CDK18 + Cyclin Y
Probe 1	12.52	8.23
Probe 3	5.17	6.22
Probe 4	2.58	3.25
Probe 5	8.59	8.15

T

	CDK19	CDK19 + Cyclin C
Probe 3	1.38	2.37

U

	CDK20	CDK20 + Cyclin H
Probe 4	2.48	2.93
Probe 5	4.71	5.61

Figure S7. Influence of Cyclin or Regulator Co-Expression on the BRET Ratio for each CDK Assay. For each CDK, the influence of cyclin co-expression on the BRET ratio was evaluated for up to 4 different energy transfer probes. Transfections were prepared containing 1 part NanoLuc fusion vector and 9 parts of either a cyclin/regulator vector (CDK + Cyclin samples) or a promoterless carrier DNA (CDK only samples). Probes were added to cells expressing CDK/NanoLuc fusions and/or cyclin regulators at a concentration of 0.5µM in the presence or absence of 20µM of the unlabeled parent compound to demonstrate specificity. For each probe/CDK pair, specific BRET is reported as a relative BRET signal by normalizing the raw BRET value for the tracer only sample to the raw BRET value in the presence of 20µM of the unlabeled parent compound. Values represent a single biological experiment (n = 1) with technical replicates (n = 2) for both the tracer only samples and the samples with 20µM of the unlabeled parent compound. Source data are provided as a Source Data File.

Supplementary Figure 8

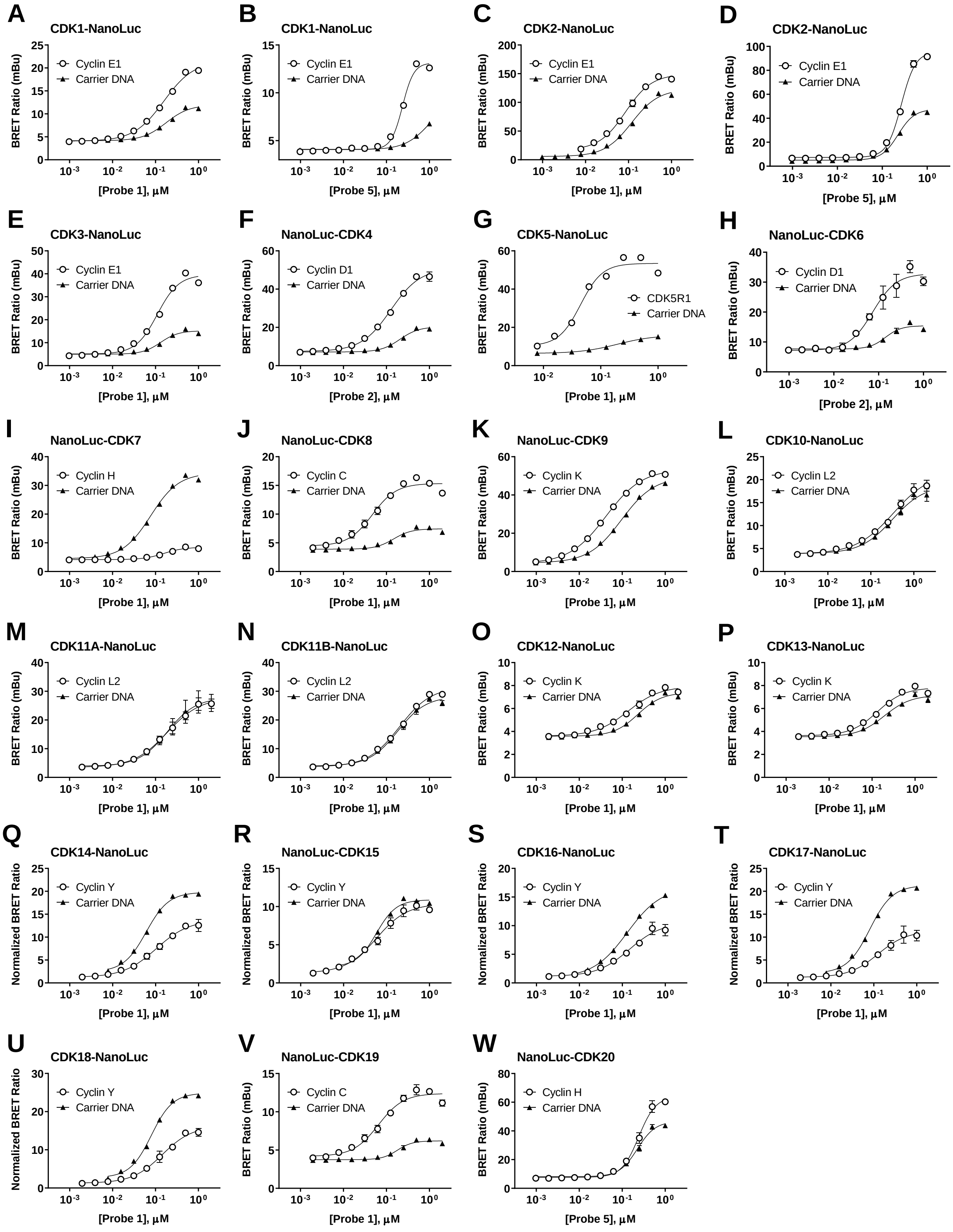

Supplementary Figure 8. Influence of Cyclin or Regulator Co-Expression on the Probe Dose-Response Behavior for Representative CDK Assays. Energy probes were characterized as described in the methods section. Briefly, HEK-293 cells were transfected with mixtures containing 1 part NanoLuc fusion vector and 9 parts of either cyclin/regulator vector (CDK + Cyclin samples) or a promoterless carrier DNA (CDK only samples). A dilution series of probe was added to the cells, after which BRET was measured and plotted as a function of probe concentration. In some cases where data was collected on different luminometers (e.g. CDKs 14−18), data is reported as signal fold change by normalizing each individual BRET value to the background BRET value on each instrument. In most cases, co-expression of a cyclin or regulator protein was found to influence the BRET value or probe potency. In the case of CDK10, CDK11A, and CDK11B, influence of co-expression on assay behavior was negligible. In virtually all cases (except for CDK7, which is explored further in Figure S9), cyclin or regulator co-expression was included for compound IC_{50} profiling. Dose response curves in each panel were measured in a single biological experiment (n = 1). Individual data points in Panel G (both curves), and Panels Q−U (Carrier DNA) represent technical singlicates (n = 1). The individual points in the Cyclin E1 curve for Panel C represent the mean of technical duplicates (n = 2). All other data points represent the mean ± S.D. of 4 technical replicates (n = 4). Source data are provided as a Source Data File.

Supplementary Figure 9

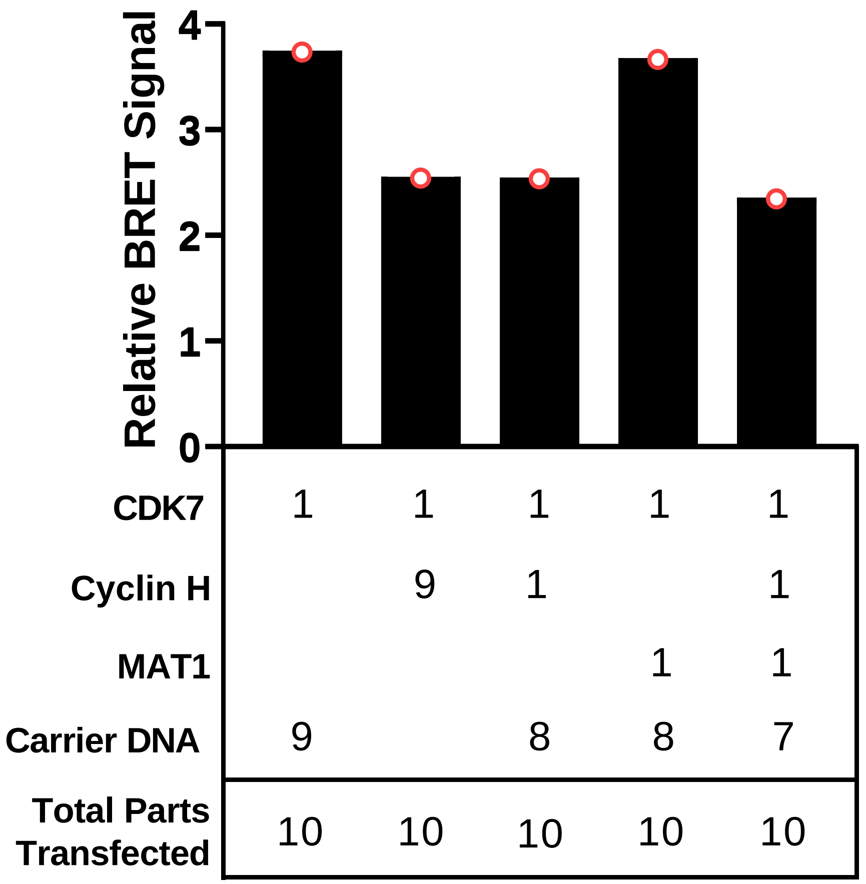

Supplementary Figure 9. Influence of Cyclin H and MAT1 Co-Expression on the CDK7 Assay. The influence of Cyclin H and/or MAT1 co-expression on the CDK7 assay was evaluated in HEK-293 cells using probe 5. NanoLuc-CDK7 fusion vector was co-transfected into HEK-293 cells with cyclin H, MAT1, and/or a promoterless carrier DNA at the ratios indicated. Probe 5 was added at a concentration of 1µM in the presence or absence of 20µM of Dinaciclib to demonstrate specificity. Specific BRET is reported as a relative BRET signal by normalizing the raw BRET value for the tracer only sample to the raw BRET value in the presence of 20µM of the unlabeled parent compound. Individual data points were measured in a single biological experiment (n = 1) and represent technical singlicates (n = 1). Cyclin H co-expression was found to reduce the BRET ratio regardless of MAT1 co-expression. MAT1 co-expression did not impact the BRET ratio regardless of Cyclin H co-expression. The highest BRET ratio was found when CDK7 was transfected in the absence of regulators. Source data are provided as a Source Data File.

Supplementary Figure 10

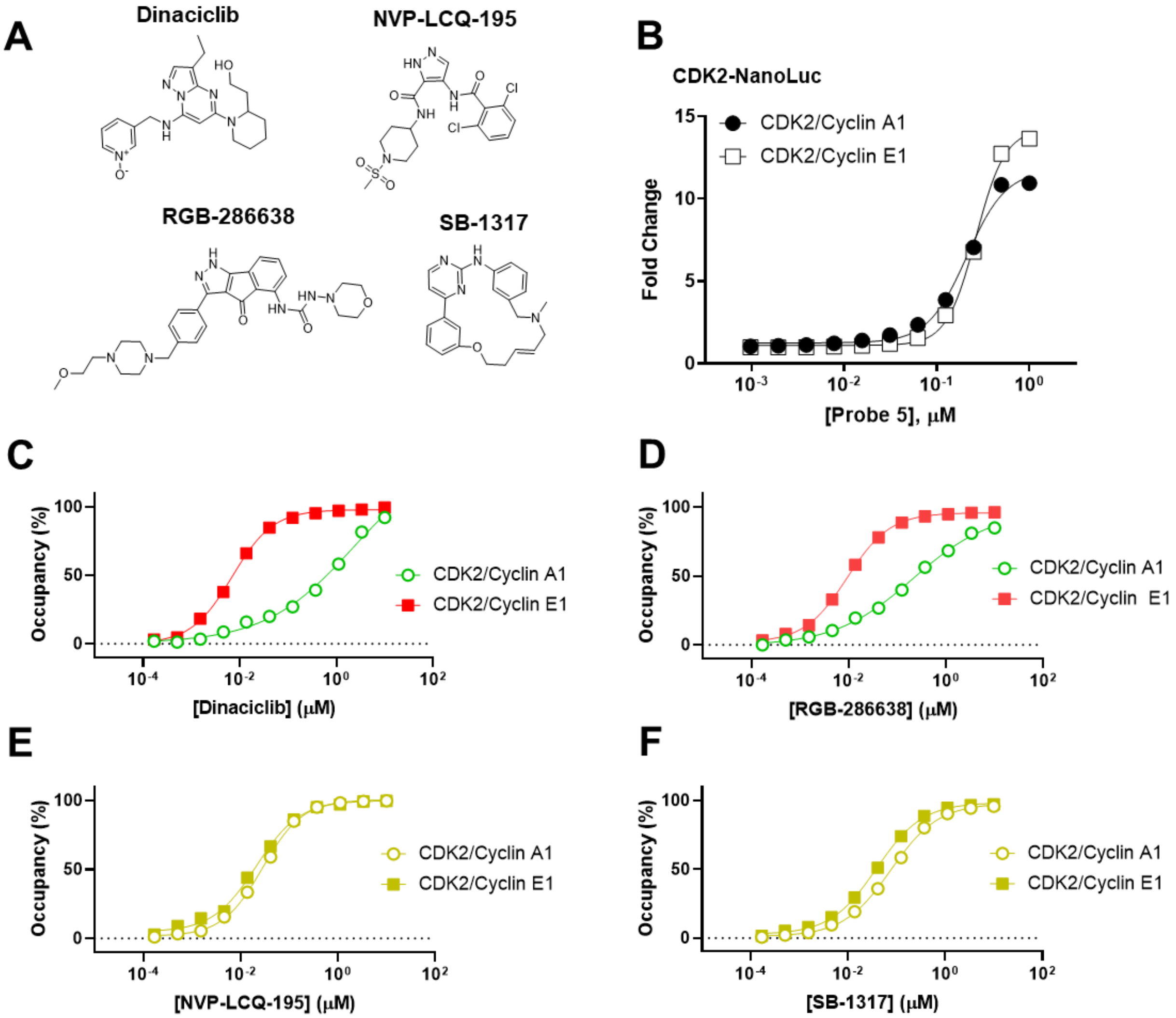

Supplementary Figure 10. Example pharmacological impacts for the CDK2 assay when co-expressing cyclin A1 vs E1. Compounds representing 4 different chemotypes (A) were selected for testing the impact of cyclin co-expression on compound potency for the CDK2 assay. (B) Probe 5 binding was evaluated in dose-response experiments for CDK2 assays with co-expression of either cyclin A1 or cyclin E1. Probe 5 was used at a concentration of 0.5µM in both assays for subsequent measurements of compound potency. Dinaciclib (C) and RGB-286638 (D) showed significantly left-shifted pharmacology with co-expression of cyclin E1 compared to cyclin A1, while NVP-LCQ-195 (E) and SB-1317 (F) showed similar pharmacology regardless of the which cyclin was co-expressed. Dose response curves in panel B were collected in a single biological experiment (n = 1) and individual data points represent the mean of technical quadruplicates (n = 4). Dose response curves in panels C−F are the mean of two independent biological experiments (n = 2). Individual points in panels C−F represent the mean of duplicates from two independent biological experiments (n = 2). Source data are provided as a Source Data File.

Supplementary Figure 11

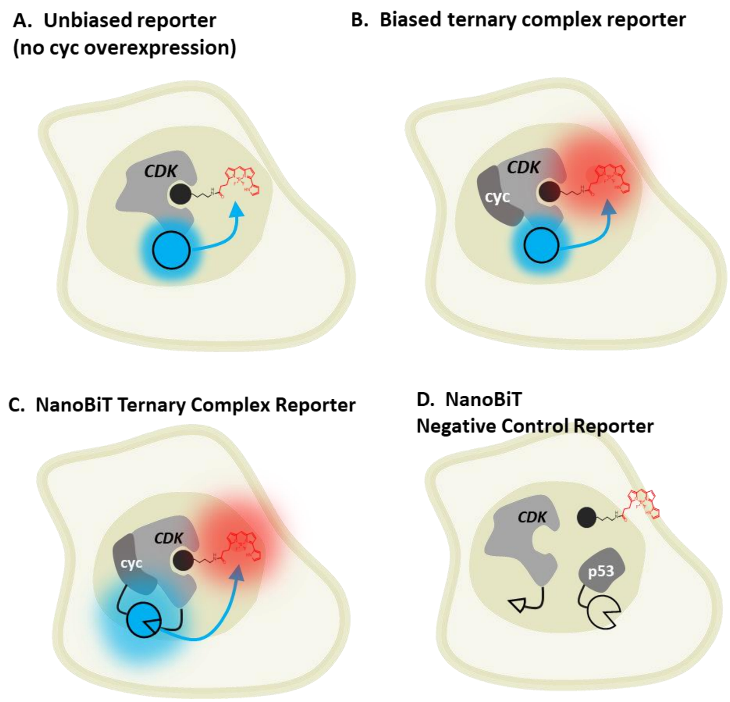

Supplementary Figure 11. NanoBiT target engagement assay design and impact of cyclin overexpression on CDK2 pharmacology. (A) Unbiased CDK2-NanoLuc target engagement in absence of cyclin overexpression. (B) Biased target engagement via overexpression of cyclin E1. (C) Selective target engagement at ternary complexes composed of CDK2/cyclin E1 with Probe 5 using a NanoBiT complementation-based reporter. (D) Negative control NanoBiT reporter.

Supplementary Figure 12

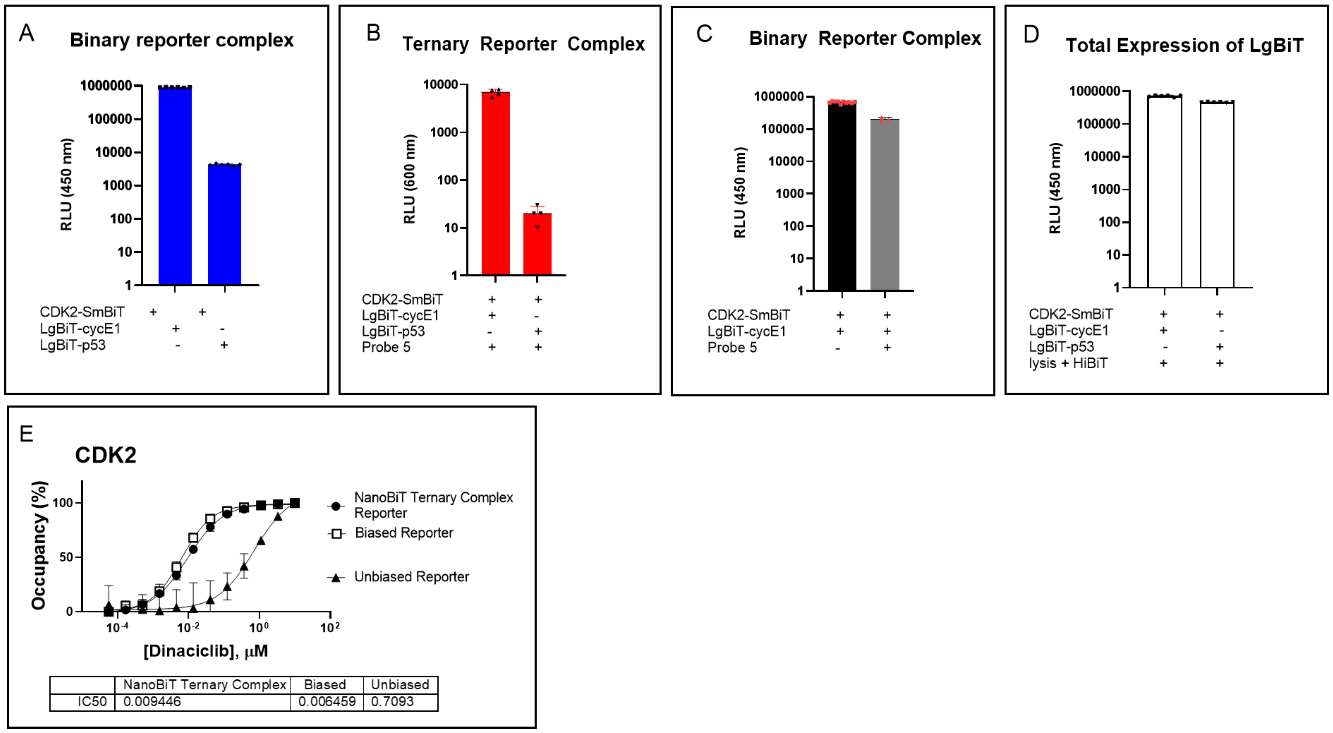

Supplementary Figure 12. Selectively quantifying intracellular engagement at CDK2/cyclin E1 complexes using a NanoBiT complementation reporter. (A) Luminescence from intracellular CDK2/cyclin E1 NanoBiT complexes compared to CDK2/p53 negative control complexes. Using NanoBiT, luminescence is conditionally generated upon formation of the intended protein-protein interaction[2]. Using NanoBiT as a binary reporter, CDK2-SmBiT/LgBiT-cyclin E1 expression generated >100-fold increase in luminescence compared to negative control (CDK2-SmBiT/LgBiT-p53) reporter constructs, thus supporting formation of the intended CDK/cyclin complex (Supplementary Figure 11). Individual bars represent the mean ± S.D. of 6 technical replicates (n = 6) for a single biological experiment (n = 1). (B) Ternary complex luminescence (600 nm) from CDK2/cyclin E1 NanoBiT complexes versus CDK2/p53 negative control complexes. Upon addition of energy transfer probe 5, BRET acceptor luminescence increased to a commensurate level (Supplementary Figure 11). Individual bars represent the mean ± S.D. of 4 technical replicates (n = 4) for a single biological experiment (n = 1). (C) Impact of CDK2/ cyclin E1 NanoBiT luminescence from Probe 5. Energy transfer probe modestly impacted the BRET donor luminescence of the binary signal generated between the CDK2/cyclin interaction, indicating that the interaction remains largely intact. Individual bars represent the mean ± S.D. of 10 technical replicates (left bar, n = 10) or 3 technical replicates (right bar, n = 3) for a single biological experiment (n = 1). (D) Total LgBiT expression from LgBiT-cyclin E1 vs LgBiT-p53. Expression level of LgBiT-cyclin E1 and LgBiT-p53 were comparable. Individual bars represent the mean ± S.D. of 6 technical replicates (n = 6) for a single biological experiment (n = 1). (E) Potency of dinaciclib at CDK2/cyclin E1 complexes, versus CDK2 in the absence of cyclin E1 overexpression. The resulting ternary reporter system thus serves as a luminescent intracellular complex to conditionally monitor engagement to the intended CDK/cyclin interface. To determine if the pharmacology of such complexes may diverge from un-complexed CDK,

target engagement was evaluated with dinaciclib (a known inhibitor of CDK2) in the presence or absence of cyclin overexpression. For dinaciclib, engagement potency at CDK2/cyclin E1 NanoBiT complexes matched closely with results observed with the biased cyclin E1 co-expression strategy (Supplementary Figure 11). In contrast, co-expression of CDK2-NanoLuc with a promoterless carrier DNA yielded engagement potency for dinaciclib that was nearly 100-fold right-shifted compared to the CDK2/cyclin E1 complexes. Dose response curves were measured in a single biological experiment (n = 1), and individual data points represent the mean of technical quadruplicates (n = 4) for the biased and unbiased curves, and a technical singlicate (n = 1) for the NanoBiT ternary complex curve. Source data are provided as a Source Data File.

Supplementary Figure 13

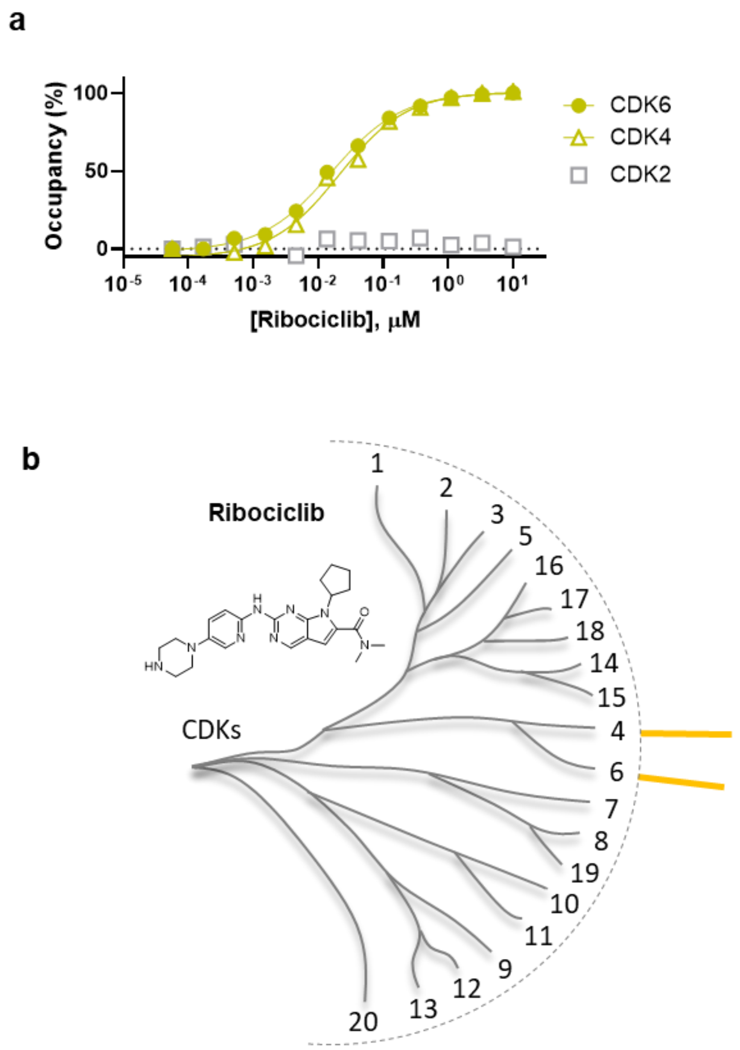

Supplementary Figure 13. Live cell engagement potency for CDK4/6 with ribociclib (a). CDK2 is included in each graph for comparative reference. Dose response curves are singlicates representative

of 3 independent biological experiments (n = 3), with individual data points as technical singlicates (n = 1). Live cell selectivity dendrograms for ribociclib (b). Source data are provided as a Source Data File.

Supplementary Figure 14

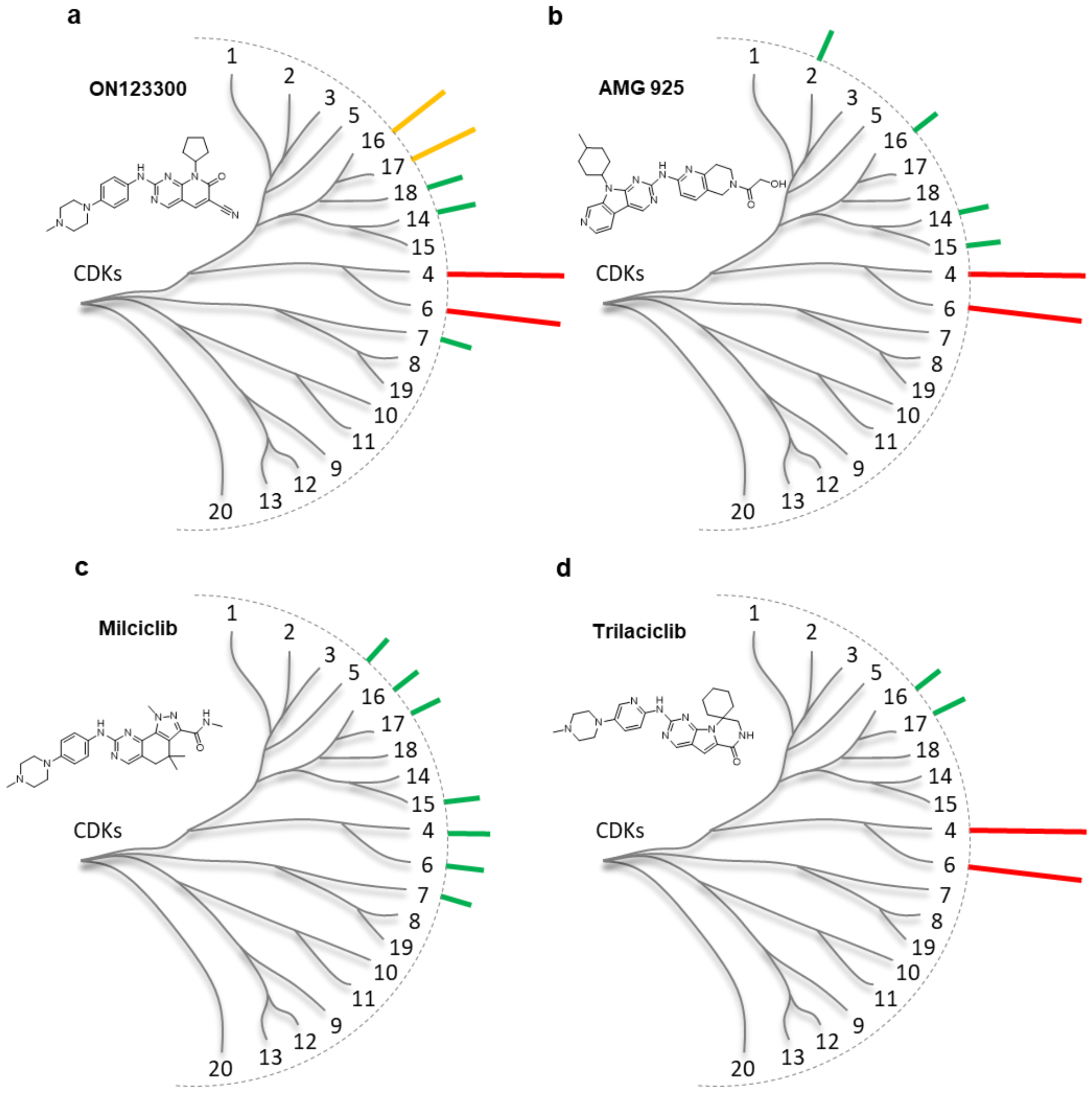

Supplementary Figure 14. Live cell selectivity dendrograms for CDK inhibitors ON123300 (a), AMG 925 (b), Milciclib (c), and Trilaciclib (d). Source data are provided as a Source Data File.

Supplementary Figure 15

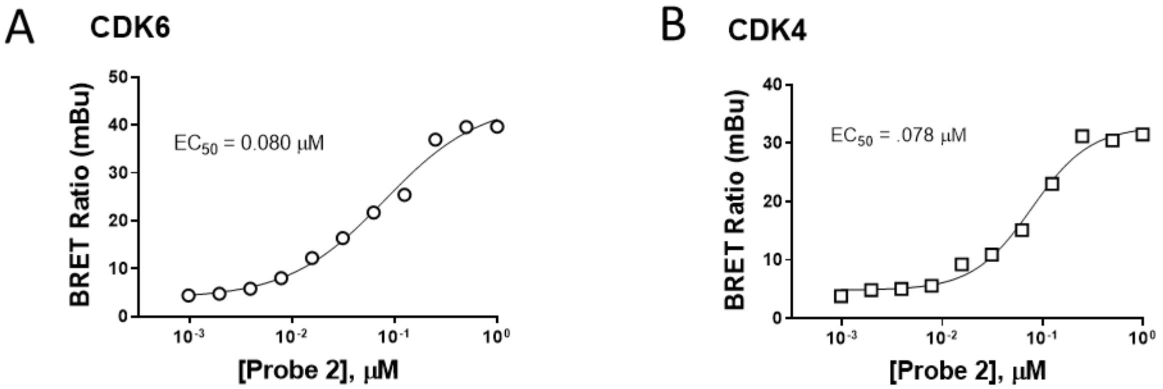

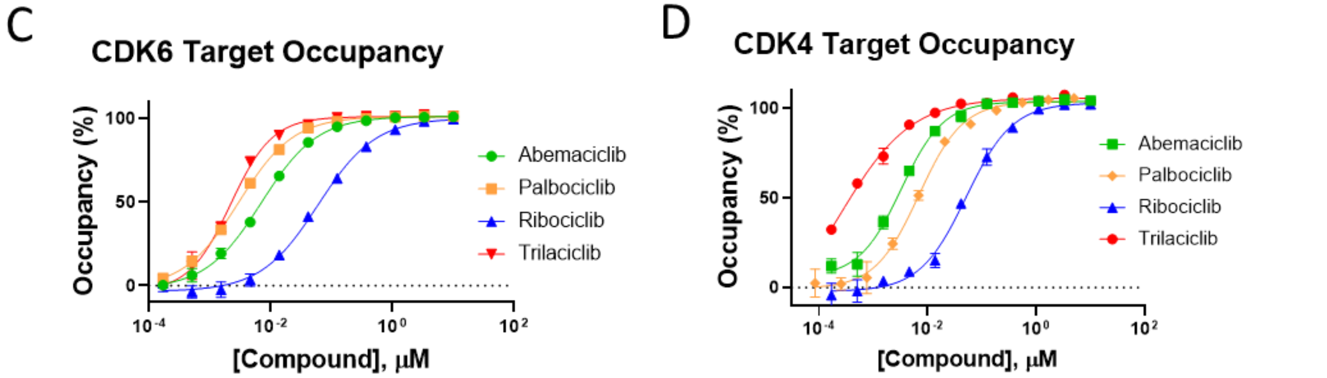

Compound	Abemaciclib	Palbociclib	Ribociclib	Trilaciclib
IC_{50} (μM)	0.0070	0.0030	0.060	0.0022

Compound	Abemaciclib	Palbociclib	Ribociclib	Trilaciclib
IC_{50} (μM)	0.0032	0.0069	0.051	0.0004

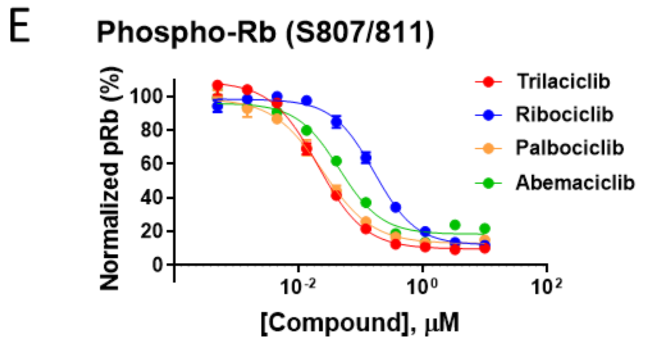

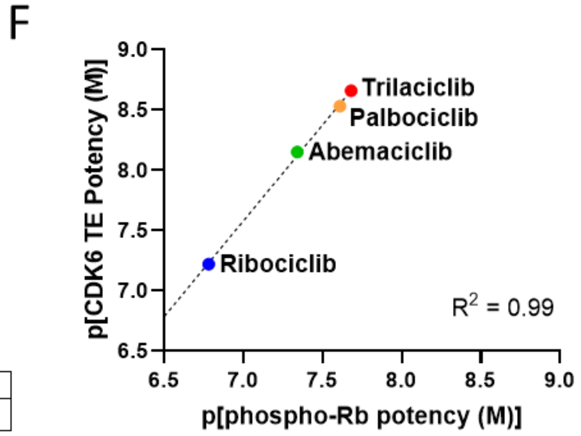

Compound	Abemaciclib	Palbociclib	Ribociclib	Trilaciclib
IC_{50} (μM)	0.046	0.025	0.17	0.021

Supplementary Figure 15. Comparison of potencies of CDK4 and CDK6 engagement with inhibition of Rb phosphorylation in MCF7 Cells. Apparent affinity of probe 2 was determined in MCF7 cells transiently transfected with Cyclin D1 and either CDK6 (A) or CDK4 (B). Target engagement was analyzed for CDK4/6 selective inhibitors in MCF7 cells for CDK6 (C) and CDK4 (D). Endogenous phospho-Rb analysis was performed with a homogenous luminescent immunoassay[5] in MCF7 cells with selective CDK4/6 inhibitors (E). Correlation of potencies [p(IC_{50})] was observed between CDK6 target occupancy and inhibition of phospho-Rb in MCF-7 cells (F). Engagement potencies for CDK4 were stronger than for CDK6, supporting CDK6 engagement may be a limiting factor in pharmacological inhibition of phospho-Rb in MCF-7 cells. All dose response curves were measured in a single biological experiment (n = 1). Individual data points in panels A and B represent technical singlicates (n = 1). Individual data points in panels C and D represent the mean ± S.D. of technical quadruplicates (n = 4). Individual data points in panel E represent the mean ± S.D. of technical triplicates (n = 3). Individual data points in panel F represent the IC_{50} values (n = 1) measured in panels C and E. Source data are provided as a Source Data File.

Supplementary Figure 16

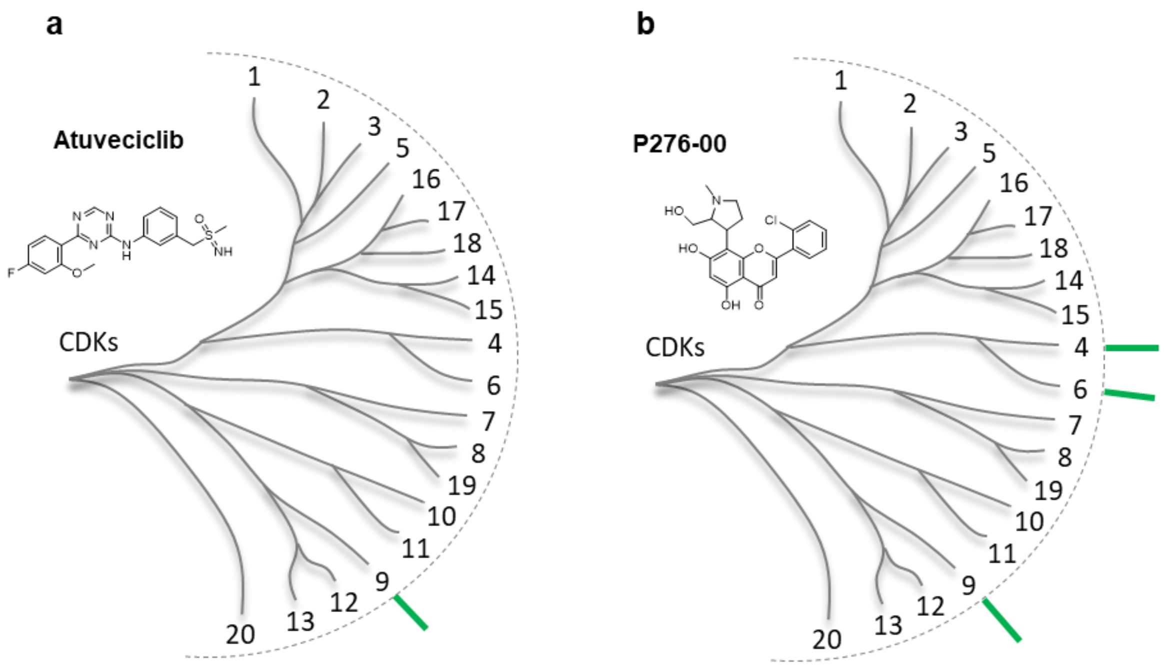

Supplementary Figure 16. Live cell selectivity dendrograms for CDK inhibitors Atuveciclib (a) and P276-00 (b). Source data are provided as a Source Data File.

Supplementary Figure 17

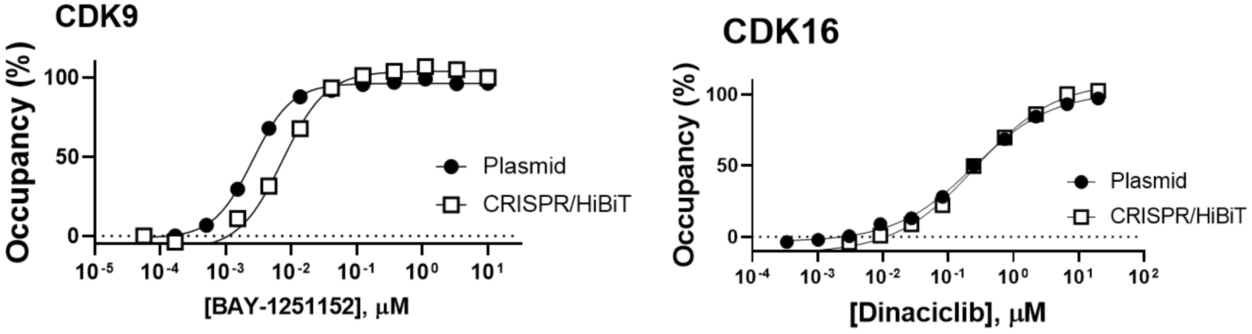

	CRISPR/HiBiT	Plasmid
IC50	0.007358	0.002569

	Plasmid	CRISPR/HiBiT
IC50	0.2457	0.2626

Supplementary Figure 17. Comparison of CDK9 and CDK16 target engagement using plasmid-based transfection of HEK293 cells versus HEK293 cells endogenously edited with HiBiT tagging. CDK9 and CDK16 were tagged at the endogenous locus using CRISPR-CAS9 as described above in the supplementary methods. Left; engagement of BAY-1251152 to CDK9. Right; engagement of dinaciclib to CDK16. All dose response curves were collected in a single biological experiment (n = 1). Individual data points represent technical singlicates (n = 1, CDK9 plasmid and both CDK16 curves) or the mean of technical duplicates (n = 2, CDK9 CRISPR/HiBiT curve). Source data are provided as a Source Data File.

Supplementary Figure 18

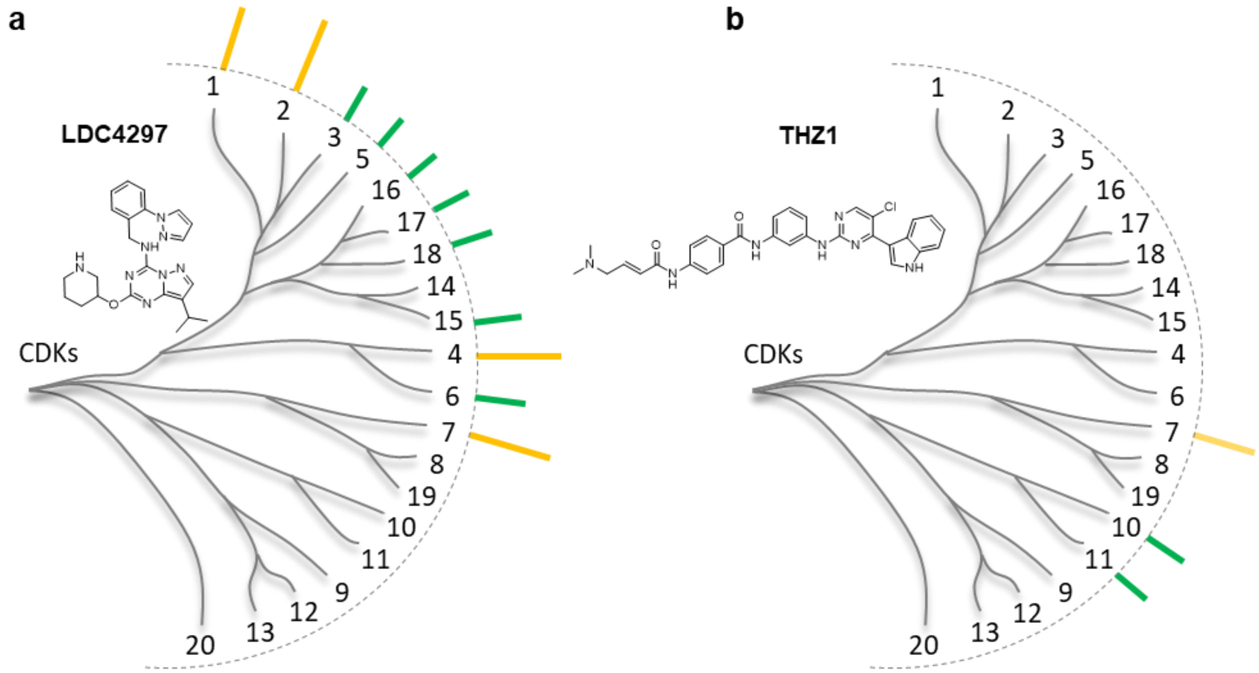

Supplementary Figure 18. Live cell selectivity dendrograms for CDK7 inhibitors LDC4297 (a) and THZ1 (b). Source data are provided as a Source Data File.

Supplementary Figure 19

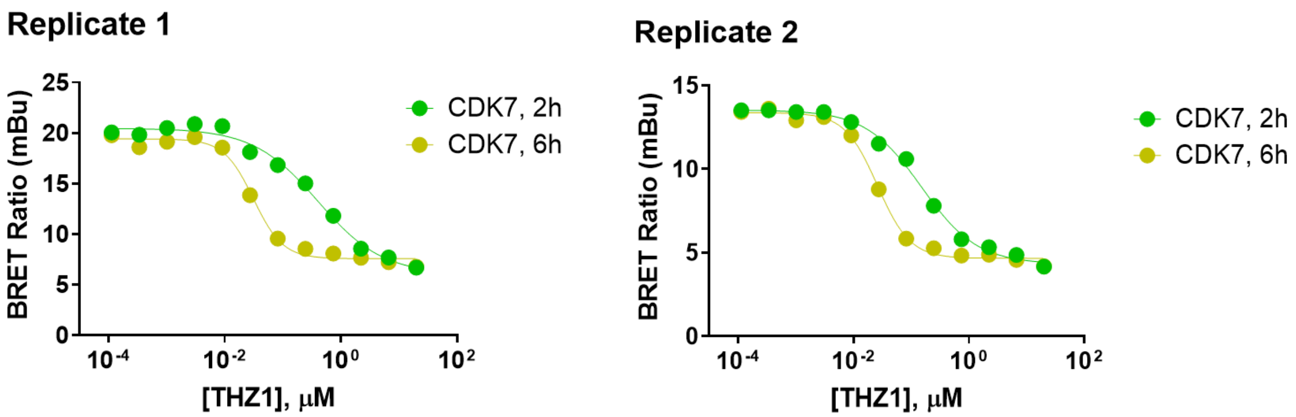

	2h	6h
IC50	0.41	0.031

	2h	6h
IC50	0.15	0.026

Supplementary Figure 19. Time-dependent potency for CDK7 Inhibitor THZ1. An extended incubation time of 6 hours results in a reproducible increase in potency compared to that observed at 2 hours, as demonstrated in two independent experiments (n = 2, replicates 1 and 2, respectively). Individual data points represent technical singlicates (n = 1). Source data are provided as a Source Data File.

Supplementary Figure 20

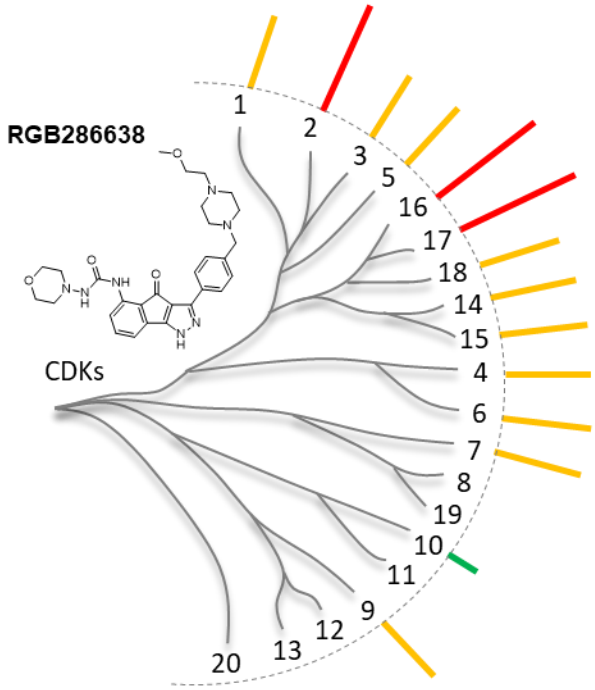

Supplementary Figure 20. Live cell selectivity dendrogram for inhibitor RGB286638. Source data are provided as a Source Data File.

Supplementary Figure 21

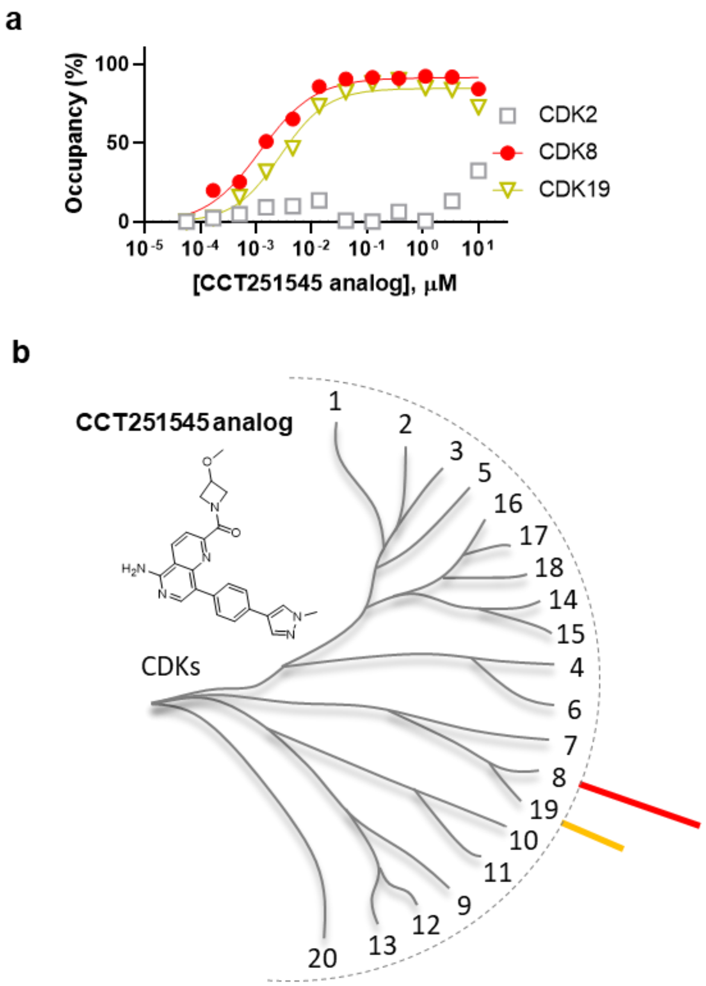

Supplementary Figure 21. Live cell engagement potency for CDK8/19 inhibitor CCT251545 analog (a). CDK2 is included in each graph for comparative reference. Dose response curves are singlicates representative of 3 independent biological experiments (n = 3), with individual data points as technical singlicates (n = 1). Live cell selectivity dendrograms for CCT251545 analog (b). Source data are provided as a Source Data File.

Supplementary Figure 22

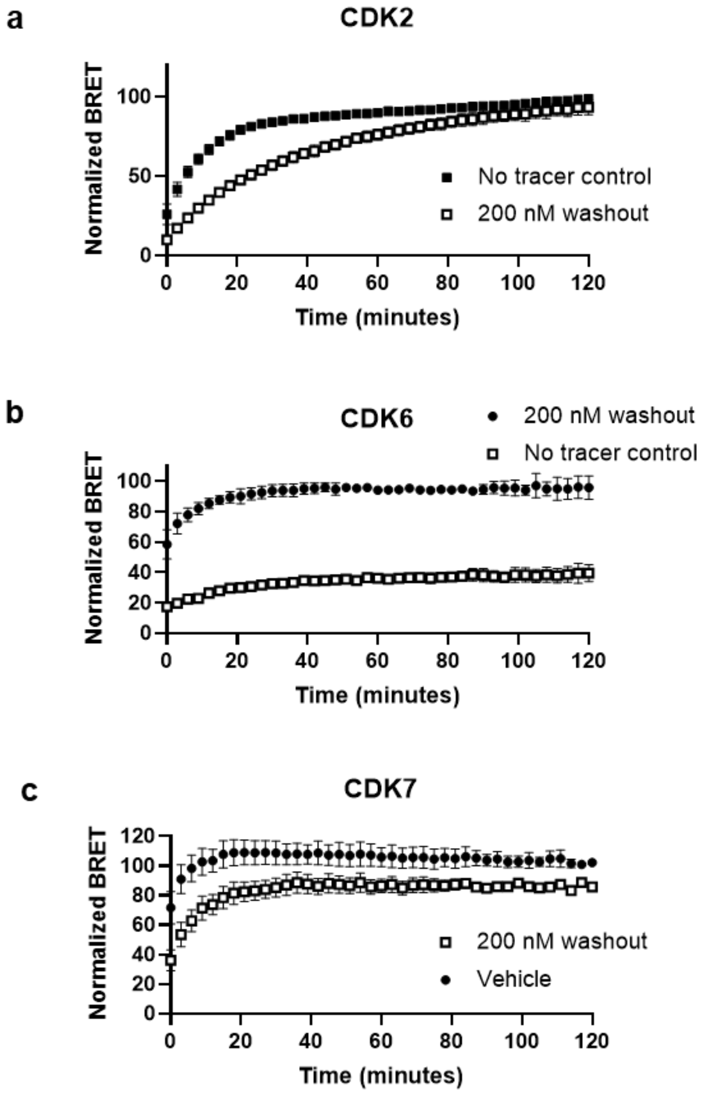

Supplementary Figure 22. Kinetic profiling of RGB286638 at CDK2 (a), CDK6 (b), and CDK7 (c). Residence time was qualitatively assessed by cellular washout as described in the method details and compared to association of energy transfer probe in cells treated with DMSO vehicle. RGB286638 shows protracted engagement at CDK6 and kinetic selectivity for CDK6 compared to CDK2 and CDK7. BRET ratios were normalized between the maximum BRET value vs BRET value in the absence of tracer. Kinetic traces and individual points are mean of 3 independent experiments ± S.E.M (n = 3). Source data are provided as a Source Data File.

Supplementary Figure 23

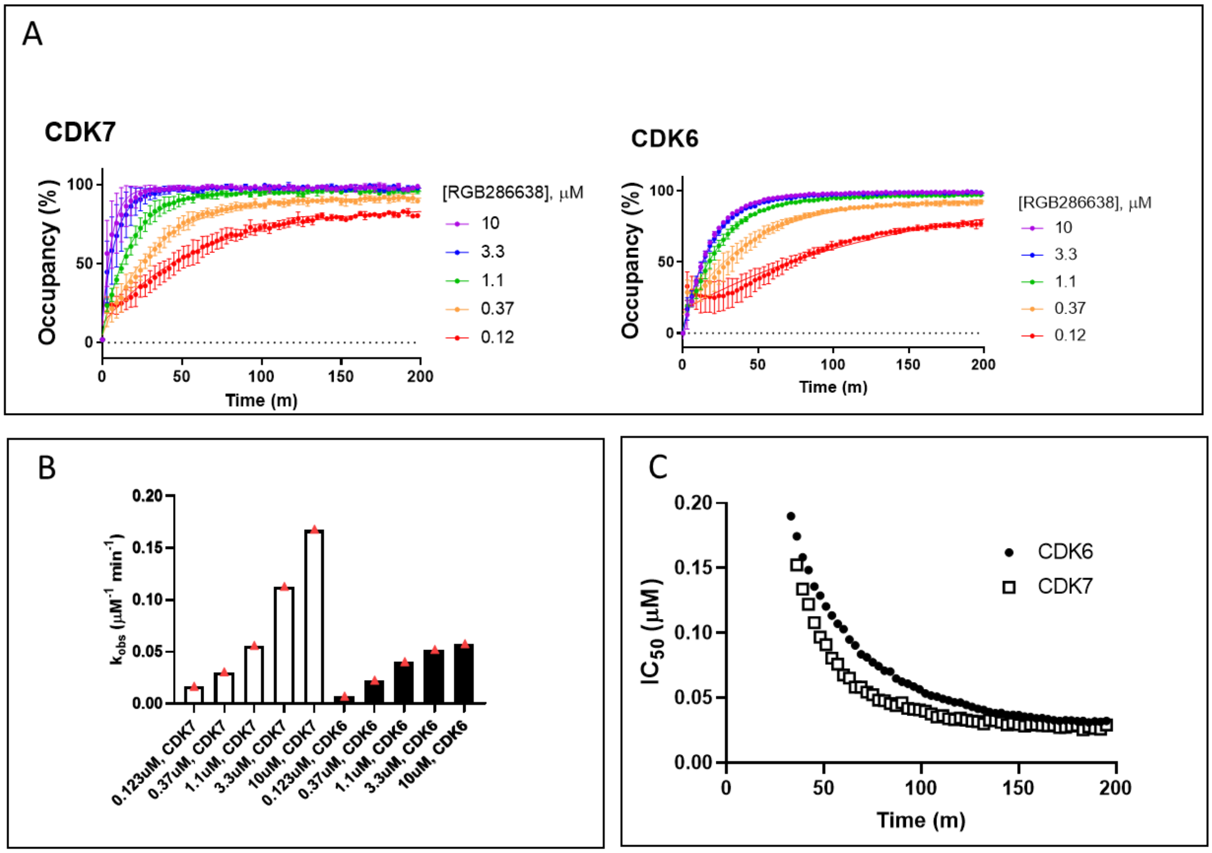

Supplementary Figure 23. Real-time analysis of target engagement of RGB286638 to CDK6 and CDK7 in live cells. (A) Real-time target engagement analysis of varying concentrations of RGB286638 against CDK7 (left) and CDK6 (right) to generate k_{obs}. Kinetic traces were collected in a single biological experiment (n = 1), where individual data points represent the mean ± S.D of technical triplicates (n = 3). (B) k_{obs} values (n = 1) were generated from panel A by fitting Supplementary Equation 2, and were then plotted as a function of compound dose, revealing slower engagement to CDK6 vs CDK7. (C) Analysis RGB286638 potency over time also revealed slower equilibration of RGB286638 to CDK6 vs CDK7. Kinetic traces were collected in a single biological experiment (n = 1), where Individual IC_{50} values collected at each timepoint represent technical singlicates (n = 1). Source data are provided as a Source Data File.

Supplementary Figure 24

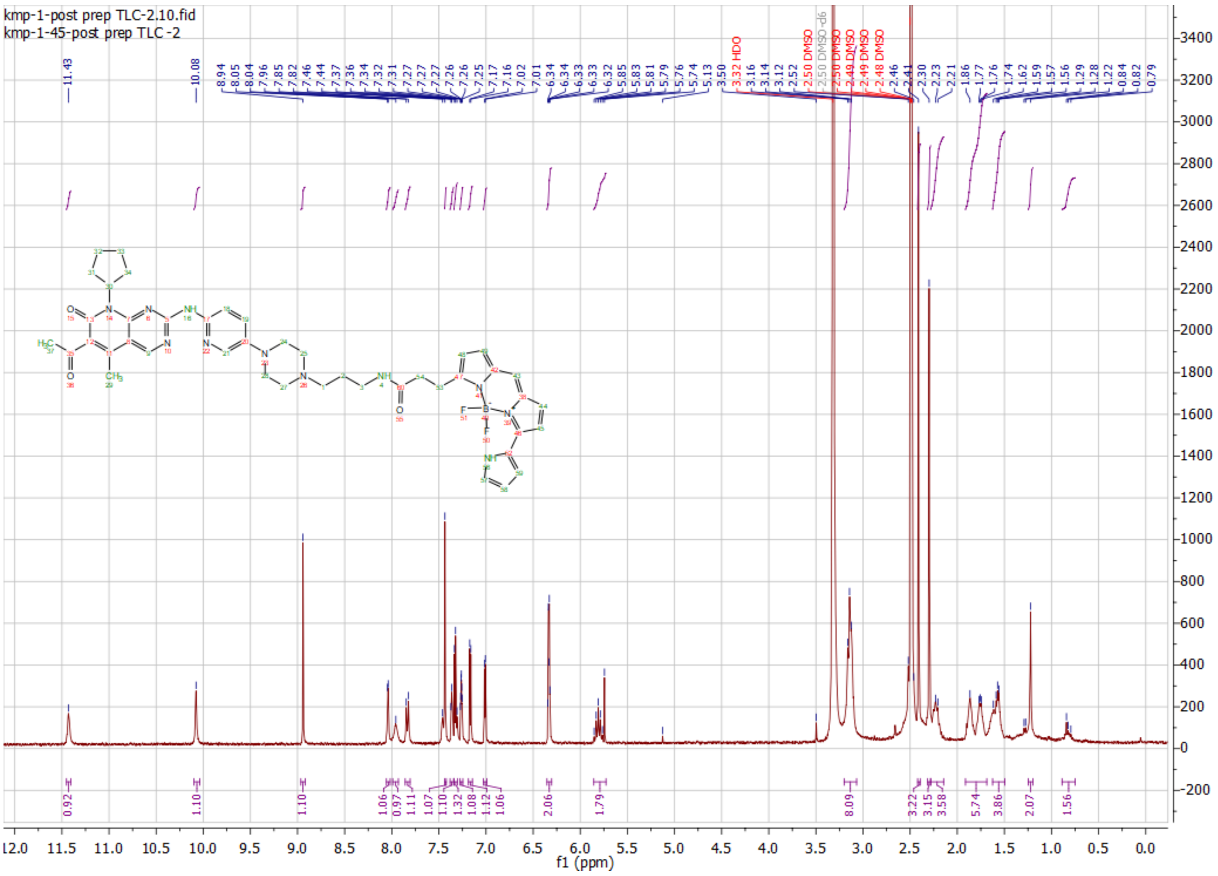

Supplementary Figure 24. ^{1}H-NMR of probe 2.

Supplementary Figure 25

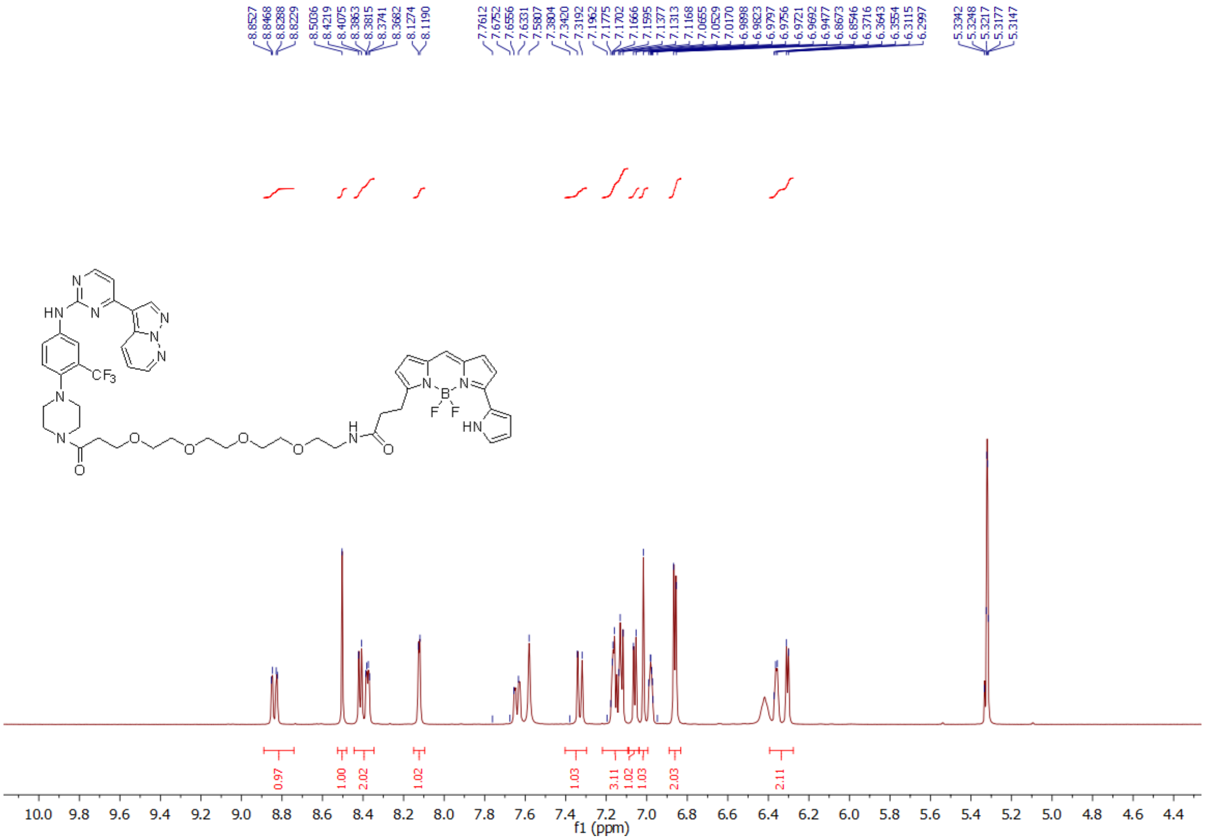

Supplementary Figure 25. ^{1}H-NMR of probe 3.

Supplementary Figure 26

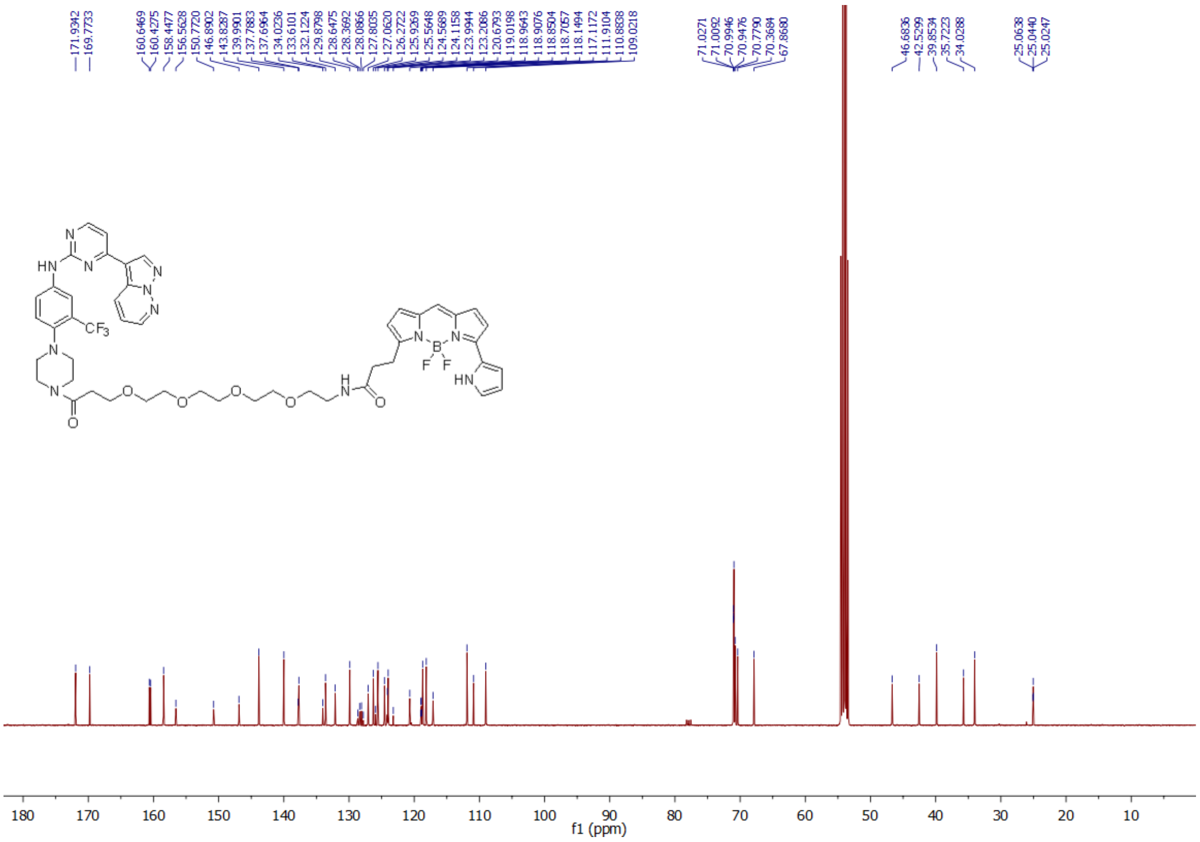

Supplementary Figure 26. ^{13}C-NMR of probe 3.

Supplementary Figure 27

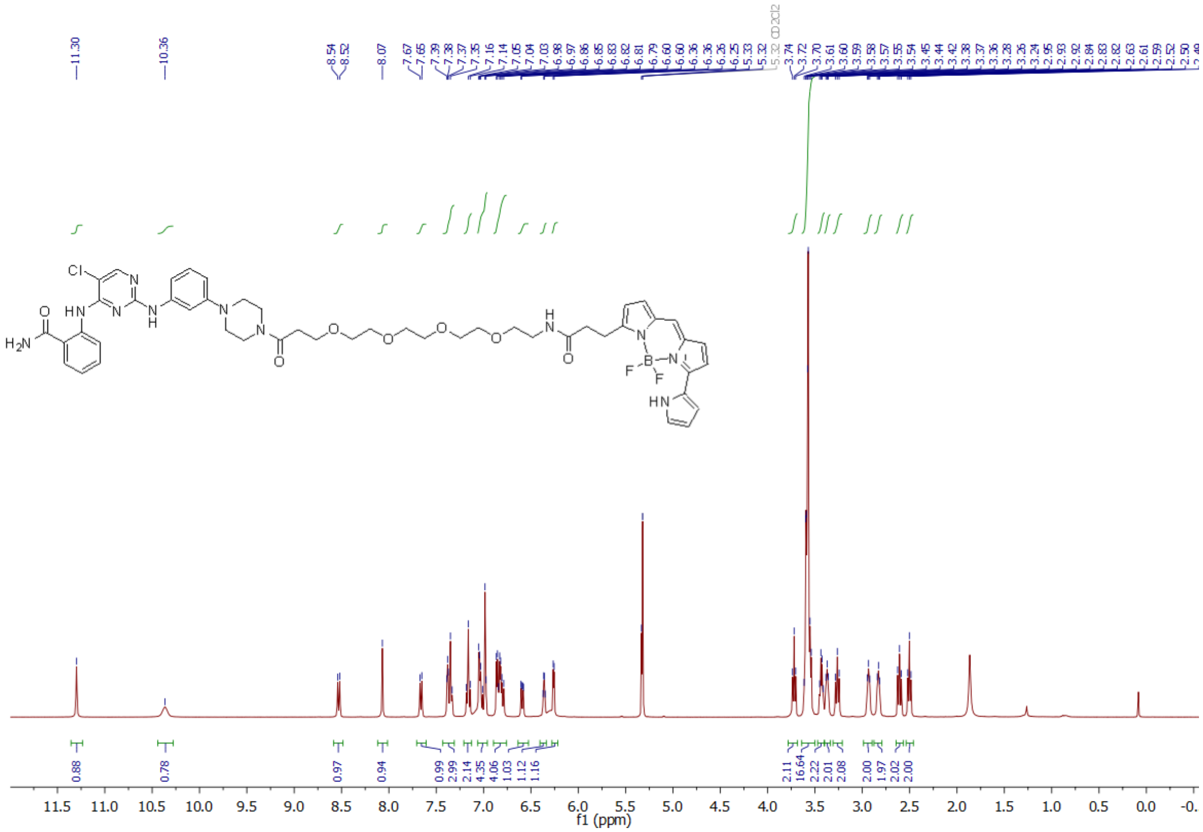

Supplementary Figure 27. ^{1}H NMR of probe 4.

Supplementary Figure 28

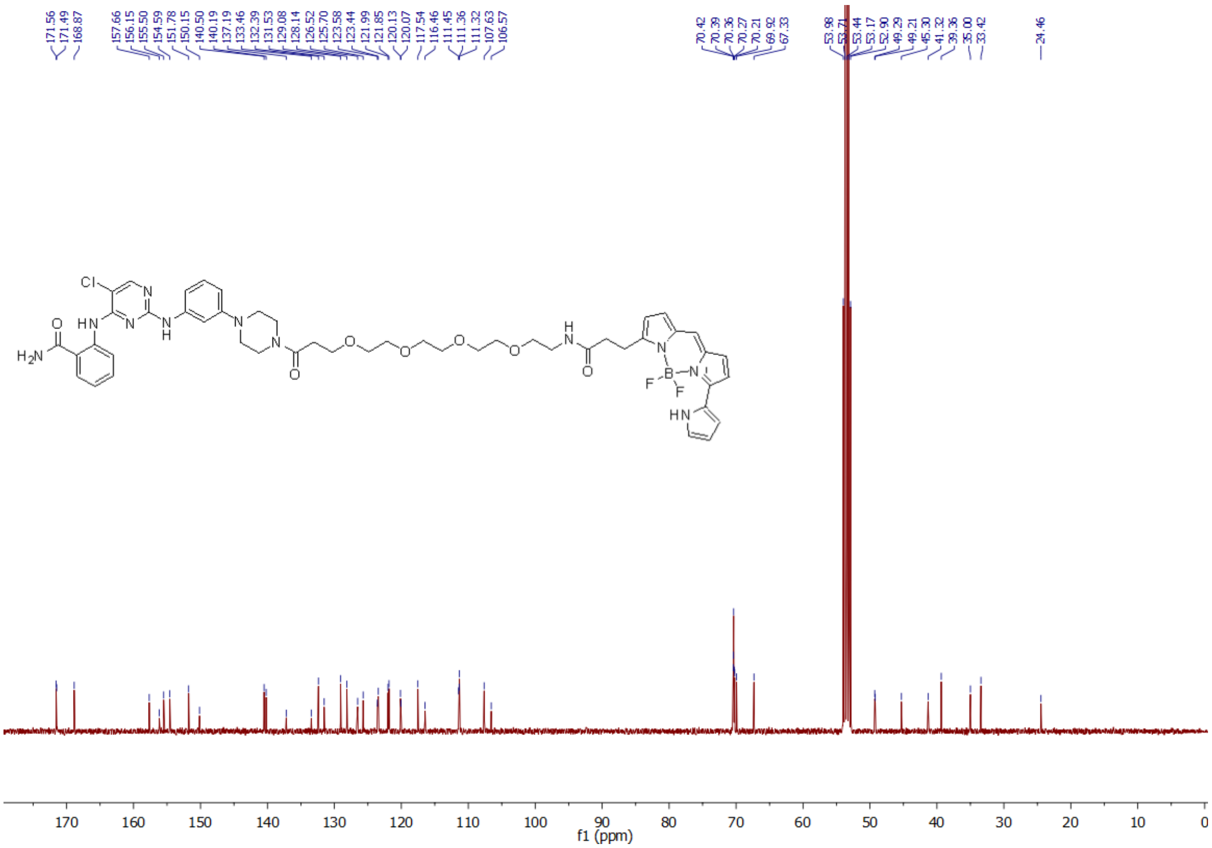

Supplementary Figure 28. ^{13}C-NMR of probe 4.

Supplementary Figure 29

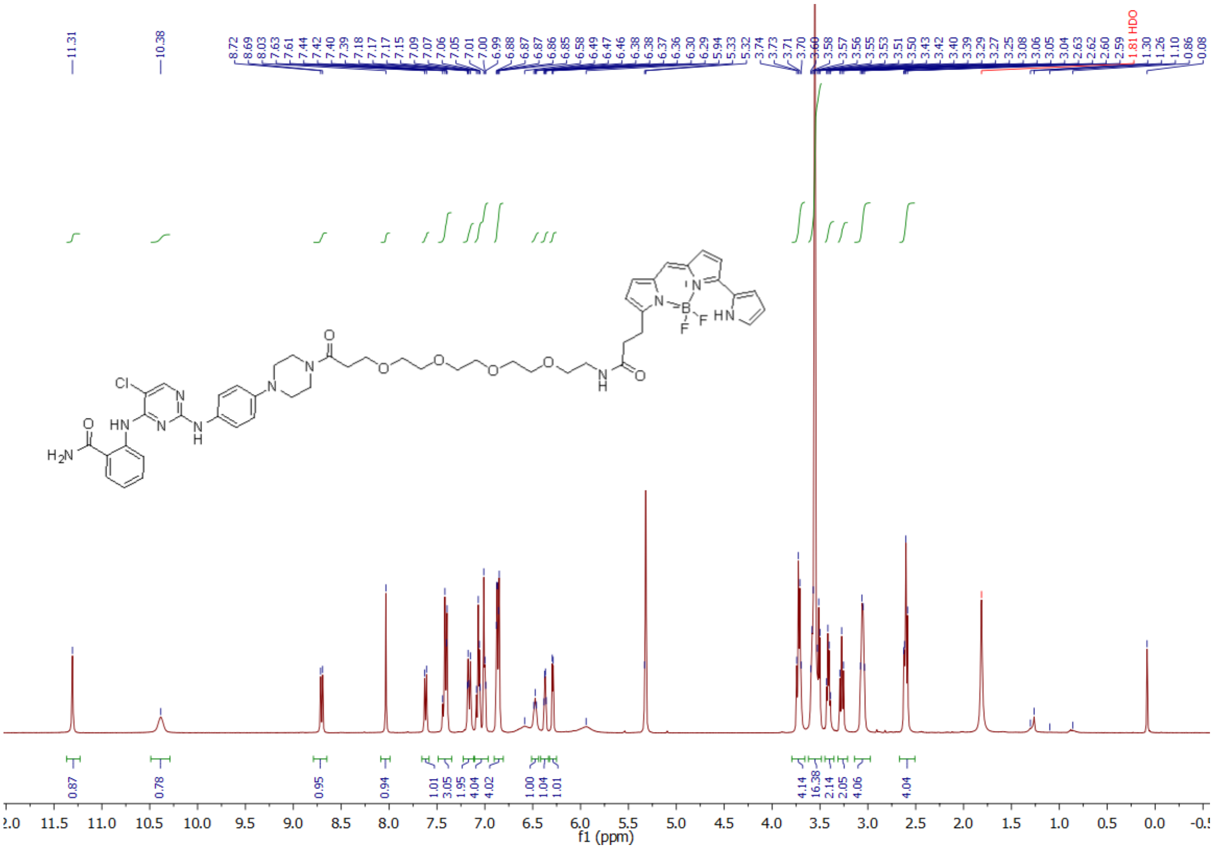

Supplementary Figure 29. ^{1}H-NMR of probe 5.

Supplementary Figure 30

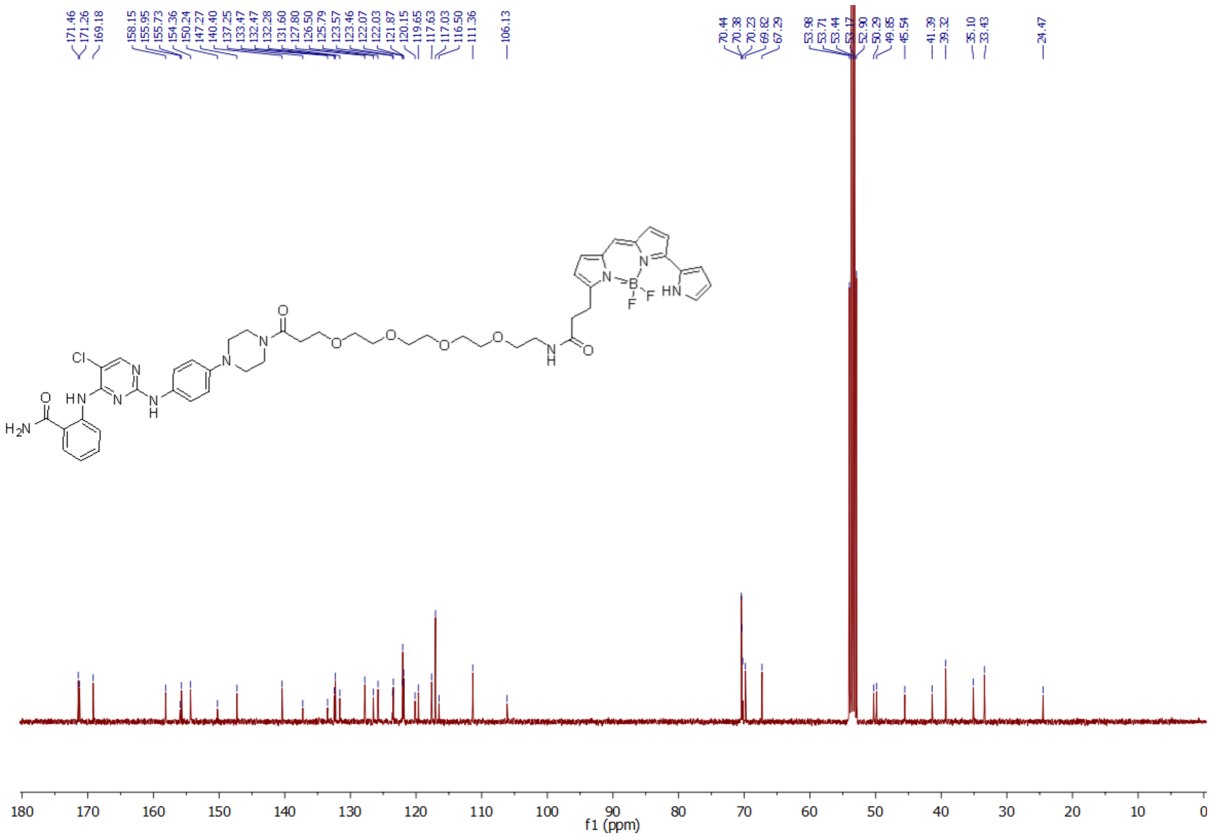

Supplementary Figure 30. ^{13}C-NMR of probe 5.

Supplementary Figure 31

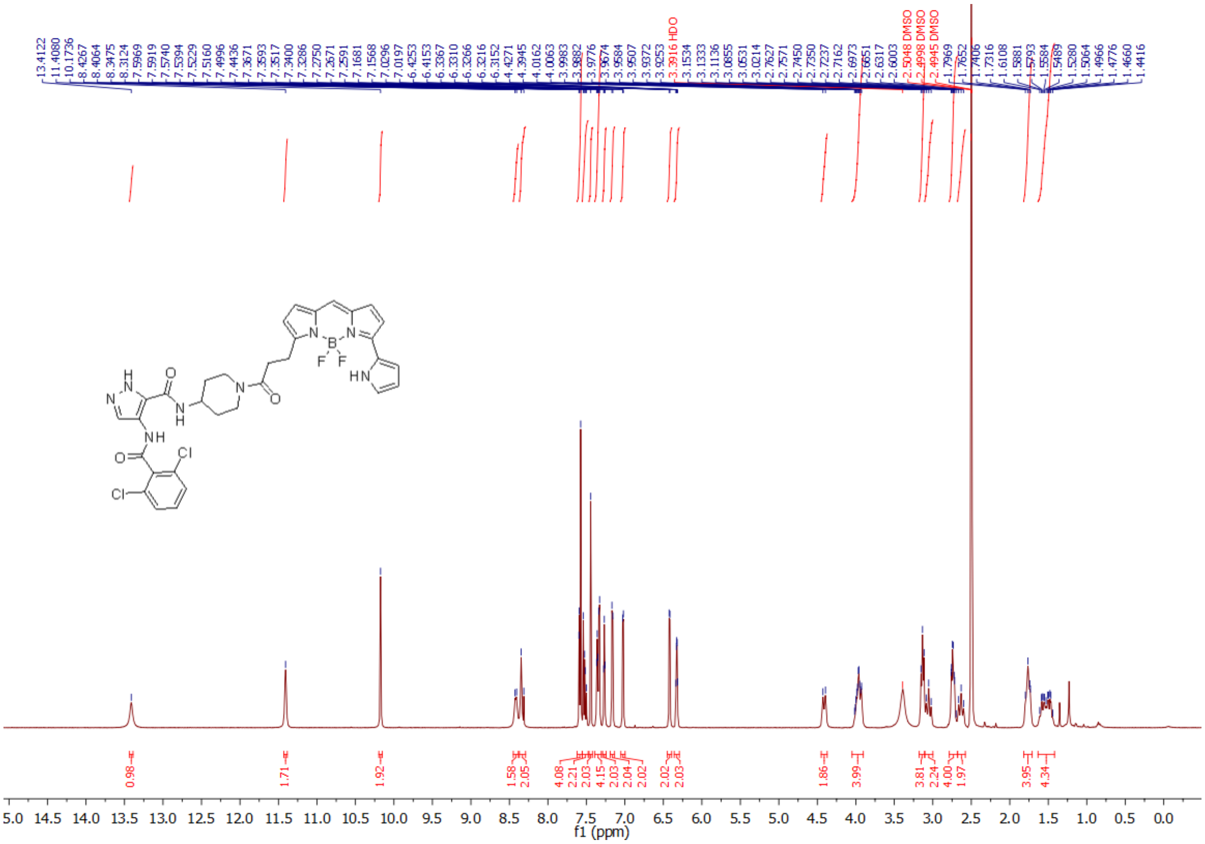

Supplementary Figure 31. 1H-NMR of probe 1.

Supplementary Figure 32

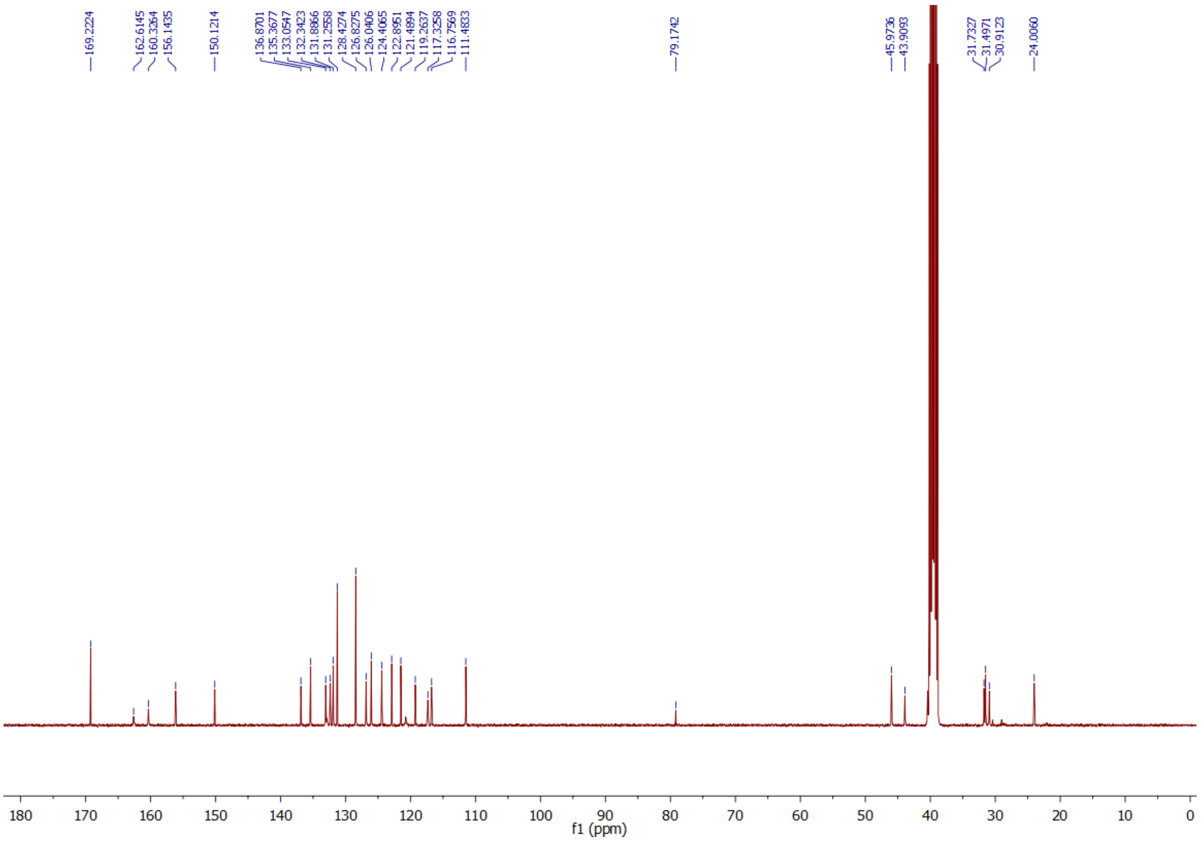

Supplementary Figure 32. ^{13}C-NMR of probe 1.

Supplementary Tables

Supplementary Table 1

CDK	[a]Relative BRET Probe 1	[a]Relative BRET Probe 2	[a]Relative BRET Probe 3	[a]Relative BRET Probe 4	[a]Relative BRET Probe 5
CDK1 + Cyclin E1	2.04	1.08	1.23	3.10	2.46
CDK2 + Cyclin E1	5.37	1.04	2.45	8.83	11.56
CDK3 + Cyclin E1	5.25	1.08	1.55	9.16	5.63
CDK4 + Cyclin D1	4.34	5.53	1.43	1.37	4.30
CDK5 + CDK5R1	4.60	1.09	1.80	5.55	9.46
CDK6 + Cyclin D1	3.05	4.09	1.18	1.46	3.14
CDK7	4.36	1.03	1.76	1.33	3.21
CDK8 + Cyclin C	1.22	1.04	5.08	1.08	1.07
CDK9 + Cyclin K	8.50	1.63	8.18	3.53	3.74
CDK10 + Cyclin L2	2.48	1.03	1.15	1.43	2.67
CDK11A + Cyclin L2	5.38	1.05	1.14	1.06	1.14
CDK11B + Cyclin L2	5.85	1.06	1.05	1.03	1.13
CDK12 + Cyclin K	1.25	1.03	1.21	1.39	1.44
CDK13 + Cyclin K	1.29	1.01	1.17	1.29	1.29
CDK14 + Cyclin Y	5.96	1.25	2.18	1.26	3.00
CDK15 + Cyclin Y	5.69	1.09	2.51	1.74	3.31
CDK16 + Cyclin Y	5.63	1.19	6.40	2.21	4.83
CDK17 + Cyclin Y	6.27	1.32	8.31	2.21	6.44
CDK18 + Cyclin Y	8.23	1.23	6.22	3.25	8.15
CDK19 + Cyclin C	1.13	1.05	2.37	1.02	1.06
CDK20 + Cyclin H	1.20	1.05	0.91	2.93	5.61

[a]Relative BRET signals for each CDK/NanoLuc fusion after incubation with optimized energy transfer probes. Each probe was screened at a concentration of 0.5µM in the presence or absence of 20µM unlabeled parent compound, and the relative BRET signal calculated as described in the legend of Supplementary Figure 1. Values represent a single biological experiment (n = 1) with technical replicates (n = 2) for both the tracer only samples and the samples with 20µM of the unlabeled parent compound. Source data are provided as a Source Data File.

Supplementary Table 2

Kinase	CDK Protein ID	Nluc Orientation	Cyclin/Regulator	Regulator Protein ID	Probe	[Probe], μM	[A]Z'
**CDK1**	NP_001307847	C	Cyclin E1	NP_001229	4	0.66	0.74
**CDK2**	NP_001789	C	Cyclin E1	NP_001229	5	0.5	0.87
**CDK3**	NP_001249	C	Cyclin E1	NP_001104515	4	0.66	0.96
**CDK4**	NP_000066	N	Cyclin D1	NP_444284	2	0.063	0.88
**CDK5**	NP_004926	C	CDK5R1	NP_003876	1	0.13	0.80
**CDK6**	NP_001138778	N	Cyclin D1	NP_444284	2	0.063	0.95
**CDK7**	NP_001790	N	None	N/A	5	0.5	0.84
**CDK8**	NP_001251	N	Cyclin C	NP_005181	3	0.063	0.90
**CDK9**	NP_001252	N	Cyclin K	NP_001092872	3	0.063	0.74
**CDK10**	NP_443714	C	Cyclin L2	NP_001034666	1	0.25	0.51
**CDK11A**	NP_001300825	C	Cyclin L2	NP_001034666	1	0.13	0.40
**CDK11B**	NP_277021	C	Cyclin L2	NP_001034666	1	0.13	0.87
**CDK12**	NP_055898	C	Cyclin K	NP_001092872	1	0.5	0.55
**CDK13**	NP_112557	C	Cyclin K	NP_001092872	1	0.5	0.42
**CDK14**	NP_001274064	C	Cyclin Y	NP_659449	1	0.13	0.64
**CDK15**	NP_001353315	N	Cyclin Y	NP_659449	1	0.063	0.71
**CDK16**	NP_006192	C	Cyclin Y	NP_659449	1	0.13	0.71
**CDK17**	NP_002586	C	Cyclin Y	NP_659449	1	0.13	0.74
**CDK18**	NP_002587	C	Cyclin Y	NP_659449	1	0.13	0.52
**CDK19**	NP_055891	N	Cyclin C	NP_005181	3	0.063	0.87
**CDK20**	NP_001034892	N	Cyclin H	NP_001230	4	0.66	0.75

[A]Z' values were measured in a single biological experiment (n = 1) with technical quadruplicates (n = 4) at the probe concentration specified above in the presence or absence of a saturating (≥10 μM) dose of unlabeled derivative.

Supplementary References

1 Robers, M. B. *et al.* Quantitative, Real-Time Measurements of Intracellular Target Engagement Using Energy Transfer. *Methods in molecular biology* **1888**, 45-71, doi:10.1007/978-1-4939-8891-4_3 (2019).

2 Dixon, A. S. *et al.* NanoLuc Complementation Reporter Optimized for Accurate Measurement of Protein Interactions in Cells. *ACS chemical biology* **11**, 400-408, doi:10.1021/acschembio.5b00753 (2016).

3 Schwinn, M. K. *et al.* CRISPR-Mediated Tagging of Endogenous Proteins with a Luminescent Peptide. *ACS chemical biology* **13**, 467-474, doi:10.1021/acschembio.7b00549 (2018).

4 Riching, K. M. *et al.* Quantitative Live-Cell Kinetic Degradation and Mechanistic Profiling of PROTAC Mode of Action. *ACS chemical biology* **13**, 2758-2770, doi:10.1021/acschembio.8b00692 (2018).

5 Hwang, B. B., Engel, L., Goueli, S. A. & Zegzouti, H. A homogeneous bioluminescent immunoassay to probe cellular signaling pathway regulation. *Commun Biol* **3**, 8, doi:10.1038/s42003-019-0723-9 (2020).
