# Supplementary material for: Quantifying CDK inhibitor selectivity in live cells
Source: Nat Commun. 2020 Jun 2;11:2743. doi: 10.1038/s41467-020-16559-0 (PMC7265341; doi:10.1038/s41467-020-16559-0)
Supplement: Supplementary file 3 — Reporting Summary [file 41467_2020_16559_MOESM3_ESM.pdf]

nature research

Corresponding author(s): Matthew Robers and Timothy Willson

Last updated by author(s): 23-April-2020

Reporting Summary

Nature Research wishes to improve the reproducibility of the work that we publish. This form provides structure for consistency and transparency in reporting. For further information on Nature Research policies, see Authors & Referees and the Editorial Policy Checklist.

Statistics

For all statistical analyses, confirm that the following items are present in the figure legend, table legend, main text, or Methods section.

n/a	Confirmed	
☐	☒	The exact sample size (n) for each experimental group/condition, given as a discrete number and unit of measurement
☐	☒	A statement on whether measurements were taken from distinct samples or whether the same sample was measured repeatedly
☒	☐	The statistical test(s) used AND whether they are one- or two-sided<br>*Only common tests should be described solely by name; describe more complex techniques in the Methods section.*
☒	☐	A description of all covariates tested
☒	☐	A description of any assumptions or corrections, such as tests of normality and adjustment for multiple comparisons
☐	☒	A full description of the statistical parameters including central tendency (e.g. means) or other basic estimates (e.g. regression coefficient) AND variation (e.g. standard deviation) or associated estimates of uncertainty (e.g. confidence intervals)
☒	☐	For null hypothesis testing, the test statistic (e.g. F, t, r) with confidence intervals, effect sizes, degrees of freedom and P value noted<br>*Give P values as exact values whenever suitable.*
☒	☐	For Bayesian analysis, information on the choice of priors and Markov chain Monte Carlo settings
☒	☐	For hierarchical and complex designs, identification of the appropriate level for tests and full reporting of outcomes
☒	☐	Estimates of effect sizes (e.g. Cohen's d, Pearson's r), indicating how they were calculated

Our web collection on statistics for biologists contains articles on many of the points above.

Software and code

Policy information about availability of computer code

Data collection	No software was used
Data analysis	Curve fitting was performed with Graphpad Prizm v8.4

For manuscripts utilizing custom algorithms or software that are central to the research but not yet described in published literature, software must be made available to editors/reviewers. We strongly encourage code deposition in a community repository (e.g. GitHub). See the Nature Research guidelines for submitting code & software for further information.

Data

Policy information about availability of data

All manuscripts must include a data availability statement. This statement should provide the following information, where applicable:

- Accession codes, unique identifiers, or web links for publicly available datasets
- A list of figures that have associated raw data
- A description of any restrictions on data availability

The source data underlying FIgures 2-5 and Supplementary figures S1-S10 and S12-S23 are provided as a source data file. All other data can be provided by the corresponding authors upon reasonable request.

Field-specific reporting

Please select the one below that is the best fit for your research. If you are not sure, read the appropriate sections before making your selection.

☒ Life sciences ☐ Behavioural & social sciences ☐ Ecological, evolutionary & environmental sciences

Life sciences study design

All studies must disclose on these points even when the disclosure is negative.

Sample size	This is not a clinical study, rather an assay and pharmacology resource. We feel that sample size relates to the number of inhibitors used in the study and the criteria for selection. The number of CDK inhibitors was selected (46) based on previous biochemical annotation and commercial availability.
Data exclusions	No data were excluded from the analysis
Replication	The objective of the study was to report potency values for certain CDK-inhibitor interactions. The values reported in the main body of the manuscript were repeated three times. These experiments involved three scientists and two different locations. Potency values +/- SD are reported.
Randomization	The study was intended to be a resource for those interested in evaluating CDK-inhibitor interactions in cells. The resulting data was intended to be a resource to guide the field on intracellular selectivity patterns for certain compounds. The authors feel that randomization was not necessary to achieve these goals.
Blinding	The scientists performing experiments were not blinded, since the purpose of the study was to generate a resource and template to assay CDKs in cells. However, technicians executing the potency experiments were uninformed as to the putative targets of the CDK inhibitors used in the study. Each had no a priori knowledge of the compound characteristics nor what should be "expected" in the profiling experiments reported herein.

Reporting for specific materials, systems and methods

We require information from authors about some types of materials, experimental systems and methods used in many studies. Here, indicate whether each material, system or method listed is relevant to your study. If you are not sure if a list item applies to your research, read the appropriate section before selecting a response.

Materials & experimental systems

n/a	Involved in the study	
☐	☒	Antibodies
☐	☒	Eukaryotic cell lines
☒	☐	Palaeontology
☒	☐	Animals and other organisms
☒	☐	Human research participants
☒	☐	Clinical data

Methods

n/a	Involved in the study	
☒	☐	ChIP-seq
☒	☐	Flow cytometry
☒	☐	MRI-based neuroimaging

Antibodies

Antibodies used	The following antibodies were purchased from Cell Signaling Technology Inc. and were used at 150 ng/ml: Rabbit anti-phospho-Rb (#8516) and Mouse anti-Rb (#9309)
Validation	Cell Signaling Technologies provides validation data for all antibodies

Eukaryotic cell lines

Policy information about cell lines

Cell line source(s)	HEK293 cells and MCF7 cells were from ATCC
Authentication	The cell lines were not authenticated beyond what is performed at ATCC
Mycoplasma contamination	The mycoplasma contamination was not determined beyond what is reported by ATCC
Commonly misidentified lines (See ICLAC register)	There were no commonly misidentified cell lines used in this study
