# Supplementary material for: Quantifying CDK inhibitor selectivity in live cells
Source: Nat Commun. 2020 Jun 2;11:2743. doi: 10.1038/s41467-020-16559-0 (PMC7265341; doi:10.1038/s41467-020-16559-0)
Supplement: Supplementary file 4 — Description of Additional Supplementary Files [file 41467_2020_16559_MOESM4_ESM.pdf]

Description of Additional Supplementary Files

File name: Supplementary Data 1
Description: Annotation of CDK inhibitors tested in this study

File name: Supplementary Data 2
Description: CDK inhibitor occupancy and potency across the 21 CDK assays

File name: Supplementary Data 3
Description: Mean potency of CDK inhibitors across the 21 CDK assays

Draft Only
