# Supplementary material for: Quantifying CDK inhibitor selectivity in live cells
Source: Nat Commun. 2020 Jun 2;11:2743. doi: 10.1038/s41467-020-16559-0 (PMC7265341; doi:10.1038/s41467-020-16559-0)
Supplement: Supplementary file 2 — Peer Review File [file 41467_2020_16559_MOESM2_ESM.pdf]

Reviewers' comments:

Reviewer #1 (Remarks to the Author):

Wells and Vasta et al., describe new assays for quantifying in vivo target engagement on CDK-cyclin complexes. For these studies, they have extended their development of BRET-based assays, previously developed for other enzymes or kinases (178 of the 500+) to all 21 human CDKs. Unlike their previous publications on BRET, they have gone much more in-depth on a single protein family and uncovered several interesting findings. For example, they identify several new and highly selective CDK inhibitors towards unique isozymes, including for the lesser studied CDKs including CDK 8 and 19. They also demonstrate an interesting comparison of compounds with similar potency for CDK6/2/7 using steady state analysis but marked variations in residence time based on wash-out protocols. Overall the findings in the manuscript are interesting and this report will likely become an important resource for investigators studying CDK-family members as drug targets. However, while the authors have produced an impressive toolkit to study CDKs, exploration into using tracers to learn more about basic kinase biology and pathology is missing, which would greatly increase the paper's impact. Specific concerns include:

1. Most IC50 data in the paper is reported as averages of duplicate runs. The variance in general looks acceptable for most interactions, however some outliers are notable. For example, there is a ~10-fold difference of 6 vs 63 nM for CDK16/Y and a BMS compound in supplementary table S4. Why did the authors not complete assays in biological triplicate and report IC50 values +/- s.d.? Triplicates and standard deviations should be reported for all IC50s in the main text and figures.

2. The authors show that BRET signals are modestly potentiated for the CDK vs CDK-cyclin complexes in Figure S3. Do the authors have any orthogonal data to support the formation of the intended complexes?...co-IP for example. Is there any chance that the tracers themselves can influence for the formation of CDK-cyclin complexes?

3. Determine IC50s for CDKs and probes in the presence and absence of cyclin expression (referencing Figure S3). Acquiring dose response curves is a better way of measuring changes that could be associated with cyclin binding.

4. Can the authors quantify residence time as a half-life value (ie. t1/2) for the inhibitors shown in Figure 5B?

5. Can the authors perform the reverse of the washout experiment (or other experiments) to determine a kon that would explain the difference of koff that give identical binding affinities in figure 5?

6. The sentence on line 129 of the paper is an overstatement, the authors should dial-back overly verbose language.

7. Is there a reference for the sentence on line 119 of the paper?

8. There is a type on line 287 – should be 'DFG-out'.

Reviewer #2 (Remarks to the Author):

Wells et al. report about the development of cell-permeable energy transfer probes to investigate intracellular potency and selectivity of 46 clinically-advanced CDK inhibitors against 21 CDKs. The

authors used a previously established technology by the same group to apply it to a broad-based CDK investigative study to correlate in vitro binding kinetics to live cell occupancy which is novel to the field. The paper is well written, the methodologies applied are highly advanced, the data interpretation is thorough, and the conclusions drawn are supported by the data. To my knowledge, this is the most comprehensive study on CDK inhibitors conducted to date. The results are convincing and will undoubtedly have a significant impact on the research community, e.g. rationalizing which drug should be used to probe specific CDKs in diseased cells, interrogate underexplored CDKs, repurpose CDK drugs in clinical development.

Minor points:
1. Line 150: "However, abemaciclib also showed collateral engagement of CDK2, -7, -9, and exhibited pan-TAIRE family activity (CDK14, -16-18), (Figure 2B)". This activity is not apparent from Fig. 2, which shows no collateral engagement with CDK2 and CDK7.
2. Please discuss the rationale behind the cyclin chosen to be co-expressed. Why was cyclin E1 co-expressed with CDK1 instead of cyclin A or cyclin B. Did the data reveal or hint if different cyclins affect intracellular occupancy (i.e. at different stages of the cell cycle)?
3. How do BRET data between N-terminal fusion vs C-terminal fusion proteins compare?

Reviewer #3 (Remarks to the Author):

The approaches and findings of Wells et al. are incredibly important to the CDK community as the lack of inhibitor specificity has plagued this field. I recommend this manuscript for publication with some revisions.

Major points:

1. The referee understands the authors' point that direct measurement of target engagement is more precise than inferring it from measuring phosphorylation of canonical downstream substrates. However, it might be informative to show that target engagement increases commensurate with decrease in phosphorylation of downstream substrates. This would demonstrate that the concentrations leading to target engagement are relevant to cellular effects elicited by compounds. Can the authors show side-by-side cellular target engagement and substrate inhibition for a 1-2 representative CDKs with well-defined substrates, or alternatively show concurrent effects on some cellular process, e.g. - cell cycle by FACS?

2. The authors nicely demonstrate that these assays can be run in HTS mode with transient transfection of NanoLuc-tagged CDKs. Can the authors demonstrate, or minimally address in the discussion, the possibility of using NanoBRET in cell lines where the CDK has been HiBit tagged on-genome at the endogenous locus. Presumably orthogonal expression of LgBit to reconstitute NanoLuc on the HiBit-tagged CDK would allow you to follow target engagement of the CDK expressed at endogenous levels.

3. The referee notices that there were no compounds with confirmed activity against CDK13, and very few with confirmed activity against CDK12, despite there being numerous compounds known to target CDK12 and CDK13 (e.g. - THZ1, THZ531, dinaciclib, flavopiridol). Can the authors comment on this? There are no Z' values shown for these (and other) CDKs, does this speak to the robustness of these particular assays? and thus the ability to identify compounds that hit these CDKs?

4. How are EC50 values determined in Figure S2? There are several curves where the BRET signal would seem to be leveling off and therefore an EC50 value could be identified as the concentration needed to get to 50% of BRETmax signal. However, there are several instances (e.g. CDK12, CDK13, CDK3, etc.) where the EC50 values can't be determined and are given as > 1 micromolar. Can the

authors explain this.

5. Related to question above, the caption of Figure S2 says that for some CDK assays (with EC50s > 1 micromolar) the BRET signal could not be saturated before hitting the solubility limit of the energy probes. How is saturated BRET signal (or BRETmax signal) determined?

Minor points:

1. For those readers not familiar with how BRET signal is calculated it would be nice to include the equation for easy reference.

2. Line 32-33. CDK7 is known to regulate the eurkaryotic cell cycle and should be included in list of CDKs that regulate this process. Numerous references could be made, including Robert Fisher’s bumped hole inhibitor papers.

3. Line 139, should be “…50% at 10 uM (micromolar)”, not 10 mM

Response to Reviewers

Reviewers' comments:

Reviewer #1 (Remarks to the Author):

Wells and Vasta et al., describe new assays for quantifying in vivo target engagement on CDK-cyclin complexes. For these studies, they have extended their development of BRET-based assays, previously developed for other enzymes or kinases (178 of the 500+) to all 21 human CDKs. Unlike their previous publications on BRET, they have gone much more in-depth on a single protein family and uncovered several interesting findings. For example, they identify several new and highly selective CDK inhibitors towards unique isozymes, including for the lesser studied CDKs including CDK 8 and 19. They also demonstrate an interesting comparison of compounds with similar potency for CDK6/2/7 using steady state analysis but marked variations in residence time based on wash-out protocols. Overall the findings in the manuscript are interesting and this report will likely become an important resource for investigators studying CDK-family members as drug targets. However, while the authors have
produced an impressive toolkit to study CDKs, exploration into using tracers to learn more about basic kinase biology and pathology is missing, which would greatly increase the paper's impact. Specific concerns include:

1. Most IC50 data in the paper is reported as averages of duplicate runs. The variance in general looks acceptable for most interactions, however some outliers are notable. For example, there is a ~10-fold difference of 6 vs 63 nM for CDK16/Y and a BMS compound in supplementary table S4. Why did the authors not complete assays in biological triplicate and report IC50 values +/- s.d.? Triplicates and standard deviations should be reported for all IC50s in the main text and figures.

Author Response: The authors agree that it is important to provide IC50 values as an average of 3 replicates +/- error.

Modification to Manuscript: All of the compound IC50s reported in the main manuscript have been repeated. Values are now reported as IC50 +/- S.E.M. of three experimental replicates, and provided in the tables and supplemental tables S4 and S5.

2. The authors show that BRET signals are modestly potentiated for the CDK vs CDK-cyclin complexes in Figure S3. Do the authors have any orthogonal data to support the formation of the intended complexes?...co-IP for example. Is there any chance that the tracers themselves can influence for the formation of CDK-cyclin complexes?

Author Response: This was an extremely valuable recommendation. Authors agree that the assay conditions utilized promote formation of the intended CDK/cyclin interaction. For this purpose, we designed a novel assay configuration (using NanoBiT), wherein luminescence is conditional upon formation of the intended CDK-cyclin interaction. Furthermore, the authors explored the influence of the CDK-cyclin complex on engagement pharmacology with known CDK inhibitor. To the authors' knowledge, this configuration represents a novel demonstration that target engagement can be quantified at selected protein complexes using a complementation based system. Authors feel this

addition to the paper may warrant a stronger "feature" in the main body of the manuscript, and will seek editorial input on changing the main body of the manuscript to accommodate an illustration (with discussion) on this novel assay design. This is an impactful manuscript modification and the authors are grateful that the Reviewer response spurred us to evaluate this concept. This new NanoBiT target engagement design represents a new direction for assay development, likely warranting a follow-up publication. For example, this NanoBiT method could allow for the identification of compounds that stabilize monomeric CDKs via disruption of cyclin complexation.

Modification to manuscript: To demonstrate that the assay conditions utilized promote formation of the intended CDK/cyclin interaction, a complementation system (NanoLuc Binary Technology, aka NanoBiT) was developed using a "split" form of NanoLuc, wherein the small subunit of Nluc (SmBiT) was fused to the CDK2, and the large subunit of NanoLuc (LgBiT) was fused to the cyclinE1. Co-expression of the NanoBiT constructs was performed under conditions identical to the original assay design (CDK2-NanoLuc + untagged cyclinE1). Upon coexpression of the binary CDK2-cycE1 NanoBiT system, a > 100 fold increase in luminescence was observed in the presence of CDK-SmBiT and cyclin-LgBiT (versus negative control expressed at comparable level). Furthermore, the authors demonstrate that the conditional binary luminescent (CDK-cyclin) complex was viable for energy transfer with the BRET probe. To the authors' knowledge, this complementation-based assay design represents the first method to measure target engagement selectively at protein-protein complexes in cells. When target engagement IC50 was compared between the binary reporter, and the original (NanoLuc) design, compound IC50 was nearly identical. In contrast, when CDK-NanoLuc was expressed in absence of cyclin, the IC50 of test compound was shifted nearly 2 orders of magnitude. These results strongly support formation of the intended CDK-cyclin interaction. These new results are presented in figures S11-S12, and discussed in the last paragraph of the first results section.

3. Determine IC50s for CDKs and probes in the presence and absence of cyclin expression (referencing Figure S3). Acquiring dose response curves is a better way of measuring changes that could be associated with cyclin binding.

Author response: The authors agree that a dose response is more valuable in this case.

Modification to manuscript: All CDK target engagement assays to evaluate the impact of cyclin co-expression were repeated as a probe dose response and provided in the supplementary Figure S8.

4. Can the authors quantify residence time as a half-life value (ie. t1/2) for the inhibitors shown in Figure 5B?

Author Response: This is a valuable recommendation. The authors recognize the value in quantifying compound off-rate in this context. The authors plotted a non-linear regression analysis on the residence time data for CDK6, CDK2, and CDK7. One phase association kinetics were measured for CDK2 and CDK7 (residence time, t, = 41 min and 8 minutes, respectively). This information is now provided in the final paragraph of the results section. However, as the occupancy of RGB2866638 for CDK6 was still very strong after two hours of analysis. This long residence time was difficult to quantify based on the curve fit. The authors considered extending the analysis to multiple hours, but were concerned about the interpretation of data at long incubations (i.e., after target turnover and cellular health could become compromised). Therefore, the authors emphasized

the work flow described below in question #5, and also added a discussion on the topic of intracellular compound residence time (and possible impacts of cellular portioning/rebinding) on the observations. Please see the final results section, final paragraph for modifications. The authors expound on this topic in response to question #5 below.

5. Can the authors perform the reverse of the washout experiment (or other experiments) to determine a kon that would explain the difference of koff that give identical binding affinities in figure 5?

Author Response: The authors agree that additional analyses are valuable to demonstrate that slow on/off rates are observed (as would be expected) with CDK6. Therefore, the authors designed a work flow to measure compound k_{obs} *as a function of compound dose, with the expectation that slow equilibration would be evident for CDK6 compared to CDK7.*

Modification to manuscript: The authors designed a real-time experiment to evaluate the equilibration rate of RGB286638 with BRET. When CDK6 and CDK7 were compared, slower k_{obs} *and also rate of IC50 stabilization was observed for CDK6 (versus CDK7), as expected. These results shown in figure S23 support the protracted residence time observed for CDK6 vs CDK7 in cellular washout studies. The authors recognize that quantitative metrics are valuable, however it should also be recognized that a number of cellular factors may influence the observed rates of compound engagement (e.g. permeability, partitioning, target turnover, etc). The authors did measure the off rate for CDK6 using the* k_{obs} *values, and observed a value >300 minutes. Although this value corroborates the cellular washout study, the authors are hesitant to report such values based on the possible impacts of cell physiology mentioned above. The authors therefore modified the manuscript with the new kinetic data corroborating the washout results (figure S23), and included a discussion (final paragraph of results section) on the potential impacts of cellular rebinding (including appropriate references) and possible limitations to quantify specific on/off rates in cells using this method. The authors recognize that cell-free biophysical approaches are warranted as an extension of this study to achieve such quantitative metrics.*

6. The sentence on line 129 of the paper is an overstatement, the authors should dial-back overly verbose language.

Author response: Line 129 in the submitted version appears as; "Despite the literature being rife with CDK inhibitors, very few CDKi's have been comprehensively profiled against the full CDK family in any assay format.17,18,20 " The authors agree that this may be an overstatement.

Modification to manuscript: The authors have modified this sentence accordingly.

7. Is there a reference for the sentence on line 119 of the paper?

Author response: 119 reads as "The enzymatic activity of many CDKs was known to be modulated in cells by the specific cyclin or regulatory partner to which they are complexed."

Modification to manuscript: A reference has now been included.

8. There is a type on line 287 – should be 'DFG-out'.

Author response: Line 287 reads as "Further evaluation is therefore warranted to determine if K03861 engages CDK8/19 via type II "DMG-out" mechanism, and if type II inhibitors could be a productive strategy for inhibition of mediator kinase activity" The authors agree this should be clarified. To the authors knowledge, this motif is indeed DMG (methionine) for CDK8, and not DFG based on Dale et al. Nat Chem Bio, 2019 / doi: 10.1038/nchembio.1952.

Modification to manuscript: The authors have deleted this term as it may be confusing because the field likely recognizes the DFG-out terminology as convention. The F→M variation is non critical to this point in the manuscript and has been removed.

Reviewer #2 (Remarks to the Author):

Wells et al. report about the development of cell-permeable energy transfer probes to investigate intracellular potency and selectivity of 46 clinically-advanced CDK inhibitors against 21 CDKs. The authors used a previously established technology by the same group to apply it to a broad-based CDK investigative study to correlate in vitro binding kinetics to live cell occupancy which is novel to the field. The paper is well written, the methodologies applied are highly advanced, the data interpretation is thorough, and the conclusions drawn are supported by the data. To my knowledge, this is the most comprehensive study on CDK inhibitors conducted to date. The results are convincing and will undoubtedly have a significant impact on the research community, e.g. rationalizing which drug should be used to probe specific CDKs in diseased cells, interrogate underexplored CDKs, repurpose CDK drugs in clinical development.

Minor points:
1. Line 150: "However, abemaciclib also showed collateral engagement of CDK2, -7, -9, and exhibited pan-TAIRE family activity (CDK14, -16-18), (Figure 2B)". This activity is not apparent from Fig. 2, which shows no collateral engagement with CDK2 and CDK7.

Author response: The authors recognize this mistake in the main text. The figures were correct but the text was incorrect.

Modification to manuscript: This text has been revised accordingly.

2. Please discuss the rationale behind the cyclin chosen to be co-expressed. Why was cyclin E1 co-expressed with CDK1 instead of cyclin A or cyclin B. Did the data reveal or hint if different cyclins affect intracellular occupancy (i.e. at different stages of the cell cycle)?

Author response: The authors agree that more discussion on the selection of the cyclins is warranted.

Modification to manuscript: The choice of cyclin can dramatically impact engagement pharmacology for some CDKs in this live cell assay format. This has been evaluated extensively by the authors with

CDK2. Additional data evaluating CDK2 engagement with various cycE, cycA, and empty plasmid (no cyclin) were included (figure S10), as well as a new assay design using NanoBiT-based complementation to prove CDK2/cyclinE1 interactions (figures S11-S12). The CDK2 results strongly support that engagement pharmacology is impacted by cyclin coexpression. The methodological template should allow for alternate cyclin/CDK interactions to be queried in this format. The authors have also modified the text to emphasize the possibility that alternate cyclins may be valuable using the experimental template provided (results section 1, paragraph 4).

3. How do BRET data between N-terminal fusion vs C-terminal fusion proteins compare?

Author response: The authors agree that the difference in BRET data using N vs C terminally tagged Nluc should be discussed and example data presented. During the course of assay development all CDK/Nluc orientations were evaluated to determine the optimal orientation for energy transfer. Much of these data were collected with earlier generations of energy transfer probes. This data can be provided if necessary. However, the authors feel that example data for a subset of CDKs will be sufficient to illustrate this point. If data on all CDKs is requested, this could be provided.

Modification to manuscript: The authors have included additional text and data illustrating how Nluc tagging orientation can impact the BRET signal (figure S6). This result is expected based on the impacts of geometry and distance on BRET efficiency.

Reviewer #3 (Remarks to the Author):

The approaches and findings of Wells et al. are incredibly important to the CDK community as the lack of inhibitor specificity has plagued this field. I recommend this manuscript for publication with some revisions.

Major points:

1. The referee understands the authors’ point that direct measurement of target engagement is more precise than inferring it from measuring phosphorylation of canonical downstream substrates. However, it might be informative to show that target engagement increases commensurate with decrease in phosphorylation of downstream substrates. This would demonstrate that the concentrations leading to target engagement are relevant to cellular effects elicited by compounds. Can the authors show side-by-side cellular target engagement and substrate inhibition for a 1-2 representative CDKs with well-defined substrates, or alternatively show concurrent effects on some cellular process, e.g. - cell cycle by FACS?

Author response: This is a valuable recommendation. The authors agree that evaluating target engagement and its relationship with functional inhibition is highly valuable.

Modification to manuscript: The authors utilized a recently reported homogenous immunoassay method to quantify the pharmacological inhibition of CDKs at known intracellular substrate (Rb).

Here, the authors utilized a new complementation-based homogenous immunoassay, wherein luminescence is generated in the presence of phosphorylated Rb from cell extracts. This method is capable of detecting phosphorylation of endogenous substrates such as Rb, and was successfully adapted to measure the potencies of CDK4/6-selective inhibitors in MCF7 breast cancer cells (figure S15). To directly compare target engagement with inhibition of phosho-Rb, the authors adapted both CDK4 and CDK6 energy transfer assays into the same MCF7 cell line. The authors demonstrate that target engagement at CDK6 strongly correlate with potency of functional inhibition using Rb phosphorylation (figure S15 and discussion in results section 3, paragraph 1). The authors feel this demonstration of the relationship between occupancy and functional inhibition represents a major improvement to the publication, and are interested in editorial input on inclusion of these data into the main body of the manuscript.

2. The authors nicely demonstrate that these assays can be run in HTS mode with transient transfection of NanoLuc-tagged CDKs. Can the authors demonstrate, or minimally address in the discussion, the possibility of using NanoBRET in cell lines where the CDK has been HiBit tagged on-genome at the endogenous locus. Presumably orthogonal expression of LgBit to reconstitute NanoLuc on the HiBit-tagged CDK would allow you to follow target engagement of the CDK expressed at endogenous levels.

Author response: The authors agree that a comparison of ectopically expressed CDK with CDK expressed at endogenous levels is warranted.

Modification to manuscript: The authors developed two cell lines endogenously edited with CRISPR/Cas9 to introduce HiBiT into endogenous genomic loci for CDK9 and CDK16. Target engagement assays were then performed on these modified cell lines for comparison to data generated with ectopically expressed CDK. Engagement potencies were comparable in the endogenous vs ectopic systems, supporting the capacity of the assays to match those of endogenously expressed targets (figure S17 and discussion in results section 3, paragraph 2).

3. The referee notices that there were no compounds with confirmed activity against CDK13, and very few with confirmed activity against CDK12, despite there being numerous compounds known to target CDK12 and CDK13 (e.g. - THZ1, THZ531, dinaciclib, flavopiridol). Can the authors comment on this? There are no Z' values shown for these (and other) CDKs, does this speak to the robustness of these particular assays? and thus the ability to identify compounds that hit these CDKs?

Author response: The authors agree and recognize that the CDK12 and CDK13 assays are less robust than the remaining CDKs, and that this should be pointed out as an opportunity for improvement. It is a noteworthy observation that THZ1, THZ531, dinaciclib, and flavopiridol did indeed engage both CDK12 and 13 in live cells in the primary screen at 10uM (yielding % occupancies close to 50% at 10uM). Furthermore for CDK12, the IC50 of THZ531 was in range for reporting (<1uM). However, in follow up IC50 profiling, the potency for the majority of these other compounds fell just outside of the hit criteria for reporting. The authors have followed up on THZ531 IC50 analysis for CDK13 and observed engagement with an IC50 of approximately 2uM (corroborating the primary screen results). For many of these compounds the failure to show potent engagement (<1uM) may be partially due to intracellular [ATP] or target activation state (which may deviate from cell free profiling results).

Modification to manuscript: the authors performed Z prime analysis across CDK assays and do observe weaker statistical performance for CDK12 and CDK13 (table S2). The authors also provide commentary on these specific assays and opportunity for improved energy transfer probes for these two CDKs (results section 1, paragraph 3). The authors included a discussion on the observation that THZ531 did engage CDK13 in our screen, but that the potency fell outside of our stringent hit criteria (<1uM) (discussion section, paragraph 4). The authors would be happy to include the follow up IC50 profiling on CDK13 with THZ531 if it would strengthen the manuscript. This value of THZ531 potency was approximately 2uM.

4. How are EC50 values determined in Figure S2? There are several curves where the BRET signal would seem to be leveling off and therefore an EC50 value could be identified as the concentration needed to get to 50% of BRETmax signal. However, there are several instances (e.g. CDK12, CDK13, CDK3, etc.) where the EC50 values can't be determined and are given as > 1 micromolar. Can the authors explain this.

Author Response: In general, EC50 values for probes were determined by fitting the sigmoidal dose-response equation to dose-dependent BRET values. In most cases, an EC50 can be determined before the probe concentration has reached its functional performance limit (typically 0.5-1uM for most of these probes (see question 5 below). In these cases, an EC50 value can be reported. In a few cases, there is false plateau observed due to approaching the functional performance limit. We depict those BRET values in the plots so that a user knows that there is no additional signal-window gained by going to higher concentrations. Still, we recognize that in those cases, we cannot report an EC50 value that is functionally meaningful for determining the probe concentration to use in the assay. Moreover, these assays are almost all operating at a subsaturating probe concentration. This can be determined by evaluating CDKi IC50 as a function of tracer concentration. When tracer concentrations exceed the Kd-apparent for the target protein, a right shifting would be evident based on the Cheng-Prusoff relationship (as demonstrated in by our group previously for kinases; doi: 10.1016/j.chembiol.2017). Authors demonstrate that BRET probes are operating at subsaturating concentrations, by evaluating the Cheng-Prusoff relationship between probe and control compound for each assay. Results demonstrate that the probes are non-saturable for some CDKs.

Modification to manuscript: To address the points above, we have revised supplementary figure S2 such that we report an EC50 of "undefined" in cases where we cannot confidently determine the probe EC50 due to the functional performance limit. We have also added two additional supplementary figures. The first depicts the effect of the probe concentration on the apparent IC50 of a test compound (figure S3). We include this data for all assays to demonstrate that we are operating at probe concentrations that only minimally influence IC50 (hence are subsaturating), which provides complimentary value to the probe EC50 data. The second figure (S4) provides linearized cheng-prusoff analyses for all of the assays where the probe EC50 is undefined. In most of these cases, we show that a meaningful cheng-prusoff relationship has not been established over the probe concentration ranges that were tested, providing further support that those assays are being performed at a subsaturating and quantitative probe concentration.

5. Related to question above, the caption of Figure S2 says that for some CDK assays (with EC50s >

1 micromolar) the BRET signal could not be saturated before hitting the solubility limit of the energy probes. How is saturated BRET signal (or BRETmax signal) determined?

Author Response: This is another great point related to question 4 above. We know from historical experience that most bodipy-based BRET probes like those reported here have functional performance limits in the 0.5-5µM range. This phenomenon is not exclusively solubility, but rather, likely has aggregation and/or plate adsorption components. Thus, it is not easily measurable via typical biophysical approaches. However, it is commonly observed as an abrupt deviation in linearity in the cheng-prusoff relationship for targets where probe-binding is saturable. Hence, we use cheng-prusoff approach as applied to the study of 3 of the energy transfer probes to provide evidence for that abrupt deviation from linearity, using targets for which probe binding is saturable (and thus demonstrate a cheng-prusoff relationship at lower probe concentrations).

Modification to manuscript: We have included an additional supplementary figure (S5) where we have taken 3 of the energy transfer probes and performed linearized cheng-prusoff analyses against kinase targets for which those probes are saturable. These targets are not necessarily CDKs, but demonstrate the phenomenon nonetheless. In these plots, we perform linear fits to the data over the linear and non-linear range to highlight the "breaking point" in the linear relationship, which signifies that the functional performance limited has been reached. It ranged from 0.5-1µM for these three probes studied here, well within expectations from our previous development experience with 100s of other bodipy-based BRET probes.

Minor points:

1. For those readers not familiar with how BRET signal is calculated it would be nice to include the equation for easy reference.

Author response: The authors agree that this should be stated, as not all readers are familiar with BRET.

Modification to manuscript: A statement was included to introduce how BRET is measured in a ratiometric format (results section 1, paragraph 2). The methods section currently includes the exact calculation and measurement conditions.

2. Line 32-33. CDK7 is known to regulate the eurkaryotic cell cycle and should be included in list of CDKs that regulate this process. Numerous references could be made, including Robert Fisher's bumped hole inhibitor papers.

Author response: The authors recognize this misstatement.

Modification to manuscript: A reference to Kwiatkowski et al has now been made and the role of CDK7 has been corrected.

3. Line 139, should be "…50% at 10 uM (micromolar)", not 10 mM

Author response: The authors recognize this mistake.

Modification to manuscript: The correction has been made.

REVIEWERS' COMMENTS:

Reviewer #1 (Remarks to the Author):

The authors have done a very nice job addressing concerns from the first submission. In particular, the addition of Nanobit is quite nice.

Minor correction: line 35 has a grammatical error with a period followed by a comma.

Optional issue to address: New residence time data (starting on line 287) was fit with a one-phase association equation in prism. If the authors have data showing these measurements at different drug concentrations, then they could extract kon and koff. Having these parameters in the manuscript would demonstrate that they can be measured.

Reviewer #3 (Remarks to the Author):

The authors did an excellent job addressing all reviewer questions. I fully support the publication of this manuscript with these new experimental data. I believe some of the new findings, particularly those addressing the reviewer questions below, to be worthy of inclusion in the main figures, but I leave it up to the editors and authors on how to address this:

Reviewer 1 - questions 2, 3
Reviewer 3 - questions 1, 2

Reviewer #1 (Remarks to the Author):

The authors have done a very nice job addressing concerns from the first submission. In particular, the addition of Nanobit is quite nice.

Minor correction: line 35 has a grammatical error with a period followed by a comma.

Author response: This error has been corrected in the revision.

Optional issue to address: New residence time data (starting on line 287) was fit with a one-phase association equation in prism. If the authors have data showing these measurements at different drug concentrations, then they could extract kon and koff. Having these parameters in the manuscript would demonstrate that they can be measured.

Author response: Per discussion with the Editor, this analysis will not be appropriate to determine kon and koff. Therefore, we would prefer not to provide additional experimental data.

Reviewer #3 (Remarks to the Author):

The authors did an excellent job addressing all reviewer questions. I fully support the publication of this manuscript with these new experimental data. I believe some of the new findings, particularly those addressing the reviewer questions below, to be worthy of inclusion in the main figures, but I leave it up to the editors and authors on how to address this:

Reviewer 1 - questions 2, 3
Reviewer 3 - questions 1, 2

Author response: Per guidance by the Editor, we have elected to keep these results in the Supplementary section.

Please do not hesitate to contact me with questions or requests for additional modifications.
